# Enantioselective 3,4-Oxyamination of 1,3-Dienes Enabled by an Electron-Rich Planar Chiral Rhodium Indenyl Catalyst

**DOI:** 10.1021/acs.orglett.5c02176

**Published:** 2025-06-30

**Authors:** Ethan M. P. Heyboer, Wesley A. Pullara, John Bacsa, Simon B. Blakey

**Affiliations:** Department of Chemistry, 1371 Emory University, Atlanta, Georgia 30322, United States

## Abstract

We describe the development of a three-component enantioselective 3,4-amino oxygenation protocol for 1,3-dienes enabled by a highly methylated electron-rich planar chiral rhodium indenyl catalyst. This transformation furnishes vicinal amino alcohol motifs in synthetically useful yields (19–86%) and excellent enantioselectivities (up to 99.5:0.5 er). We demonstrate its utility for a variety of activated and unactivated dienes coupled with a broad scope of dioxazolone and alcohol coupling partners.

## Graphical abstract

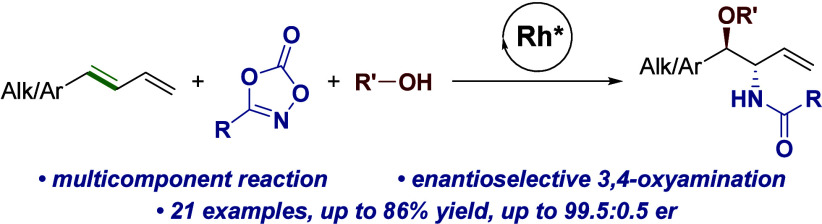

Vicinal amino alcohols have emerged as a privileged motif, appearing in a variety of natural products, pharmaceuticals, and ligands for asymmetric catalysis.^1^ Consequently, considerable efforts have been devoted toward the development of methods for rapid access to enantioenriched amino alcohols.^1,2^ Many of these strategies revolve around transition-metal-catalyzed oxidations of simple alkene precursors. However, these methods often require tethered functionalities and directing groups, complicating their synthetic utility.^3–11^ These considerations extend to the direct oxyamination of conjugated π systems such as 1,3-dienes, whose added alkene functionalities promise enhanced synthetic utility and more challenging reactivity in equal measure.^12,13^ A fully intermolecular three-component amino oxygenation of 1,3-dienes that did not require embedded directing groups or tethering would provide modular access to valuable enantioenriched amino alcohols. Recent work by Li et al.^14^ demonstrates the value of this approach in the context of *gem*-difluorodienes; however, this method cannot access vicinal amino alcohols, and its scope is constrained to aryl *gem*-difluorodiene substrates. Additional work by Chen et al.^15^ provides access to the vicinal amino alcohol motif, but only in the 1,2 fashion, and alkyl diene substrates suffer from diminished selectivities. These examples highlight the need for further development of this reactivity; in particular, an enantioselective 3,4-oxyamination of 1,3-dienes is, to date, unknown (Figure 1A).

**Figure 1:**
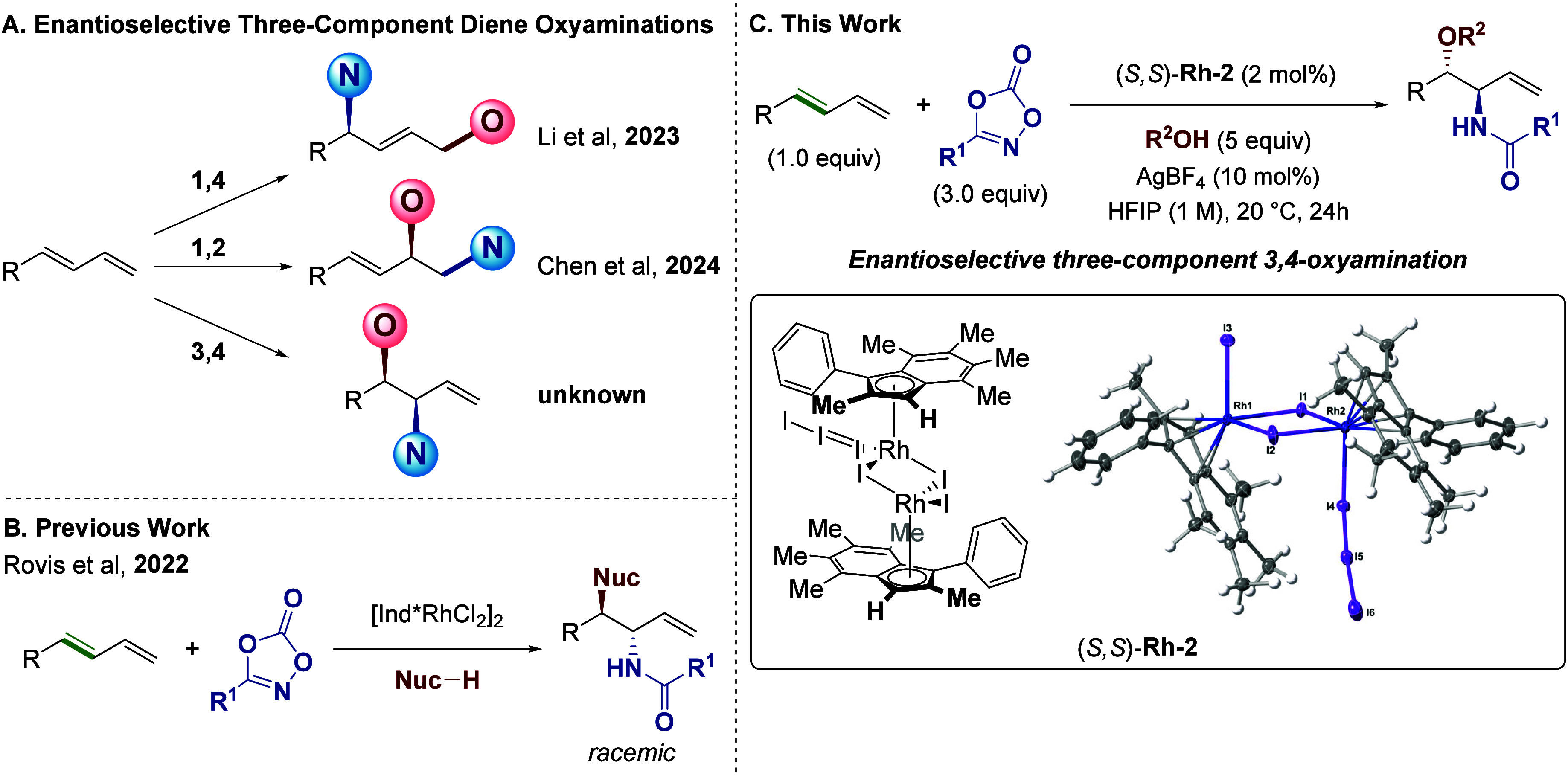
Overview of three-component diene oxyaminations.

To that end, and building from Rovis’ racemic method to access 3,4-amino alcohols via oxyamination of terminal 1,3-dienes (Figure 1B),^16,17^ we set out to develop an enantioselective version of this reaction through the use of our planar chiral Rh­(III) indenyl catalyst class. These catalysts have a demonstrated utility in enantioselective alkene functionalizations,^18–22^ and we hypothesized that the planar chirality of the catalyst would also provide stereocontrol in the initial outer-sphere nucleophilic attack of an alcohol nucleophile to a coordinated 1,3-diene. Herein, we report the successful realization of an enantioselective three-component 3,4-amino oxygenation of 1,3-dienes (Figure 1C).

Our investigation began with a brief exploration of planar chiral Rh­(III) indenyl catalysts (Figure 2, entries 1–2). We quickly discovered that the 2-Me-3-Ph indenyl catalyst **Rh-1**, which had been effective in promoting enantioselective allylic amidation^18^ and olefin aziridination reactions,^19^ did not provide the desired reactivity.

**Figure 2:**
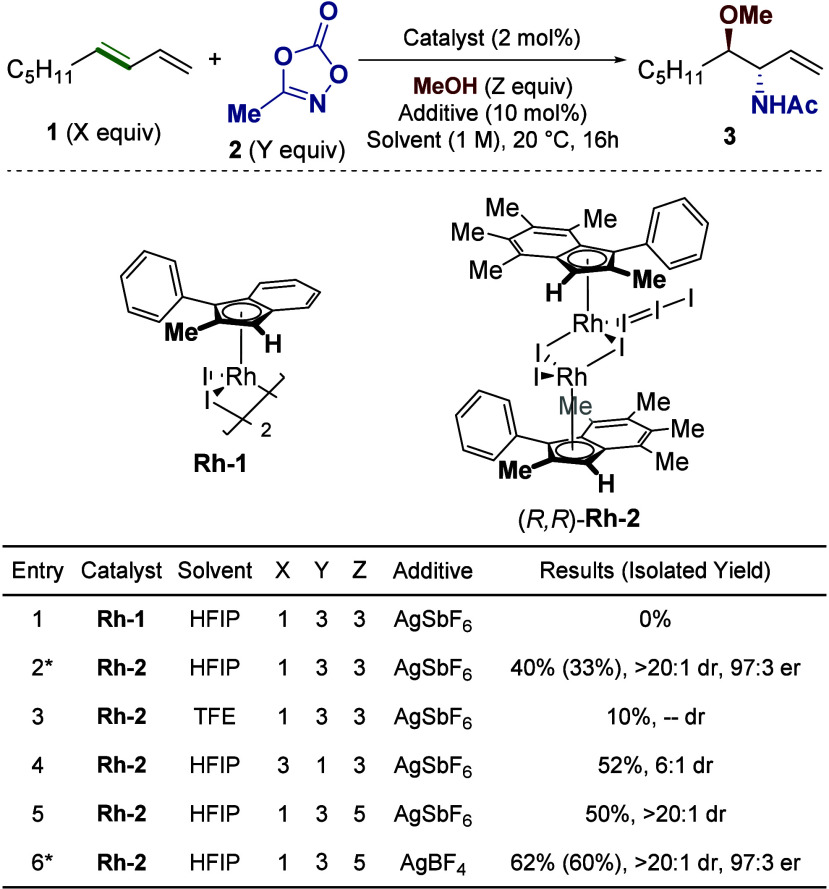
Optimization of the reaction. NMR yields and dr values were calculated using mesitylene as an internal standard, and er values were determined via chiral HPLC. * indicates use of (*R*,*R*)-**Rh-2**.

Considering the racemic reaction’s dependence on the permethylated [Ind*RhCl_2_]_2_ catalyst,^16^ we turned to a highly methylated version of our catalyst, **Rh-2**, which we were able to prepare in four steps from commercially available starting materials. The Rh­(I) precursor to **Rh-2** was resolvable via chiral HPLC (see Supporting Information), allowing for subsequent I_2_ oxidation of enantiopure material. Intriguingly, a crystal structure of this complex revealed that it bore an I_3_^–^ ligand as opposed to the traditional I^–^ ligand expected in Rh­(III) halide dimers (Figure 1C).^23^ Attempts to isolate the simple iodide analogue by performing the reaction with 1 equiv of I_2_ were not successful.

Regardless, the planar chiral complex (*R*,*R*)-**Rh-2** provided the desired oxyamination reactivity in modest yield (33%) but with excellent enantioselectivity (97:3 er), providing a starting point for further optimization initially conducted using a racemic mixture of **Rh-2** enantiomers.

The reaction demonstrated a significant dependence on HFIP as the solvent, with other fluorinated solvents providing poor conversion (Figure 2, entry 3). However, this posed a challenge regarding reactivity. Though it is necessary for the reaction to proceed, HFIP also causes decomposition of the nonadiene starting material **1**, likely through an acid-catalyzed polymerization pathway (see Supporting Information). An attempt to diminish the impact of this decomposition by using dioxazolone **2** as the limiting reagent improved yield, but at the cost of decreased diastereoselectivity (entry 4). We attribute this effect to a decrease in the rate of nitrene insertion in the mechanism (*vide infra*).^16^ In the interest of maintaining the stereoselective quality of the reaction, we chose to move forward using **1** as the limiting reagent. Increasing the amount of nucleophile used enhanced the yield (entry 5), and we found AgBF_4_ to be the most effective halide scavenger (entry 6). After confirming maintenance of the high enantioselectivity found in our initial hit, we moved forward with the conditions outlined in entry 6 (60% yield, >20:1 dr, 97:3 er).

With optimized conditions in hand, we turned our attention toward the application of this procedure to several diene substrates (Figure 3). The reaction conditions tolerate both electron-donating and -withdrawing groups distal to the diene (**5–7**), as well as a reactive halide functional handle (**8**). Aryl dienes are also tolerated, albeit with reduced yield and enantioselectivity (**9–10**). The reaction operates in an intramolecular fashion as well, with both alcohol and amine nucleophiles tolerated, though enantioselectivity is once again reduced (**11–12**). Finally, the reaction can be carried out on a larger scale, with product **4** obtained in an acceptable yield with excellent enantioselectivity on a 1.0 mmol scale.

**Figure 3:**
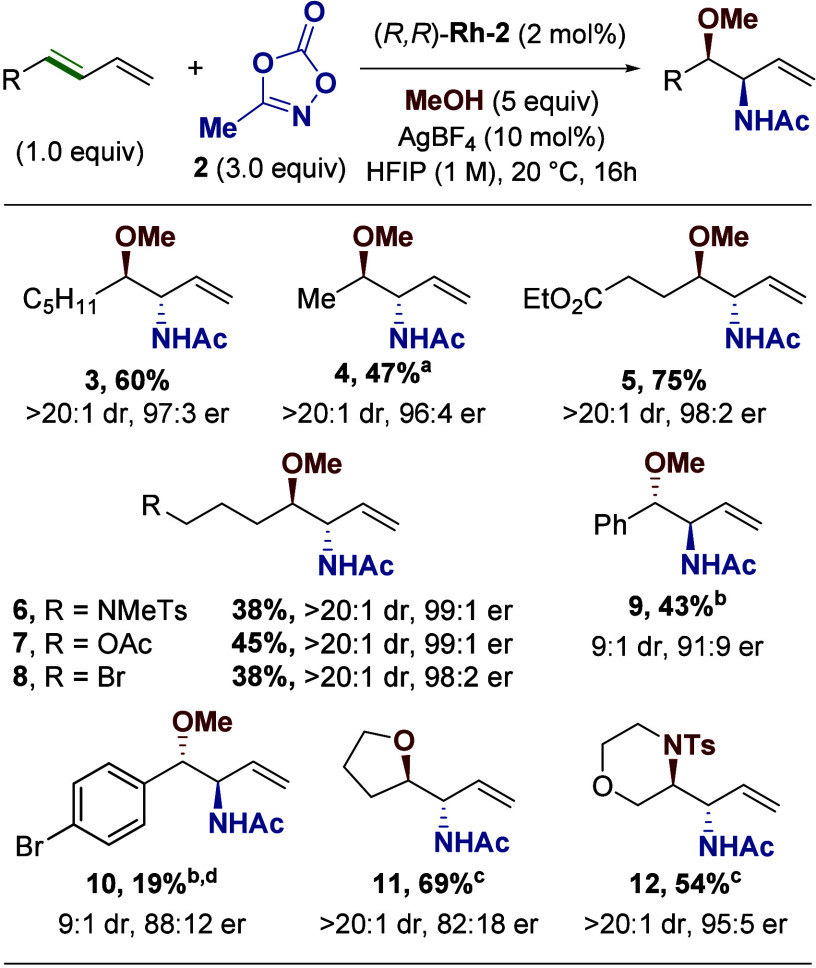
Scope of dienes. Reactions were run on a 0.25 mmol scale. Diastereomeric ratios were determined from crude NMR. Enantiomeric ratios were determined by chiral HPLC. ^a^Reaction was run on a 1.0 mmol scale. ^b^Reaction was run with (*S*,*S*) catalyst. ^c^Reaction was run without MeOH. ^d^The absolute stereochemistry of compound **10** was obtained via X-ray crystallography (see Supporting Information). All other stereochemical outcomes are assigned by analogy.

We next turned to the nucleophile and dioxazolone scope (Figure 4). Simple hydroxyl nucleophiles were tolerated (**14–15**), notably without competitive nucleophilic acyl substitution at the diene ester. The reaction also demonstrated tolerance of the oxidatively sensitive heterocycles furan (**16**) and thiophene (**17**), as well as a strained *N*-Boc azetidine (**18**). Notably, we did not attempt the reaction with phenolic derivatives or *tert*-butanol considering their lack of reactivity in the racemic reaction.^17^ Turning to the dioxazolone scope, the reaction tolerated aryl dioxazolones, albeit in reduced yield (**19**). We observed that increasing steric bulk on the α-carbon of the dioxazolone led to diminished yield and stereoselectivity, with *t*-butyl dioxazolone providing virtually no diastereoselectivity (**20–22**). We attribute this to a reduced rate of nitrene formation and insertion due to steric hindrance, allowing for scrambling of the *anti*-stereochemistry (*vide infra*). We note that additional alkene functionality in product **23** was preserved. Finally, an alpha stereocenter on the dioxazolone was maintained for both enantiomers (**24a**–**b**), with little to no effect on the chiral control provided by the catalyst.

**Figure 4:**
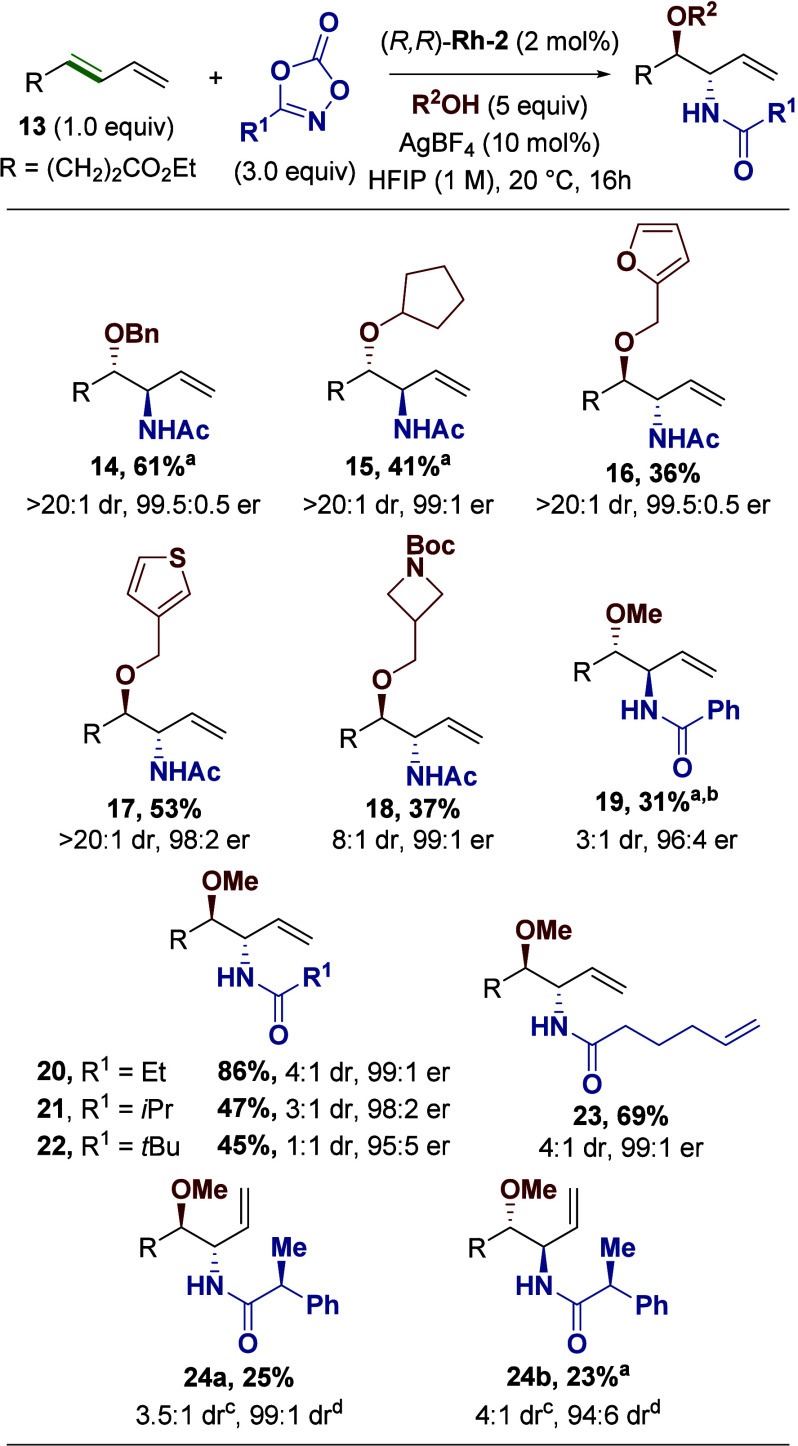
Scope of nucleophiles and dioxazolones. Reactions were run on a 0.25 mmol scale. Diastereomeric ratios were determined from crude NMR. Enantiomeric ratios were determined by chiral HPLC. ^a^Reaction was run with (*S*,*S*) catalyst. ^b^Reaction was run with 4 mol % catalyst and 20 mol % AgBF_4_. ^c^dr between newly formed stereocenters. ^d^dr between the dioxazolone stereocenter and new stereocenters.

To account for the observed stereochemical outcome of the reaction, and incorporating the previous observations by Rovis and co-workers,^16,17^ we propose the following mechanism (Figure 5A). Although the diene is likely to bind predominantly in an η^4^ mode after formation of the cationic Rh complex, with the R substituent oriented away from the phenyl group on the catalyst to minimize steric interactions (**I-A**), subsequent nucleophilic attack on this complex would proceed through a high-energy transition state and lead to intermediate **II-A** in which the π-allyl ligand experiences considerable 1,3-allylic strain. We propose that there is a small equilibrium population of intermediate **I-B**, in which only the terminal alkene is bound to Rh and the diene is arranged in an *s*-trans configuration. The Rh-coordination inductively activates the internal olefin for nucleophilic attack, in this case leading to the lower energy π-allyl complex **II-B** and diminishing the 1,3-allylic strain compared to **II-A**. Subsequent nitrenoid formation (**III**) and insertion (**IV**) occurs in an *anti* fashion, setting the second stereocenter and contributing to the diastereoselectivity of the reaction. Protodemetalation of complex **IV** regenerates the catalyst and furnishes the desired oxyamination product.

**Figure 5:**
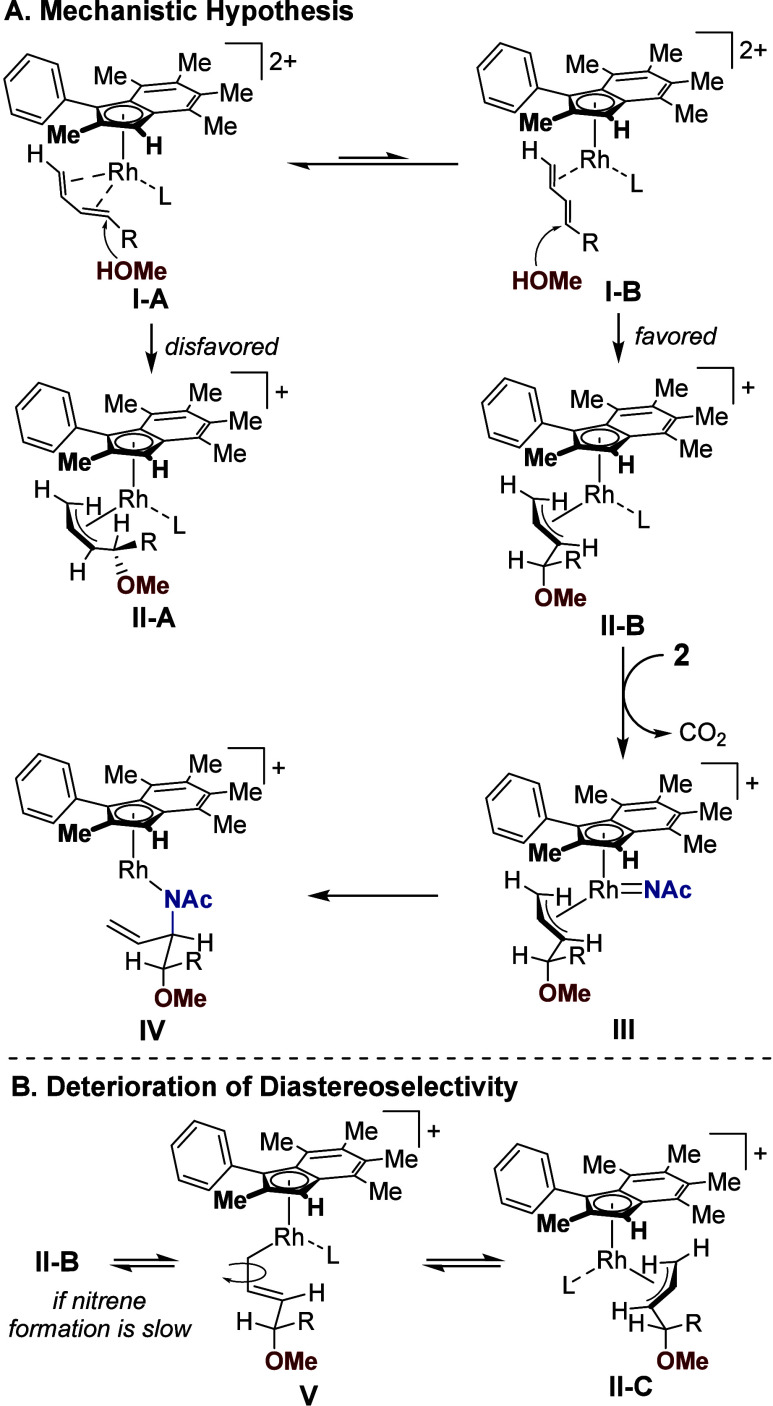
Mechanistic hypothesis explaining the observed stereochemistry. Mechanism is depicted using (*S*,*S*)-**Rh-2**. L = solvent or dioxazolone.

This mechanistic proposal also explains the poor diastereoselectivity observed both when using dioxazolone as the limiting reagent (Figure 2, entry 4) and when using sterically encumbered dioxazolones (Figure 5B). As identified by Rovis,^16^ a shift in coordination of complex **II-B** from the η^3^ to η^1^ mode can form complex **V**, allowing for free bond rotation. Reformation of the η^3^ complex **II-C** leads to the *syn* diastereomer, diminishing diastereoselectivity. A decreased concentration of dioxazolone or use of a dioxazolone with a large α substituent slows the nitrene addition into the π-allyl species **III**, allowing more time for the deleterious side pathway to occur.

In conclusion, we have demonstrated a highly enantioselective three-component 3,4-oxyamination of 1,3-dienes catalyzed by an electron-rich planar chiral Rh­(III) indenyl catalyst. This process has proven useful for rapid modular synthesis of chiral vicinal amino alcohols on the mmol scale, and its mild conditions offer access to a broad range of coupling partners. Extension of this reactivity into other nucleophiles and alkene functionalizations is currently ongoing in our laboratory.

## Supplementary Material



## Data Availability

The data underlying this study are available in the published article and its Supporting Information.

## Notes

Experimental procedures, further optimization tables, characterization data, spectra, and crystallographic data (PDF)
