# Supplementary material for: Enantioselective 3,4-Oxyamination of 1,3-Dienes Enabled by an Electron-Rich Planar Chiral Rhodium Indenyl Catalyst
Source: Org Lett. 2025 Jun 30;27(27):7412–6. doi: 10.1021/acs.orglett.5c02176 (PMC12261335; doi:10.1021/acs.orglett.5c02176)
Supplement: Supplementary file 1 [file ol5c02176_si_001.pdf]

Supporting Information For

Enantioselective 3,4-Oxyamination of 1,3-Dienes Enabled by an Electron-Rich Planar Chiral Rhodium Indenyl Catalyst

Ethan M. P. Heyboer, Wesley A. Pullara, John Bacsa, Simon B. Blakey*

Department of Chemistry, Emory University, Atlanta, Georgia 30322, United States

*email: sblakey@emory.edu

General Procedures **S2**
Catalyst Synthesis **S3**
Preparation of Starting Materials **S4**
Optimization Studies **S6**
Nona-1,3-Diene Decomposition Study **S7**
Stereochemical Assignment **S9**
General Procedure for Enantioselective 3,4-Oxyamination of 1,3-Dienes **S10**
NMR Spectra and HPLC Traces for New Compounds **S18**
X-Ray Crystallographic Data **S62**
(*S*,*S*)-**Rh-2** (CCDC 2434467) **S62**
10 (CCDC 2454911) **S78**
References **S87**

1. General Procedures

All reactions were conducted under nitrogen atmosphere with anhydrous solvents in oven- or flame-dried glassware using standard Schlenk technique, unless otherwise stated. Anhydrous dichloromethane (DCM), diethyl ether (Et_2O), tetrahydrofuran (THF), and were obtained by passage through activated alumina using a *Glass Contours* solvent purification system. 1,1,1,3,3,3-hexaflouroisopropanol (HFIP) was distilled over activated 4Å molecular sieves and stored over activated 4Å molecular sieves. Solvents for workup, extraction, and column chromatography were used as received from commercial suppliers without further purification. All catalysts were stored and weighed in a nitrogen-filled glovebox. All other chemicals were purchased from Millipore Sigma, Strem Chemicals, Oakwood Chemicals, Alfa Aesar, TCI, Combi Blocks, or Ambeed and used as received without further purification, unless otherwise stated.

^{1}H and ^{13}C nuclear magnetic resonance (NMR) spectra were recorded on a Varian Inova 600 spectrometer (600 MHz ^{1}H, 151 MHz ^{13}C), a Varian Inova 500 spectrometer (500 MHz ^{1}H, 126 MHz ^{13}C), a Bruker 400 spectrometer (400 MHz ^{1}H, 126 MHz ^{13}C), and a Varian Inova 400 spectrometer (400 MHz ^{1}H, 126 MHz ^{13}C). Chemical shifts δ values were reported in parts per million (ppm) relative to $CHCl_3$ (7.26 ppm for ^{1}H, 77.16 ppm for ^{13}C) for $CDCl_3$, relative to C_6H_6 ((7.16 ppm for ^{1}H, 128.06 ppm for ^{13}C) for C_6D_6, relative to DMSO (2.50 ppm for ^{1}H, 39.52 ppm for ^{13}C) for DMSO-d_6. Coupling constants (J values) were reported in Hz and multiplicities were indicated using the following abbreviations: s = singlet, d = doublet, t = triplet, q = quartet, qn = quintet, m = multiplet, br = broad. High resolution mass spectra (HRMS) were obtained using a Thermo Electron Corporation Finigan LTQFTMS (at the Mass Spectrometry Facility, Emory University). High Pressure Liquid Chromatography (HPLC) was performed on an Agilent 1260 Infinity II series HPLC utilizing CHIRALPAK® IA, IB, IH, IJ, and IK 4.6 x 150 mm analytical columns. Semi preparative HPLC was performed on an Agilent 1260 Infinity II series preparative HPLC using a CHIRALCEL® OD-H 20 x 250 mm column. Optical rotations were measured on a PerkinElmer 341 polarimeter. Analytical thin layer chromatography (TLC) was performed on precoated glass-backed Silicycle SiliaPureR 0.25 mm silica gel 60 plates and visualized with UV light or potassium permanganate ($KMnO_4$) stain. Silica gel column chromatography was performed using Silicycle SiliaFlashR F60 silica gel (40-63 μm). Flash column chromatography was performed using Silicycle SiliaFlashR F60 silica gel (40-63 μm) on a Biotage Isolera One system. Preparatory TLC was performed on precoated glass backed Silicycle SiliaPureR 1.0 mm silica gel 60 plates.

2. Catalyst Synthesis

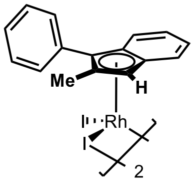

Rh-1

(±)-**Rh-1** catalyst was synthesized following the literature procedure. Spectroscopic data match those previously known in the literature.[1]

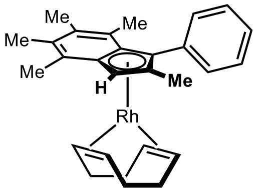

(*R*)-**Rh-S1**

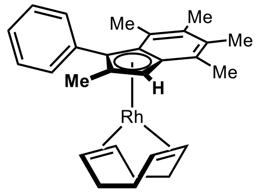

(*S*)-**Rh-S1**

(*R*)-**Rh-S1** and (*S*)-**Rh-S1** were synthesized and resolved via chiral HPLC following a known literature procedure.[2]

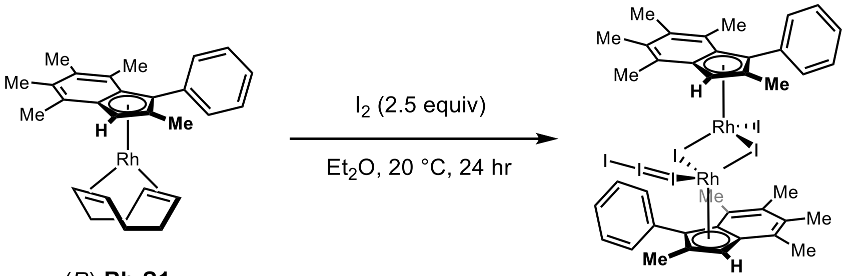

(*R*)-**Rh-S1**

(*R,R*)-**Rh-2**

(R,R)-(η³-2,4,5,6,7-pentamethyl-3-phenylinden-1H-yl) rhodium(III) triiodide dimer ((R,R)-**Rh-2**): With no precautions against air or moisture exposure, I_2 crystals (110 mg, 0.43 mmol, 2.5 equiv.) were added to a 25 mL round bottom flask equipped with a stir bar and containing a solution of resolved (*R*)-**Rh-S1** (82 mg, 0.17 mmol, 1.0 equiv.) in Et_2O (0.03M). The vial was capped to prevent solvent evaporation and the solution was stirred for 24 hours. The reaction was filtered through a Buchner funnel washing with excess Et_2O until the filtrate was clear. The fine black powder was collected from the filter paper, then subsequently purified using flash column chromatography (0% - 5% MeOH in DCM) to give the (*R,R*)-**Rh-2** precatalyst as a powdery black material (78 mg, 0.052 mmol, 60% yield), which was stored in a nitrogen filled glovebox. **^{1}H NMR** (600 MHz, DMSO-d_6) δ 8.26-8.24 (m, 2H), 7.47-7.42 (m, 4H), 7.39-7.36 (m, 2H), 7.28-7.26 (m, 2H), 6.44 (s, 2H), 2.46 (s, 6H), 2.18 (s, 6H), 2.11 (s, 6H), 2.02 (s, 6H), 1.87 (s, 6H). **^{13}C NMR** (151 MHz, $CDCl_3$) δ 142.7, 141.6, 133.5, 131.0, 130.7, 129.9, 129.2, 128.5, 128.1, 127.7, 112.5 (d, *J* = 6.0 Hz), 107.2 (d, *J* = 2.9 Hz), 104.9 (d, *J* = 3.7 Hz), 95.9 (d, *J* = 6.9 Hz), 75.1 (d, *J* = 7.0 Hz), 18.3, 17.8, 17.5, 17.2, 14.0. **mp** = 206-212 °C. **HRMS:** (+APCI) calculated for $C_{40}H_{42}{}^{127}I_3{}^{103}Rh_2$ $[M-I_3]^+$ 1108.8525, found 1108.8525.

(*S,S*)-**Rh-2** and (±)-**Rh-2** were prepared using the same method. Purple-black crystals of (*S,S*)-**Rh-2** suitable for X-ray crystallography were obtained via evaporation from chloroform.

To confirm our structural assignment, we repeated the oxidation under the reaction conditions described several times and obtained crystal structures of (*S,S*)-**Rh-2** on two separate occasions. Furthermore, both the ^{1}H and ^{13}C data are indicative of a single product, suggesting that the structure assigned to (*S,S*)-**Rh-2** is correct and that it is unlikely to have arisen from anomalous crystal picking.

3. Preparation of Starting Materials

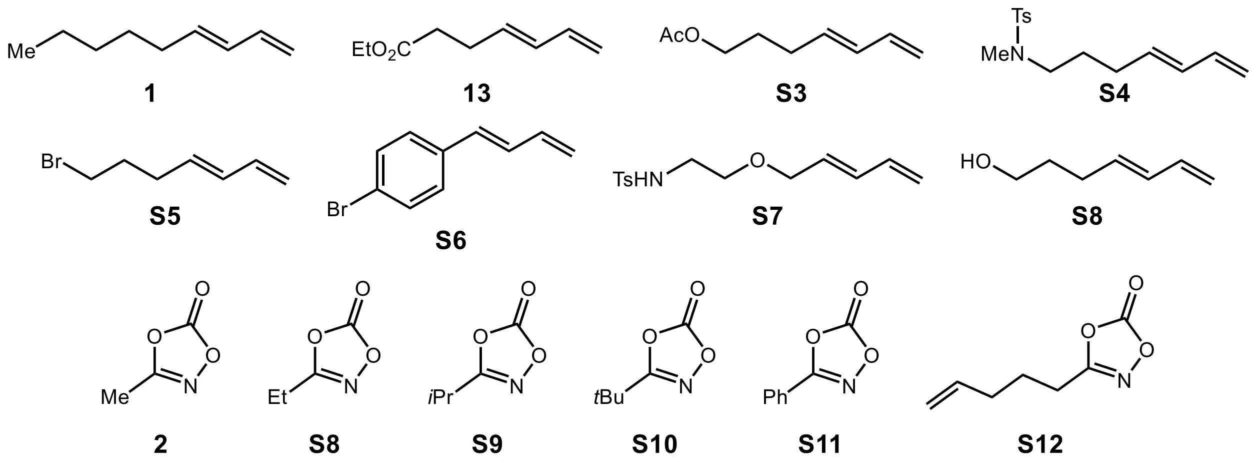

The 1,3-dienes **1**,[3] **12**,[3] **S3**,[3] **S4**,[3] **S5**,[3] **S6**,[3] **S7**,[3] and **S8**[4] were synthesized following known literature procedures. 1,3-pentadiene and buta-1,3-dien-1-ylbenzene were purchased from TCI and Sigma Aldrich, respectively. The dioxazolones **2**,[1] **S8**,[5] **S9**,[6] **S10**,[1] **S11**,[1] and **S12**[3] were synthesized following known literature procedures.

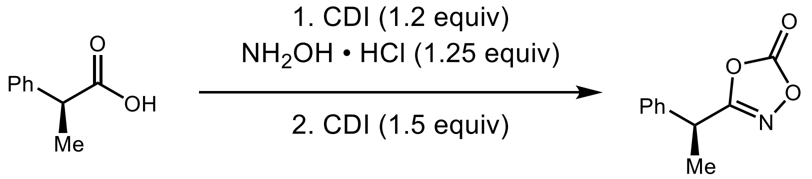

S13

(S)-3-(1-phenylethyl)-1,4,2-dioxazol-5-one (**S13**): Prepared in an analogous fashion to (±)-3-(1-phenylethyl)-1,4,2-dioxazol-5-one.[7] Inside a glovebox, an oven-dried 50 mL round bottom flask equipped with a stir bar was charged with 1,1'-carbonyldiimidazole (CDI, 2.0 g, 12.6 mmol, 1.2 equiv), then sealed with a rubber septum and removed from the glovebox. Acetonitrile (20 mL, 0.5M) was added, followed by *(S)*-2-phenylpropionic acid (1.43 mL, 10.5 mmol, 1.0 equiv). Immediate evolution of CO_2 was observed, and the reaction was allowed to stir for 3 hours. The flask was then opened and NH_2OH • HCl (912 mg, 13.1 mmol, 1.25 equiv) was quickly added. The flask was resealed and allowed to stir under an N_2 balloon for 16 hours. To the resulting solution was added another portion of CDI (2.6 g, 15.8 mmol, 1.5 equiv), and the reaction was allowed to stir for another 2 hours. The reaction was quenched with 1M aq. HCl, then extracted with DCM (3x 30 mL). The combined organic layers were washed with brine (3x 50 mL) and dried over $MgSO_4$, then concentrated under reduced pressure. The resulting crude dioxazolone was dissolved in minimal DCM, then filtered through a pad of silica gel, washing with DCM, to afford the pure dioxazolone as a clear, colorless liquid (283 mg, 1.48 mmol, 14% yield, 98:2 er). Spectroscopic data match those reported in the literature for the racemic product.[7] **HPLC** (IH, 2-propanol/*n*-hexane = 20/80, flow rate = 1.0 mL/min, l = 210 nm) t_M = 7.5 min, t_m = 6.1 min, 98:2 e

(±)-3-(1-phenylethyl)-1,4,2-dioxazol-5-one (**S14**): Synthesized following known literature procedure.[7]

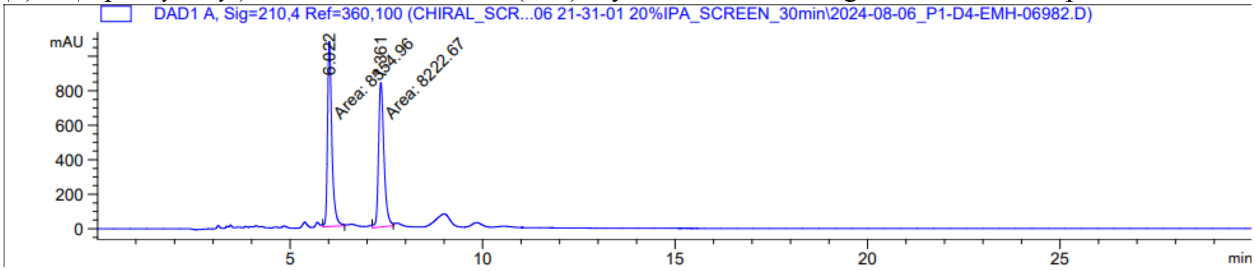

Peak #	RetTime [min]	Type	Width [min]	Area [mAU*s]	Height [mAU]	Area %
1	6.022	MM	0.1300	8354.95801	1071.14685	50.3990
2	7.361	MM	0.1636	8222.67383	837.91077	49.6010

(S)-3-(1-phenylethyl)-1,4,2-dioxazol-5-one (**S13**):

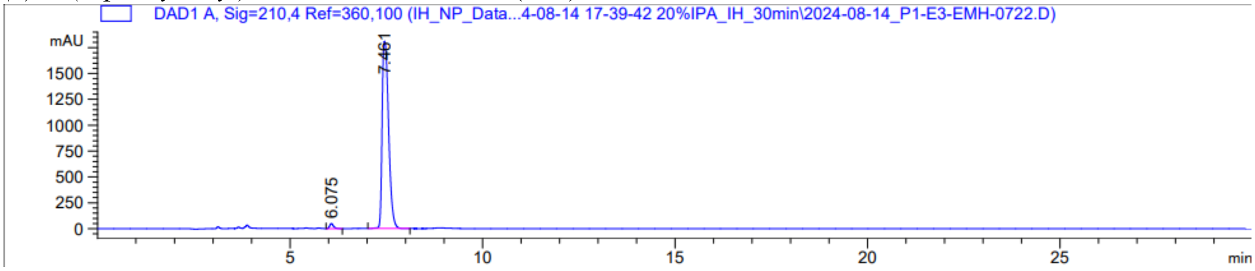

Peak #	RetTime [min]	Type	Width [min]	Area [mAU*s]	Height [mAU]	Area %
1	6.075	BV R	0.0948	333.75519	49.74102	1.5620
2	7.461	VV R	0.1392	2.10327e4	1808.11414	98.4380

4. Optimization Studies

All reactions were run under the following conditions: An oven-dried 4 mL vial equipped with stir bar was brought into the glovebox, where it was charged with (±)-**Rh-2** (7.5 mg, 5 µmol, 2 mol%) and silver salt, capped with septum, and removed from the glovebox. A separate oven-dried 4 mL vial was charged with nona-1,3-diene (31 mg, 250 µmol, 1.0 equiv), 3-methyl-1,4,2-dioxazol-5-one (76 mg, 750 µmol, 3.0 equiv), and MeOH (50 µL, 1.25 mmol, 5.0 equiv). Two washes of solvent were used to transfer the diene solution into the reaction vial through the septum, such that the final concentration of the reaction was 1.0 M (i.e. the total combined volume of solvent and MeOH = 0.250 mL). The vial was sealed with parafilm, sonicated for 15 seconds, then stirred at 21 °C for 16 hours. After 16 hours, the reaction was diluted with ethyl acetate, filtered over Celite, and the resulting material was concentrated *in vacuo*. Crude reaction mixtures were analyzed via ^{1}H NMR. All yields and dr values were calculated using mesitylene as internal standard.

Figure S1. Solvent screen.

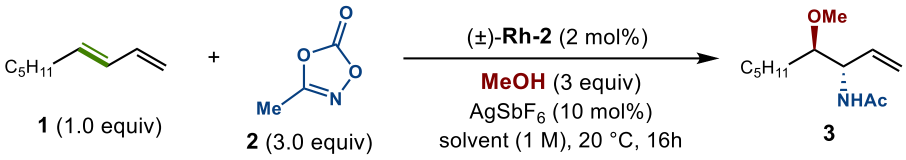

Entry	Solvent	Results
1	Acetone	12%, -- dr
2	THF	7%, -- dr
3	MeOH	9%, -- dr
4	TFE	10% -- dr
5	HFIP	40%, >20:1 dr

Figure S2. Silver additive screen.

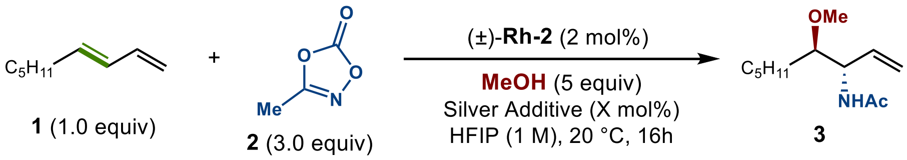

Entry	Silver Additive	X	Results
1	$AgNTf_2$	10%	21%, >20:1 dr
2	AgOTf	10%	29%, >20:1 dr
3	$AgPF_6$	10%	50%, >20:1 dr
4	$AgSbF_6$	10%	50%, >20:1 dr
5	$AgBF_4$	10%	62%, >20:1 dr
6	$AgBF_4$	15%	23%, >20:1 dr
7	$AgBF_4$	20%	25%, >20:1 dr

5. Nona-1,3-Diene Decomposition Study

To assess the proposed decomposition of nona-1,3-diene under reaction conditions, an oven-dried 4 mL vial equipped with stir bar was charged with nona-1,3-diene (31 mg, 250 µmol), 3-methyl-1,4,2-dioxazol-5-one (76 mg, 750 µmol), and mesitylene (35 µL, 250 µmol), then 250 µL of HFIP were added and the mixture was allowed to stir at room temperature. At $t = 0$, 1, 2, and 8 hours, aliquots were removed for ^{1}H NMR analysis. The yield of remaining diene was determined using mesitylene as internal standard.

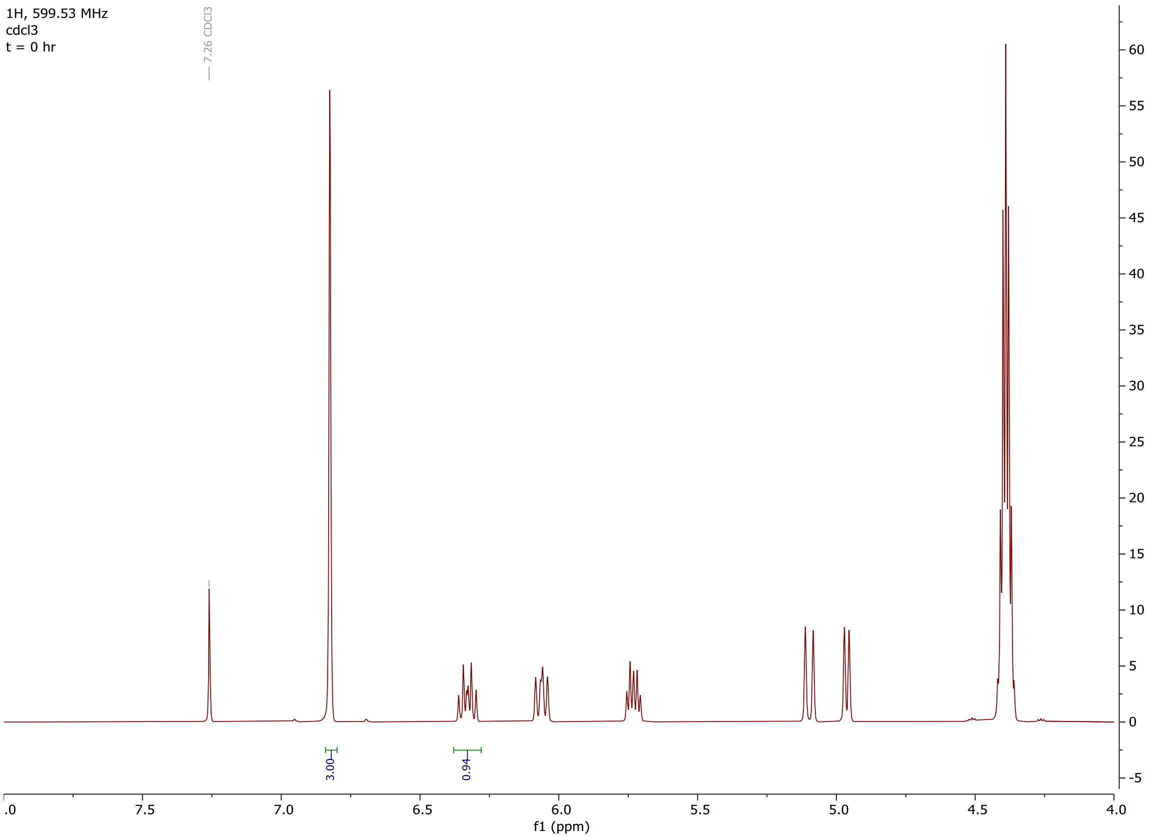

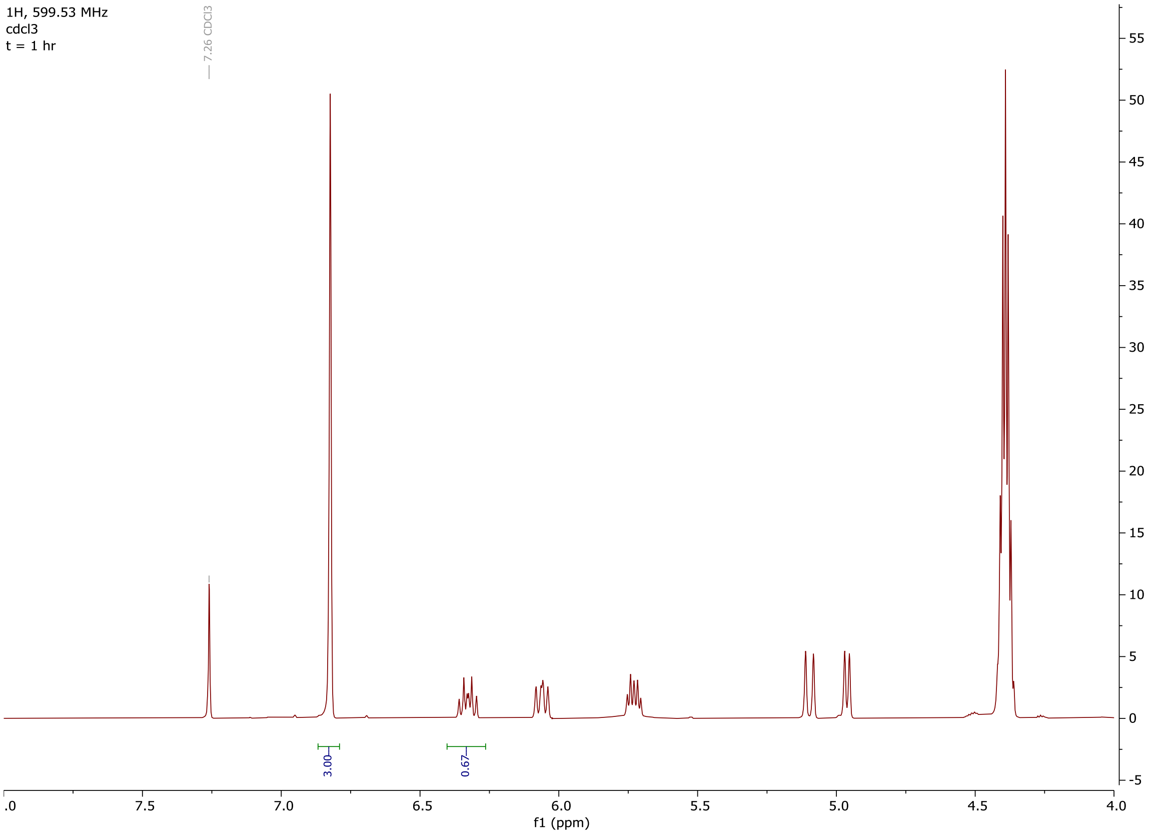
1H, 599.53 MHz
cdcl3
t = 1 hr
7.26 CDCl3
3.00
0.67
f1 (ppm)

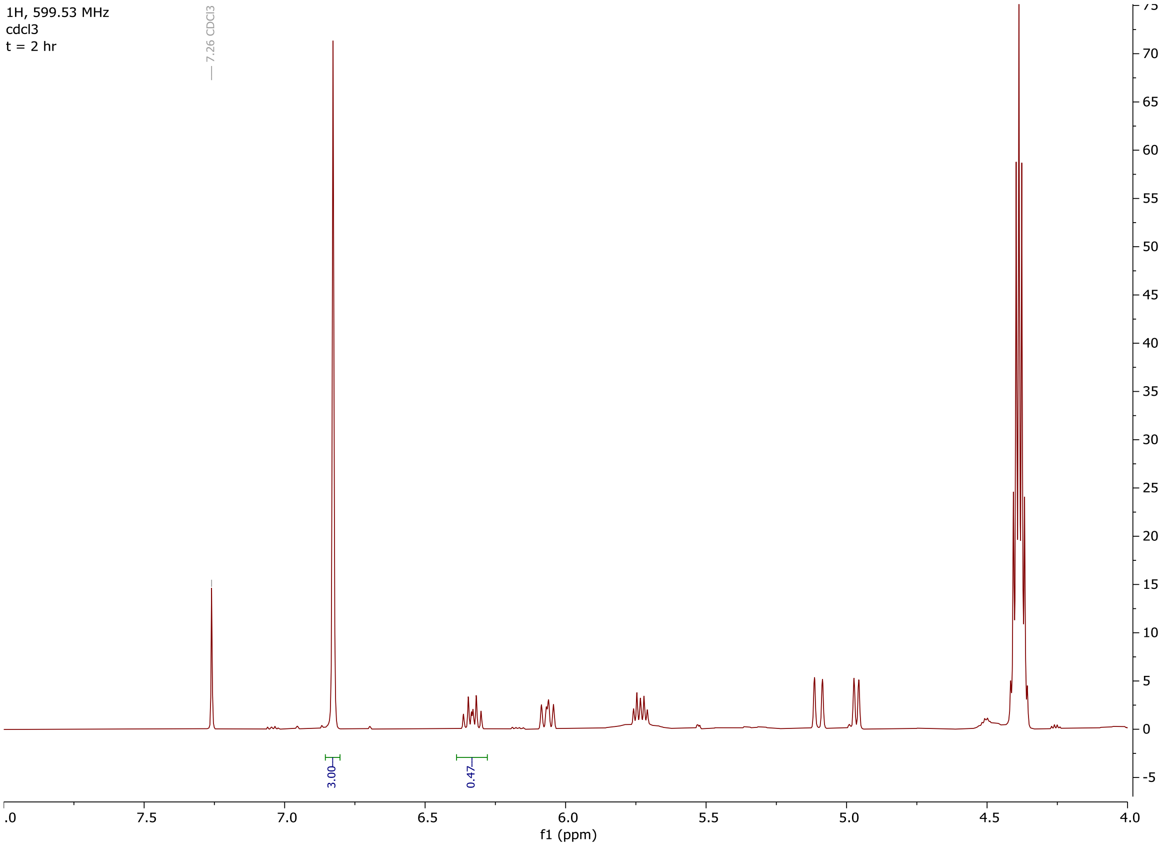
1H, 599.53 MHz
cdcl3
t = 2 hr
7.26 CDCl3
3.00
0.47
f1 (ppm)

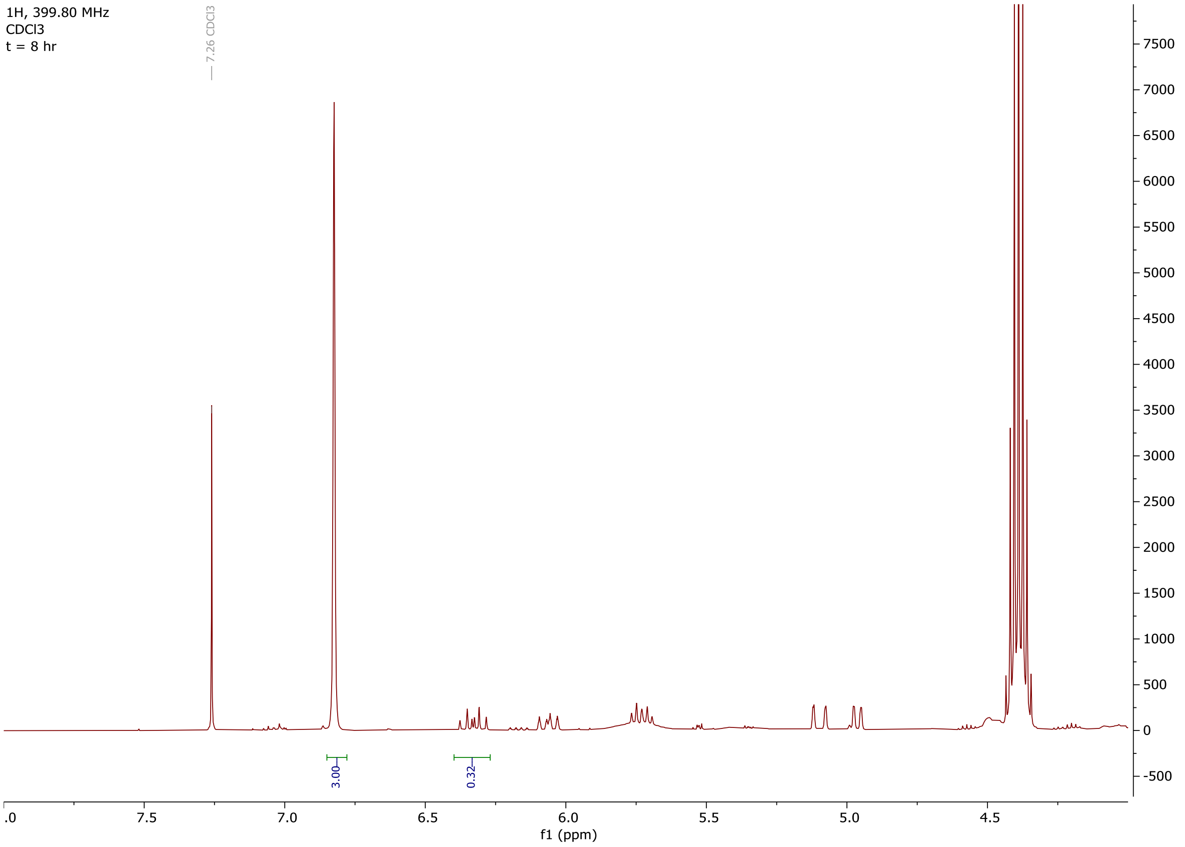

6. Stereochemical Assignment

A crystal structure (Figure S3) of **10** prepared using (*S,S*)-**Rh-2** was obtained in order to assign absolute stereochemistry of the oxyamination product. Stereochemistry of all other products were assigned by analogy.

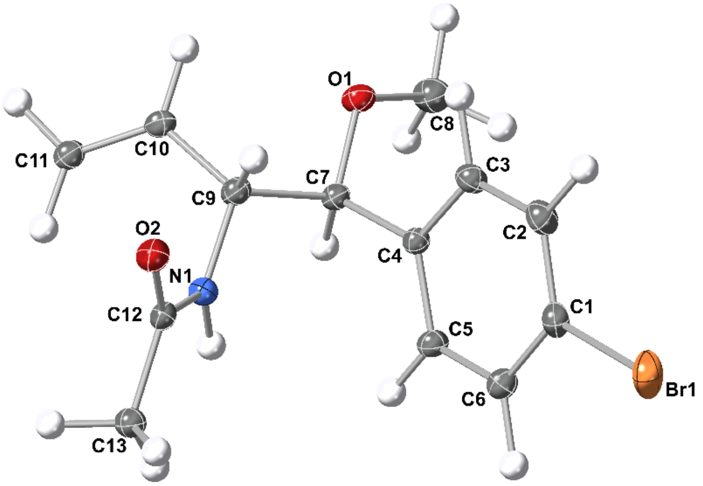

Figure S3. The crystal structure of **10**.

7. General Procedure for Enantioselective 3,4-Oxyamination of 1,3-Dienes

An oven-dried 4 mL vial equipped with stir bar was brought into the glovebox, where it was charged with (*R,R*)-**Rh-2** (7.5 mg, 5 μmol, 2 mol%) and $AgBF_4$ (4.9 mg, 25 μmol, 10 mol%), capped with septum, and removed from the glovebox. A separate oven-dried 4 mL vial was charged with diene (250 μmol, 1.0 equiv.), dioxazolone (750 μmol, 3.0 equiv.), and nucleophile (1.25 mmol, 5.0 equiv.). Two washes of HFIP were used to transfer the diene solution into the reaction vial through the septum, such that the final concentration of the reaction was 1.0 M (i.e. the total combined volume of HFIP and nucleophile = 0.250 mL). The vial was sealed with parafilm, sonicated for 15 seconds, then stirred at 21 °C for 16 hours. After 16 hours, the reaction was diluted with ethyl acetate, filtered over Celite, and the resulting material was concentrated *in vacuo.* The crude reaction mixture was purified via FCC to afford purified products **3-24.** Some products required further purification via preparatory TLC.

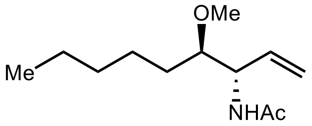

N-((3S,4R)-4-methoxynon-1-en-3-yl)acetamide (**3**). Prepared using the General Procedure and purified with FCC (ethyl acetate/hexanes = 40/60) to provide a colorless oil (32.0 mg, 60% yield, 97:3 er). **^{1}H NMR** (600 MHz, $CDCl_3$) δ 5.88 (d, *J* = 8.9 Hz, 1H), 5.77 (ddd, *J* = 17.1, 10.4, 6.7 Hz, 1H), 5.24-5.20 (m, 2H), 4.61 (m, 1H), 3.39 (s, 3H), 3.24 (td, *J* = 6.3, 3.1 Hz, 1H), 2.02 (s, 3H), 1.58-1.53 (m, 1H), 1.40-1.25 (m, 7H), 0.88 (t, *J* = 6.9 Hz, 3H). Spectral data match those reported in the literature.[3] **HPLC** (IH, 2-propanol/*n*-hexane = 20/80, flow rate = 1.0 mL/min, l = 210 nm) t_R = 4.9 min (major), 11.3 min (minor). **$[\alpha]_D^{20}$ =** -24.13 (*c* = 0.30, $CHCl_3$).

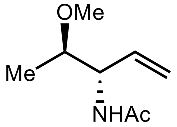

N-((3S,4R)-4-methoxypent-1-en-3-yl)acetamide (**4**). Prepared using the General Procedure and purified with FCC (acetone/hexanes = 30/70) on a 1.0 mmol scale to provide a yellow oil (74.5 mg, 47% yield, 96:4 er) **^{1}H NMR** (400 MHz, $CDCl_3$) δ 5.95 (br s, 1H), 5.78 (ddd, *J* = 17.5, 10.1, 6.8 Hz, 1H), 5.24 (dt, *J* = 4.7, 1.4 Hz, 1H), 5.21 (qn, *J* = 1.5 Hz, 1H), 4.47 (dddt, *J* = 8.2, 6.6, 3.0, 1.3 Hz, 1H), 3.46 (qd, *J* = 6.4, 3.2 Hz, 1H), 3.34 (s, 3H), 2.02 (s, 3H), 1.13 (d, *J* = 6.3 Hz, 3H). Spectral data match those reported in the literature.[3] **HPLC** (IH, 2-propanol/*n*-hexane = 20/80, flow rate = 1.0 mL/min, l = 210 nm) t_R = 8.8 min (major), 11.6 min (minor). **$[\alpha]_D^{20}$ =** -6.32 (*c* = 0.82, $CHCl_3$).

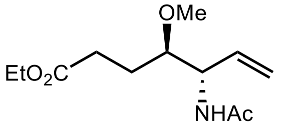

Ethyl (4R,5S)-5-acetamido-4-methoxyhept-6-enoate (**5**). Prepared using the General Procedure and purified with FCC (acetone/hexanes = 30/70) to provide a colorless oil (45.8 mg, 75% yield, 98:2 er). **^{1}H NMR** (400 MHz, $CDCl_3$) δ 5.81-5.73 (m, 2H), 5.25 (dt, *J* = 10.1, 1.4 Hz, 1H), 5.22 (dt, *J* = 3.0, 1.3 Hz, 1H), 4.68-4.62 (m, 1H), 4.13 (q, *J* = 7.1 Hz, 2H), 3.40 (s, 3H), 3.29 (ddd, *J* = 7.5, 5.9, 3.7 Hz, 1H), 2.39 (ddd, *J* = 8.0, 6.9, 3.5 Hz, 2H), 2.03 (s, 3H), 1.88-1.79 (m, 1H), 1.77-1.69 (m, 1H), 1.26 (t, *J* = 7.1 Hz, 3H). Spectral data match those reported in the literature.[3] **HPLC** (IH, 2-propanol/*n*-hexane = 20/80, flow rate = 1.0 mL/min, l = 210 nm) t_R = 6.9 min (minor), 14.0 min (major). **$[\alpha]_D^{20}$ =** +19.77 (*c* = 1.06, $CHCl_3$).

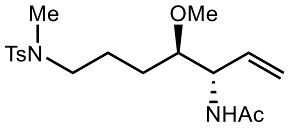

N-((3S,4R)-7-((N,4-dimethylphenyl)sulfonamido)-4-methoxyhept-1-en-3-yl)acetamide (**6**). Prepared using the General Procedure and purified with FCC (acetone/hexanes = 40/60) and preparatory TLC (100% ethyl acetate) to provide a yellow oil (35.0 mg, 38% yield, 99:1 er). **^{1}H NMR** (400 MHz, $CDCl_3$) δ 7.65 (d, *J* = 8.3, 2H), 7.32 (d, *J* = 7.8 Hz, 2H), 5.86 (d, *J* = 8.8 Hz, 1H), 5.78 (ddd, *J* = 17.1, 10.5, 6.6 Hz, 1H), 5.23 (dt, *J* = 8.1, 1.4 Hz, 1H), 5.20 (d, *J* = 1.5 Hz, 1H), 4.63-4.58 (m, 1H), 3.39 (s, 3H), 3.28 (td, *J* = 6.3, 3.8 Hz, 1H), 3.01-2.97 (m, 2H), 2.68 (s, 3H), 2.42 (s, 3H), 2.03 (s, 3H), 1.64-1.45 (m, 4H). Spectral data match those reported in the literature.[3] **HPLC** (IH, 2-propanol/*n*-hexane = 40/60, flow rate = 1.0 mL/min, l = 210 nm) t_R = 8.5 min (major), 17.5 min (minor). **$[\alpha]_D^{20}$ =** -11.52 (*c* = 1.20, $CHCl_3$).

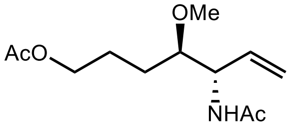

(4R,5S)-5-acetamido-4-methoxyhept-6-en-1-yl acetate (**7**). Prepared using the General Procedure and purified with FCC (ethyl acetate/hexanes = 70/30) to provide a colorless oil (27.3 mg, 45% yield, 99:1 er). **^{1}H NMR** (400 MHz, $CDCl_3$) δ 5.84-5.72 (m, 2H), 5.25 (dt, *J* = 8.9, 1.4 Hz, 1H), 5.22 (m, 1H), 4.62 (ddq, *J* = 8.6, 5.0, 1.7 Hz, 1H), 4.06 (t, *J* = 6.5 Hz, 2H), 3.40 (s, 3H), 3.27 (td, *J* = 6.6, 3.4 Hz, 1H), 2.05 (s, 3H), 2.03 (s, 3H), 1.77-1.64 (m, 2H), 1.63-1.54 (m, 1H), 1.49-1.40 (m, 1H). Spectral data match those reported in the literature.[3] **HPLC** (IH, 2-propanol/*n*-hexane = 30/70, flow rate = 1.0 mL/min, l = 210 nm) t_R = 5.7 min (major), 10.5 min (minor). **$[\alpha]_D^{20}$ =** -13.09 (*c* = 0.79, $CHCl_3$).

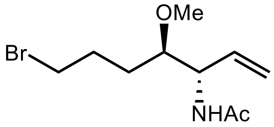

N-((3S,4R)-7-bromo-4-methoxyhept-1-en-3-yl)acetamide (**8**). Prepared using the General Procedure and purified with FCC (acetone/pentane = 30/70) to provide an orange oil (25.1 mg, 38% yield, 98:2 er). **^{1}H NMR** (400 MHz, $CDCl_3$) δ 5.82-5.74 (m, 2H), 5.26 (dt, *J* = 8.3, 1.4 Hz, 1H), 5.23 (d, *J* = 1.4 Hz, 1H), 4.62 (m, 1H), 3.45-3.41 (m, 5H), 3.28 (td, *J* = 6.7, 3.5 Hz, 1H), 2.03 (s, 3H), 1.99-1.86 (m, 2H), 1.73-1.64 (m, 1H), 1.61-1.52 (m, 1H). Spectral data match those reported in the literature.[3] **HPLC** (IK, 2-propanol/*n*-hexane = 10/90, flow rate = 1.0 mL/min, l = 210 nm) t_R = 9.2 min (minor), 10.3 min (major). **$[\alpha]_D^{20}$ =** -20.00 (*c* = 0.31, $CHCl_3$).

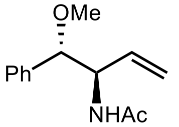

N-((1S,2R)-1-methoxy-1-phenylbut-3-en-2-yl)acetamide (**9**). Prepared using the General Procedure with (*S,S*)-**Rh-2** and purified with FCC (acetone/hexanes = 20/80) to provide a yellow oil (23.6 mg, 43% yield, 91:9 er). **^{1}H NMR** (400 MHz, $CDCl_3$) δ 7.38-7.34 (m, 2H), 7.31-7.27 (m, 3H), 5.98 (d, *J* = 9.1 Hz, 1H), 5.72 (ddd, *J* = 17.2, 10.5, 5.8 Hz, 1H), 5.09 (dt, *J* = 10.5, 1.4 Hz, 1H), 5.00 (dt, *J* = 17.2, 1.5 Hz, 1H), 4.70 (dddt, *J* = 9.1, 5.2, 3.3, 1.6 Hz, 1H), 4.41 (d, *J* = 3.5 Hz, 1H), 3.33 (s, 3H), 2.05 (s, 3H). Spectral data match those reported in the literature.[3] **HPLC** (IA, 2-propanol/*n*-hexane = 5/95, flow rate = 1.0 mL/min, l = 210 nm) t_R = 10.4 min (minor), 11.5 min (major). **$[\alpha]_D^{20}$ =** -70.06 (*c* = 0.31, $CHCl_3$).

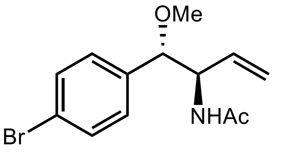

N-((1S,2R)-1-(4-bromophenyl)-1-methoxybut-3-en-2-yl)acetamide (**10**). Prepared using the General Procedure with (*S,S*)-**Rh-2** and purified with FCC (acetone/pentane = 20/80) and preparatory TLC (100% ethyl acetate) to provide a colorless solid (14.1 mg, 19% yield, 88:12 er). Crystals suitable for X-ray crystallography were obtained via slow evaporation from chloroform and heptane. **^{1}H NMR** (400 MHz, $CDCl_3$) δ 7.48 (dt, *J* = 8.3, 1.5 Hz, 2H), 7.16 (dt, *J* = 8.4, 1.8 Hz, 2H), 5.97 (d, *J* = 9.1 Hz, 1H), 5.69 (ddd, *J* = 16.9, 10.5, 6.2 Hz, 1H), 5.09 (dt, *J* = 10.6, 1.4 Hz, 1H), 4.98 (dt, *J* = 17.2, 1.5 Hz, 1H), 4.63 (dddt, *J* = 9.1, 6.1, 3.0, 1.4 Hz, 1H), 4.36 (d, *J* = 3.5 Hz, 1H), 3.30 (s, 3H), 2.04 (s, 3H). Spectral data match those reported in the literature.[3] **mp** = 108-112 °C. **HPLC** (IB, 2-propanol/*n*-hexane = 10/90, flow rate = 1.0 mL/min, l = 210 nm) t_R = 6.5 min (minor), 7.3 min (major). **$[\alpha]_D^{20}$ =** +29.57 (*c* = 0.23, $CHCl_3$).

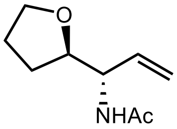

N-((S)-1-((R)-tetrahydrofuran-2-yl)allyl)acetamide (**11**). Prepared using the General Procedure, omitting the nucleophile, and purified with FCC (acetone/hexanes = 20/80) to provide a colorless oil (29.3 mg, 69% yield, 82:18 er). **^{1}H NMR** (400 MHz, $CDCl_3$) δ 5.98 (br s, 1H), 5.78 (ddd, *J* = 17.4, 10.3, 7.2 Hz, 1H), 5.27-5.20 (m, 2H), 4.50-4.44 (m, 1H), 3.96 (td, *J* = 7.2, 3.9 Hz, 1H), 3.81 (dt, *J* = 8.2, 6.5 Hz, 1H), 3.72 (dt, *J* = 8.2, 6.7 Hz, 1H), 1.98 (s, 3H), 1.96-1.82 (m, 3H), 1.71-1.63 (m, 1H). Spectral data match those reported in the literature.[3] **HPLC** (IH, 2-propanol/*n*-hexane = 15/85, flow rate = 1.0 mL/min, l = 210 nm) t_R = 17.6 min (minor), 19.2 min (major). **$[\alpha]_D^{20}$ =** -26.70 (*c* = 1.09, $CHCl_3$).

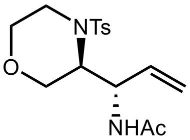

N-((S)-1-((S)-4-tosylmorpholin-3-yl)allyl)acetamide (**12**). Prepared using the General Procedure, omitting the nucleophile, and purified with FCC (acetone/pentane = 40/60) to provide a yellow oil (46.0 mg, 54% yield, 95:5 er). **^{1}H NMR** (400 MHz, $CDCl_3$) δ 7.69 (dt, *J* = 8.3, 1.7 Hz, 2H), 7.33 – 7.30 (m, 2H), 6.09 (d, *J* = 8.9 Hz, 1H), 6.00 (ddd, *J* = 17.2, 10.4, 5.7 Hz, 1H), 5.25 (dt, *J* = 17.1, 1.4 Hz, 1H), 5.18 (dt, *J* = 10.4, 1.4 Hz, 1H), 4.92 – 4.85 (m, 1H), 3.84 (d, *J* = 12.1 Hz, 1H), 3.76 (dd, *J* = 6.7, 3.9 Hz, 1H), 3.68 (dd, *J* = 11.6, 3.5 Hz, 1H), 3.61 (dd, *J* = 14.7, 3.3 Hz, 1H), 3.44 (ddd, *J* = 14.7, 12.5, 3.7 Hz, 1H), 3.24 (dd, *J* = 12.2, 3.9 Hz, 1H), 3.12 (ddd, *J* = 12.5, 11.5, 3.2 Hz, 1H), 2.43 (s, 3H), 2.02 (s, 3H). Spectral data match those reported in the literature.[4] **HPLC** (IB, 2-propanol/*n*-hexane = 35/65, flow rate = 1.0 mL/min, l = 210 nm) t_R = 5.9 min (major), 7.4 min (minor). **$[\alpha]_D^{20}$ =** +26.97 (*c* = 1.28, $CHCl_3$).

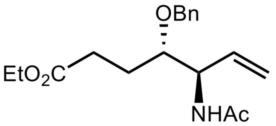

Ethyl (4S,5R)-5-acetamido-4-(benzyloxy)hept-6-enoate (**14**). Prepared using the General Procedure with (*S,S*)-**Rh-2** and purified with FCC (ethyl acetate/hexanes = 50/50) to provide a colorless oil (48.6 mg, 61% yield, 99.5:0.5 er). **^{1}H NMR** (400 MHz, $CDCl_3$) δ 7.39-7.29 (m, 5H), 5.80 (ddd, *J* = 17.1, 10.4, 6.6 Hz, 1H), 5.62 (d, *J* = 8.8 Hz, 1H), 5.25 (dt, *J* = 8.3, 1.4 Hz, 1H), 5.21 (d, *J* = 1.4 Hz, 1H), 4.68-4.61 (m,

2H), 4.52 (d, *J* = 11.6 Hz, 1H), 4.11 (q, *J* = 7.1 Hz, 2H), 3.52 (ddd, *J* = 7.7, 5.8, 3.5 Hz, 1H), 2.47-2.33 (m, 2H), 1.97-1.76 (m, 5H), 1.24 (t, *J* = 7.2 Hz, 3H). **^{13}C NMR** (101 MHz, $CDCl_3$) δ 173.4, 169.6, 138.3, 133.8, 128.7, 128.2, 128.1, 117.8, 79.5, 72.2, 60.6, 53.2, 30.3, 25.7, 23.5, 14.3. **HRMS:** (+APCI) calculated for $C_{18}H_{26}O_4N$ $[M+H]^+$ 320.1856, found 320.1853. **HPLC** (IK, 2-propanol/*n*-hexane = 20/80, flow rate = 1.0 mL/min, l = 210 nm) t_R = 6.7 min (major), t_m = 7.5 min (minor). **$[\alpha]_D^{20}$ =** +22.04 (*c* = 0.93, $CHCl_3$).

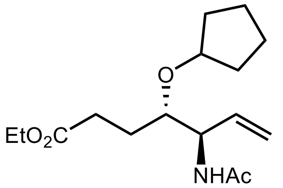

Ethyl (4S,5R)-5-acetamido-4-(cyclopentyloxy)hept-6-enoate (**15**). Prepared using the General Procedure with (*S,S*)-**Rh-2** and purified with FCC (acetone/hexanes = 20/80) to provide a colorless oil (30.8 mg, 41% yield, 99:1 er). **^{1}H NMR** (400 MHz, $CDCl_3$) δ 5.80-5.71 (m, 2H), 5.23 (dt, *J* = 13.8, 1.3 Hz, 1H), 5.19 (dt, *J* = 6.9, 1.4 Hz, 1H), 4.63 (dddd, *J* = 8.5, 6.5, 3.0, 1.4 Hz, 1H), 4.12 (q, *J* = 7.2 Hz, 2H), 4.05-4.01 (m, 1H), 3.43 (td, *J* = 6.7, 3.8 Hz, 1H), 2.44-2.31 (m, 2H), 2.01 (s, 3H), 1.77-1.50 (m, 10H), 1.25 (t, *J* = 7.1 Hz, 3H). **^{13}C NMR** (101 MHz, $CDCl_3$) δ 173.5, 169.5, 134.3, 117.4, 80.0, 60.6, 53.3, 32.8, 32.6, 30.3, 25.6, 23.7, 23.4, 23.4, 14.4. **HRMS:** (+APCI) calculated for $C_{16}H_{28}O_4N$ $[M+H]^+$ 298.2013, found 298.2010. **HPLC** (IH, 2-propanol/*n*-hexane = 20/80, flow rate = 1.0 mL/min, l = 210 nm) t_R = 5.6 min (minor), 9.0 min (major). **$[\alpha]_D^{20}$ =** +14.33 (*c* = 1.02, $CHCl_3$).

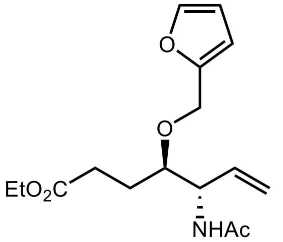

Ethyl (4R,5S)-5-acetamido-4-(furan-2-ylmethoxy)hept-6-enoate (**16**). Prepared using the General Procedure and purified with FCC (acetone/hexanes = 20/80) to provide a yellow oil (27.9 mg, 36% yield, 99.5:0.5 er). **^{1}H NMR** (400 MHz, $CDCl_3$) δ 7.43 (dd, *J* = 1.8, 0.8 Hz, 1H), 6.36 (dd, *J* = 3.2, 1.8 Hz, 1H), 6.34 (dd, *J* = 3.3, 0.8 Hz, 1H) 5.83-5.73 (m, 2H), 5.25 (dt, *J* = 10.9, 1.4 Hz, 1H), 5.22 (dt, *J* = 4.0, 1.3 Hz, 1H), 4.61 (d, *J* = 13.2 Hz, 1H), 4.58-4.53 (m, 1H), 4.47 (d, *J* = 13.2 Hz, 1H), 4.13 (q, *J* = 7.1 Hz, 2H), 3.51 (ddd, *J* = 7.4, 6.1, 3.4 Hz, 1H), 2.42-2.29 (m, 2H), 1.93 (s, 3H), 1.90-1.81 (m, 1H), 1.80-1.71 (m, 1H), 1.25 (t, *J* = 7.1 Hz, 3H). **^{13}C NMR** (101 MHz, $CDCl_3$) δ 173.2, 169.4, 151.8, 143.2, 133.5, 118.2, 110.7, 110.1, 79.1, 64.1, 60.7, 53.6, 30.2, 26.0, 23.6, 14.4. **HRMS:** (+APCI) calculated for $C_{16}H_{24}O_5N$ $[M+H]^+$ 310.1649, found 310.1646. **HPLC** (IH, 2-propanol/*n*-hexane = 30/70, flow rate = 1.0 mL/min, l = 210 nm) t_R = 6.9 min (major), 11.3 min (minor). **$[\alpha]_D^{20}$ =** -1.90 (*c* = 0.20, $CHCl_3$).

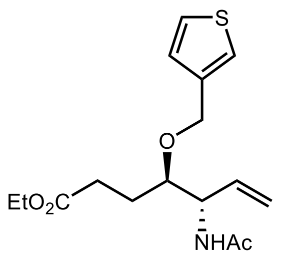

Ethyl (4R,5S)-5-acetamido-4-(thiophen-3-ylmethoxy)hept-6-enoate (**17**). Prepared using the General Procedure and purified with FCC (acetone/hexanes = 15/85) to provide a yellow oil (43.1 mg, 53% yield, 98:2 er). **^{1}H NMR** (400 MHz, $CDCl_3$) δ 7.33 (dd, *J* = 4.9, 3.0 Hz, 1H), 7.24 – 7.22 (m, 1H), 7.08 (dd, *J* = 4.9, 1.3 Hz, 1H), 5.78 (ddd, *J* = 17.1, 10.5, 6.5 Hz, 1H), 5.63 (d, *J* = 8.7 Hz, 1H), 5.24 (dt, *J* = 7.5, 1.3 Hz, 1H), 5.21 (d, *J* = 1.4 Hz, 1H), 4.67 – 4.51 (m, 3H), 4.11 (q, *J* = 7.1 Hz, 2H), 3.49 (ddd, *J* = 7.7, 5.7, 3.5 Hz, 1H), 2.47 – 2.27 (m, 2H), 1.89 (s, 3H), 1.88 – 1.71 (m, 2H), 1.24 (t, *J* = 7.1 Hz, 3H). **^{13}C NMR** (101 MHz, $CDCl_3$) δ 173.4, 169.5, 139.3, 133.8, 127.5, 126.6, 123.7, 117.8, 79.3, 67.1, 60.7, 53.2, 30.3, 25.7, 23.5, 14.3. **HRMS:** (+APCI) calculated for $C_{16}H_{24}O_4N^{32}S$ $[M+H]^+$ 326.1421, found 326.1419. **HPLC** (IK, 2-propanol/*n*-hexane = 20/80, flow rate = 1.0 mL/min, l = 210 nm) t_R = 7.6 min (minor), 8.6 min (major). **$[\alpha]_D^{20}$ =** -26.92 (*c* = 1.37, $CHCl_3$).

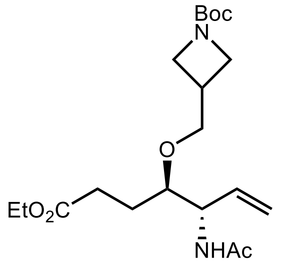

tert-*Butyl 3-(((3S,4R)-3-acetamido-7-ethoxy-7-oxohept-1-en-4-yl)oxy)methyl)azetidine-1-carboxylate* (**18**). Prepared using the General Procedure and purified with FCC (acetone/hexanes = 20/80) to provide a yellow oil (36.7 mg, 37% yield, 99:1 er). **^{1}H NMR** (400 MHz, $CDCl_3$) δ 5.80-5.71 (m, 2H), 5.26-5.20 (m, 2H), 4.72-4.67 (m, 1H), 4.12 (qd, *J* = 7.1, 1.3 Hz, 2H), 3.97 (td, *J* = 8.5, 1.3 Hz, 2H), 3.68-3.63 (m, 3H), 3.59-3.55 (m, 1H), 3.44-3.40 (m, 1H), 2.74-2.64 (m, 1H), 2.45-2.30 (m, 2H), 2.02 (d, *J* = 1.3 Hz, 3H), 1.79-1.73 (m, 2H), 1.43 (d, *J* = 1.3 Hz, 9H), 1.25 (td, *J* = 7.1, 1.2 Hz, 3H). **^{13}C NMR** (101 MHz, $CDCl_3$) δ 173.3, 169.7, 156.6, 134.0, 117.8, 80.5, 79.6, 71.4, 60.7, 53.2, 30.2, 29.0, 28.5, 28.5, 25.3, 23.6, 14.4. **HRMS:** (+APCI) calculated for $C_{20}H_{35}O_6N_2$ $[M+H]^+$ 399.2490, found 399.2491. **HPLC** (IB, 2-propanol/*n*-hexane = 20/80, flow rate = 1.0 mL/min, l = 210 nm) t_R = 4.9 min (minor), 6.1 min (major). **$[\alpha]_D^{20}$ =** -12.22 (*c* = 1.20, $CHCl_3$).

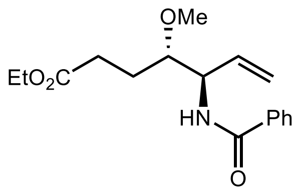

Ethyl (4S,5R)-5-benzamido-4-methoxyhept-6-enoate (**19**). Prepared using the General Procedure with 4 mol% (*S,S*)-**Rh-2** and 20 mol% $AgBF_4$ and purified with FCC (acetone/hexanes = 20/80) to provide a yellow oil (23.4 mg, 31% yield, 96:4 er). **^{1}H NMR** (400 MHz, $CDCl_3$) δ 7.82 – 7.77 (m, 2H), 7.54 – 7.49 (m, 1H), 7.47 – 7.42 (m, 2H), 6.50 (d, *J* = 8.5 Hz, 1H), 5.88 (ddd, *J* = 17.1, 10.4, 6.6 Hz, 1H), 5.33 (dt, *J* = 17.2, 1.3 Hz, 1H), 5.28 (dt, *J* = 10.4, 1.3 Hz, 1H), 4.84 (dddd, *J* = 8.2, 6.6, 3.6, 1.8 Hz, 1H), 4.14 (q, *J* = 7.2 Hz, 2H), 3.44 (s, 3H), 3.41 (ddd, *J* = 8.2, 4.9, 2.3 Hz, 1H), 2.48 – 2.40 (m, 2H), 1.92 (dq, *J* = 14.6, 7.4

Hz, 1H), 1.87 – 1.76 (m, 1H), 1.26 (t, *J* = 7.2 Hz, 3H). **^{13}C NMR** (101 MHz, $CDCl_3$) δ 173.4, 166.9, 134.6, 133.7, 131.7, 128.8, 127.1, 118.1, 82.0, 60.7, 58.4, 53.8, 30.4, 25.5, 14.4. **HRMS:** (+APCI) calculated for $C_{17}H_{24}O_4N$ $[M+H]^+$ 306.1700, found 306.1701. **HPLC** (IH, 2-propanol/*n*-hexane = 20/80, flow rate = 1.0 mL/min, l = 210 nm) t_R = 6.0 min (minor), 7.8 min (major). **$[\alpha]_D^{20}$ =** +25.15 (*c* = 1.13, $CHCl_3$).

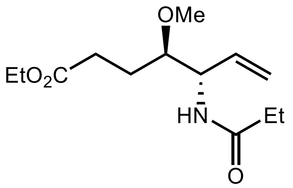

Ethyl (4R,5S)-4-methoxy-5-propionamidohept-6-enoate (**20**). Prepared using the General Procedure and purified with FCC (acetone/pentane = 15/85) to provide a yellow oil (55.4 mg, 86% yield, 99:1 er). **^{1}H NMR** (400 MHz, $CDCl_3$) δ 5.81-5.73 (m, 2H), 5.24 (dt, *J* = 9.8, 1.4 Hz, 1H), 5.20 (qn, *J* = 1.4 Hz, 1H), 4.64 (dddd, *J* = 9.9, 6.5, 3.0, 1.4 Hz, 1H), 4.12 (q, *J* = 7.1 Hz, 2H), 3.39 (s, 3H), 3.28 (ddd, *J* = 7.6, 5.8, 3.8 Hz, 1H), 2.38 (ddd, *J* = 8.1, 6.9, 4.0 Hz, 2H), 2.24 (q, *J* = 7.6 Hz, 2H), 1.87-1.78 (m, 1H), 1.77-1.68 (m, 1H), 1.25 (t, *J* = 7.1 Hz, 3H), 1.16 (t, *J* = 7.6 Hz, 3H). **^{13}C NMR** (101 MHz, $CDCl_3$) δ 173.4, 173.3, 133.9, 117.6, 81.9, 60.6, 58.2, 53.0, 30.3, 30.0, 25.3, 14.3, 10.0. **HRMS:** (+APCI) calculated for $C_{13}H_{24}O_4N$ $[M+H]^+$ 258.1700, found 258.1699. **HPLC** (IK, 2-propanol/*n*-hexane = 10/90, flow rate = 1.0 mL/min, l = 210 nm) t_R = 12.4 min (minor), 13.7 min (major). **$[\alpha]_D^{20}$ =** -9.17 (*c* = 0.29, $CHCl_3$).

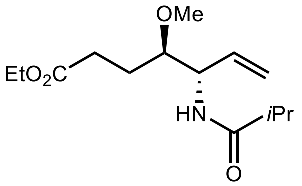

Ethyl (4R,5S)-5-isobutyramido-4-methoxyhept-6-enoate (**21**). Prepared using the General Procedure and purified with FCC (acetone/hexanes = 15/85) to provide a yellow oil (31.7 mg, 47% yield, 98:2 er). **^{1}H NMR** (400 MHz, $CDCl_3$) δ 5.83-5.75 (m, 2H), 5.24 (dt, *J* = 8.6, 1.4 Hz, 1H), 5.21 (dt, *J* = 1.8, 1.0 Hz, 1H), 4.65-4.60 (m, 1H), 4.13 (q, *J* = 7.1 Hz, 2H), 3.40 (s, 3H), 3.29 (ddd, *J* = 7.4, 5.8, 3.8 Hz, 1H), 2.46-2.33 (m, 3H), 1.88-1.70 (m, 2H), 1.25 (t, *J* = 7.1 Hz, 3H), 1.17 (dd, *J* = 6.9, 0.9 Hz, 6H). **^{13}C NMR** (101 MHz, $CDCl_3$) δ 176.4, 173.4, 133.9, 117.6, 82.0, 60.6, 58.3, 52.9, 35.9, 30.4, 25.4, 19.8, 19.8, 14.4. **HRMS:** (+APCI) calculated for $C_{14}H_{26}O_4N$ $[M+H]^+$ 272.1856, found 272.1855. **HPLC** (IA, 2-propanol/*n*-hexane = 10/90, flow rate = 1.0 mL/min, l = 210 nm) t_R = 6.3 min (minor), 7.4 min (major). **$[\alpha]_D^{20}$ =** -12.19 (*c* = 0.97, $CHCl_3$).

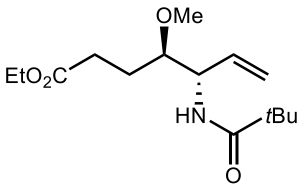

Ethyl (4R,5S)-4-methoxy-5-pivalamidohept-6-enoate (**22**). Prepared using the General Procedure and purified with FCC (acetone/hexanes = 15/85) to provide a brown oil (32.2 mg, 45% yield, 95:5 er). Isolated as a ~2:1 mixture of diastereomers. **^{1}H NMR** (400 MHz, $CDCl_3$) δ 5.98-5.76 (m, 2H), 5.23 (dt, *J* = 4.8, 1.4 Hz, 1H, major), 5.19 (qn, *J* = 1.4 Hz, 1H, major), 5.18-5.16 (m, 1H, minor), 5.13 (d, *J* = 1.8 Hz, 1H, minor) 4.61-4.55 (m, 1H), 4.12 (q, *J* = 7.1 Hz, 2H, major), 4.12 (q, *J* = 7.1 Hz, 2H, minor), 3.39 (s,

3H), 3.33 (ddd, J = 7.8, 5.7, 2.4 Hz, 1H, minor), 3.28 (ddd, J = 7.4, 5.9, 3.8 Hz, 1H, major), 2.45-2.31 (m, 2H), 1.87-1.62 (m, 2H), 1.25 (t, J = 7.2 Hz, 3H, major), 1.24 (t, J = 7.2 Hz, 3H, minor), 1.23 (s, 9H, minor), 1.21 (s, 9H, major). **^{13}C NMR** (101 MHz, $CDCl_3$) δ 178.2 (minor), 177.9 (major), 173.5 (minor), 173.4 (major), 136.6 (minor), 134.0 (major), 117.5 (major), 115.4 (minor), 82.1 (major), 81.9 (minor), 60.6 (major), 60.6 (minor), 59.0 (minor), 58.5 (major), 53.1 (major), 52.7 (minor), 39.0 (minor), 38.9 (major), 30.5 (minor), 30.4 (major), 27.8 (minor), 27.7 (major), 26.5 (minor), 25.6 (major), 14.4 (minor), 14.3 (major). **HRMS:** (+APCI) calculated for $C_{15}H_{28}O_4N$ $[M+H]^+$ 286.2013, found 286.2017. **HPLC** (IK, 2-propanol/*n*-hexane = 3/97, flow rate = 1.0 mL/min, l = 210 nm) t_R = 21.2 min (minor), 23.1 min (major). **$[\alpha]_D^{20}$ =** +3.30 (c = 0.86, $CHCl_3$).

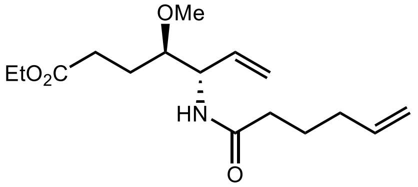

Ethyl (4R,5S)-5-(hex-5-enamido)-4-methoxyhept-6-enoate (**23**). Prepared using the General Procedure and purified with FCC (acetone/hexanes = 15/85) to provide a colorless oil (51.6 mg, 69% yield, 99:1 er). **^{1}H NMR** (400 MHz, $CDCl_3$) δ 5.83-5.73 (m, 3H), 5.26-5.23 (m, 1H), 5.21 (qn, J = 1.4 Hz, 1H), 5.06-4.97 (m, 2H), 4.66 (dddd, J = 8.6, 6.4, 3.8, 1.9 Hz, 1H), 4.13 (q, J = 7.1 Hz, 2H), 3.40 (s, 3H), 3.28 (ddd, J = 7.6, 5.8, 3.7 Hz, 1H), 2.46-2.33 (m, 2H), 2.22 (dd, J = 8.0, 6.6 Hz, 2H), 2.13-2.07 (m, 2H), 1.87-1.69 (m, 4H), 1.26 (t, J = 7.2 Hz, 3H). **^{13}C NMR** (101 MHz, $CDCl_3$) δ 173.4, 172.3, 138.0, 133.9, 117.7, 115.5, 81.9, 60.7, 58.2, 53.0, 36.2, 33.2, 30.3, 25.3, 24.9, 14.4. **HRMS:** (+APCI) calculated for $C_{16}H_{28}O_4N$ $[M+H]^+$ 298.2013, found 298.2016. **HPLC** (IK, 2-propanol/*n*-hexane = 15/85, flow rate = 1.0 mL/min, l = 210 nm) t_R = 6.6 min (minor), 7.4 min (major). **$[\alpha]_D^{20}$ =** -13.02 (c = 0.63, $CHCl_3$).

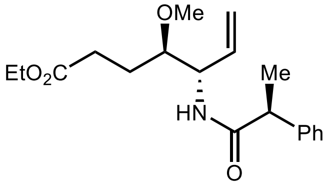

Ethyl (4R,5S)-4-methoxy-5-((S)-2-phenylpropanamido)hept-6-enoate (**24a**). Prepared using the General Procedure and purified with FCC (acetone/hexanes = 10/90) to provide a brown oil (20.4 mg, 25% yield, 99:1 er). **^{1}H NMR** (400 MHz, $CDCl_3$) δ 7.37-7.28 (m, 5H), 5.71 (ddd, J = 17.1, 10.5, 6.6 Hz, 1H), 5.63 (d, J = 8.7 Hz, 1H), 5.19-5.12 (m, 2H), 4.54 (dddd, J = 8.5, 6.4, 4.4, 2.9 Hz, 1H), 4.11 (q, J = 7.1 Hz, 2H), 3.59 (q, J = 7.2 Hz, 1H), 3.13 (s, 3H), 3.12-3.08 (m, 1H), 2.31 (t, J = 7.8 Hz, 2H), 1.73-1.59 (m, 2H), 1.54 (d, J = 7.2 Hz, 3H), 1.24 (t, J = 7.1 Hz, 3H). **^{13}C NMR** (101 MHz, $CDCl_3$) δ 173.5, 173.3, 141.5, 133.6, 129.1, 127.8, 127.5, 117.8, 82.0, 60.6, 58.4, 53.5, 47.4, 30.3, 25.6, 18.4, 14.4. **HRMS:** (+APCI) calculated for $C_{19}H_{28}O_4N$ $[M+H]^+$ 334.2013, found 334.2015. **HPLC** (IH, 2-propanol/*n*-hexane = 10/90, flow rate = 1.0 mL/min, l = 210 nm) t_R = 6.9 min (major), 8.6 min (minor). **$[\alpha]_D^{20}$ =** -8.24 (c = 0.51, $CHCl_3$).

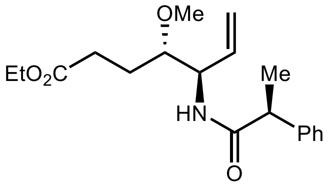

Ethyl (4S,5R)-4-methoxy-5-((S)-2-phenylpropanamido)hept-6-enoate (**24b**). Prepared using the General Procedure with (*S,S*)-**Rh-2** and purified with FCC (acetone/hexanes = 10/90) to provide a brown oil (18.8 mg, 23% yield, 94:6 er). **^{1}H NMR** (400 MHz, $CDCl_3$) δ 7.37-7.27 (m, 5H), 5.66 (ddd, *J* = 17.2, 10.5, 6.0 Hz, 1H), 5.58 (d, *J* = 8.8 Hz, 1H), 5.08 (dt, *J* = 10.5, 1.4 Hz, 1H), 4.95 (dt, *J* = 17.2, 1.4 Hz, 1H), 4.61 (dddd, *J* = 10.3, 5.5, 3.6, 1.8 Hz, 1H), 4.12 (q, *J* = 7.1 Hz, 2H), 3.61 (q, *J* = 7.2 Hz, 1H), 3.28 (s, 3H), 3.19 (ddd, *J* = 7.3, 5.8, 3.8 Hz, 1H), 2.42-2.28 (m, 2H), 1.71-1.64 (m, 2H), 1.54 (d, *J* = 7.2 Hz, 3H), 1.25 (t, *J* = 7.1 Hz, 3H). **^{13}C NMR** (101 MHz, $CDCl_3$) δ 173.7, 173.4, 141.4, 133.7, 129.1, 127.8, 127.5, 117.2, 82.0, 60.6, 58.4, 52.9, 47.4, 30.3, 25.4, 18.4, 14.4. **HRMS:** (+APCI) calculated for $C_{19}H_{28}O_4N$ $[M+H]^+$ 334.2013, found 334.2013. **HPLC** (IH, 2-propanol/*n*-hexane = 10/90, flow rate = 1.0 mL/min, l = 210 nm) t_R = 7.0 min (minor), 8.5 min (major). **$[\alpha]_D^{20}$ =** +27.13 (*c* = 0.32, $CHCl_3$).

8. NMR Spectra and HPLC Traces for New Compounds

*(R,R)-(η^3-2,4,5,6,7-pentamethyl-3-phenylinden-1H-yl) rhodium(III) iodide dimer ((R,R)-***Rh-2***):*

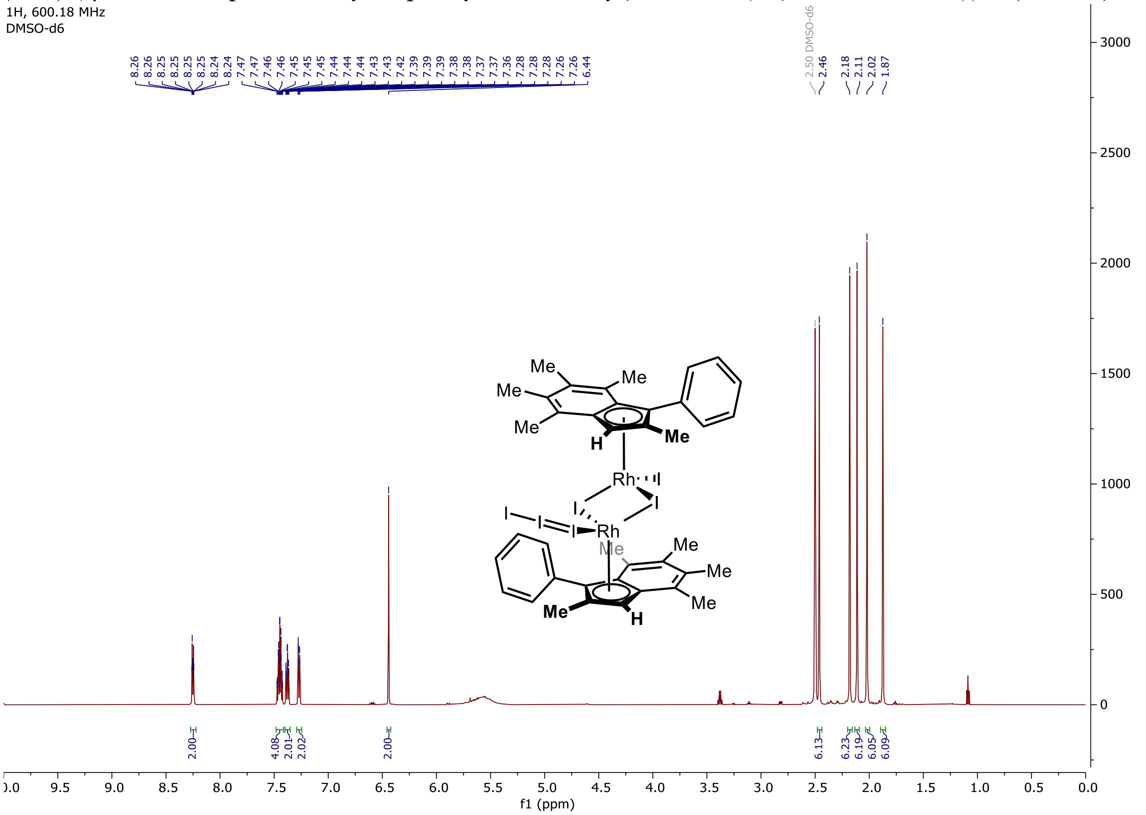

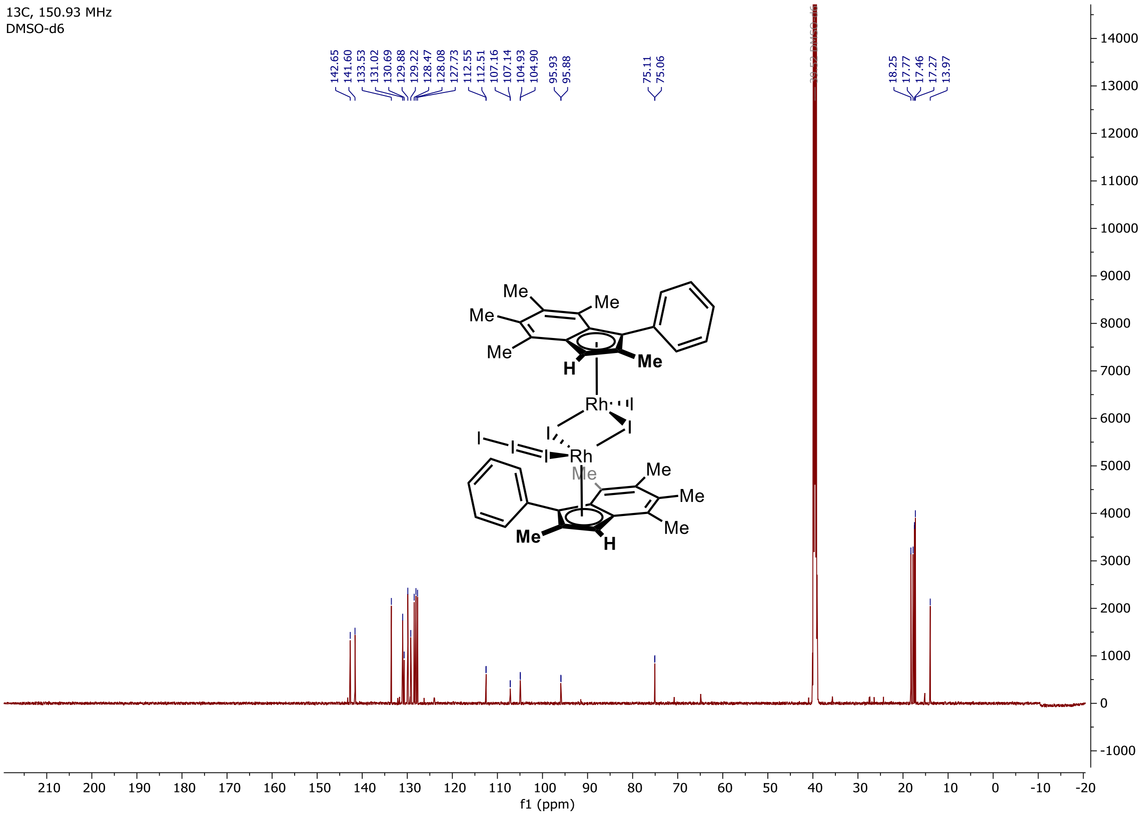

N-((3S,4R)-4-methoxynon-1-en-3-yl)acetamide (**3**):

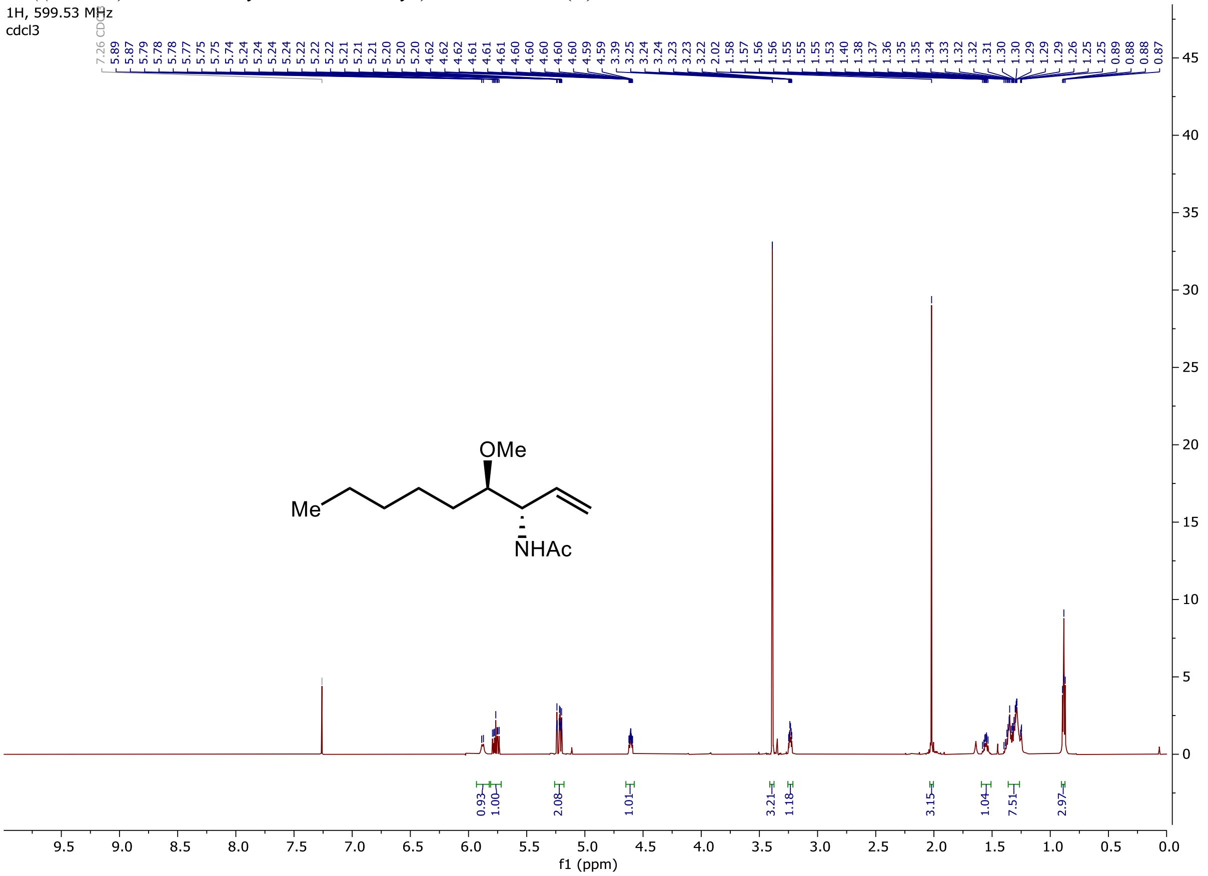

N-((±)-4-methoxynon-1-en-3-yl)acetamide (±-**3**):

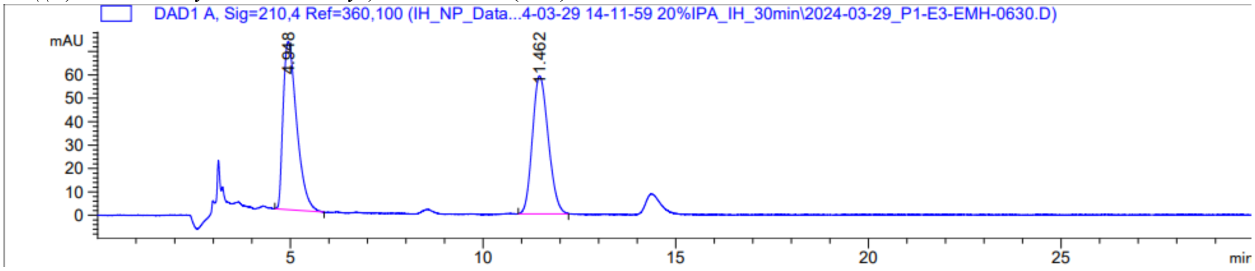

Peak #	RetTime [min]	Type	Width [min]	Area [mAU*s]	Height [mAU]	Area %
1	4.948	BV R	0.2872	1757.35938	72.01173	51.0286
2	11.462	BV R	0.3353	1686.50952	59.02794	48.9714

N-((3S,4R)-4-methoxynon-1-en-3-yl)acetamide (**3**):

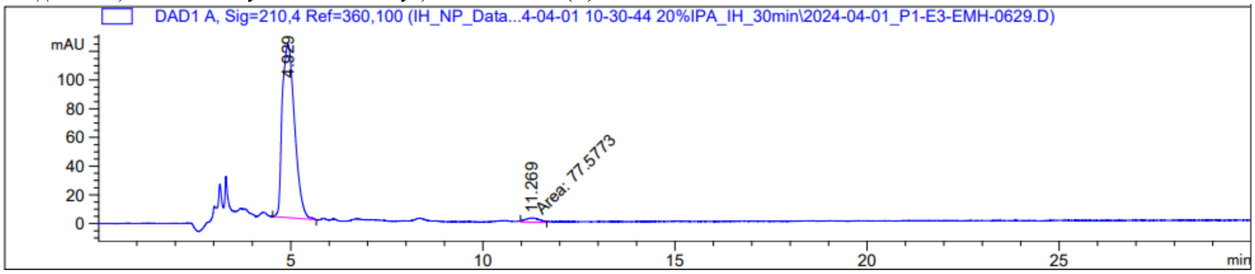

Peak #	RetTime [min]	Type	Width [min]	Area [mAU*s]	Height [mAU]	Area %
1	4.929	BV R	0.2633	2692.56836	120.91361	97.1995
2	11.269	MM	0.4375	77.57726	2.95529	2.8005

N-((3S,4R)-4-methoxypent-1-en-3-yl)acetamide (**4**):

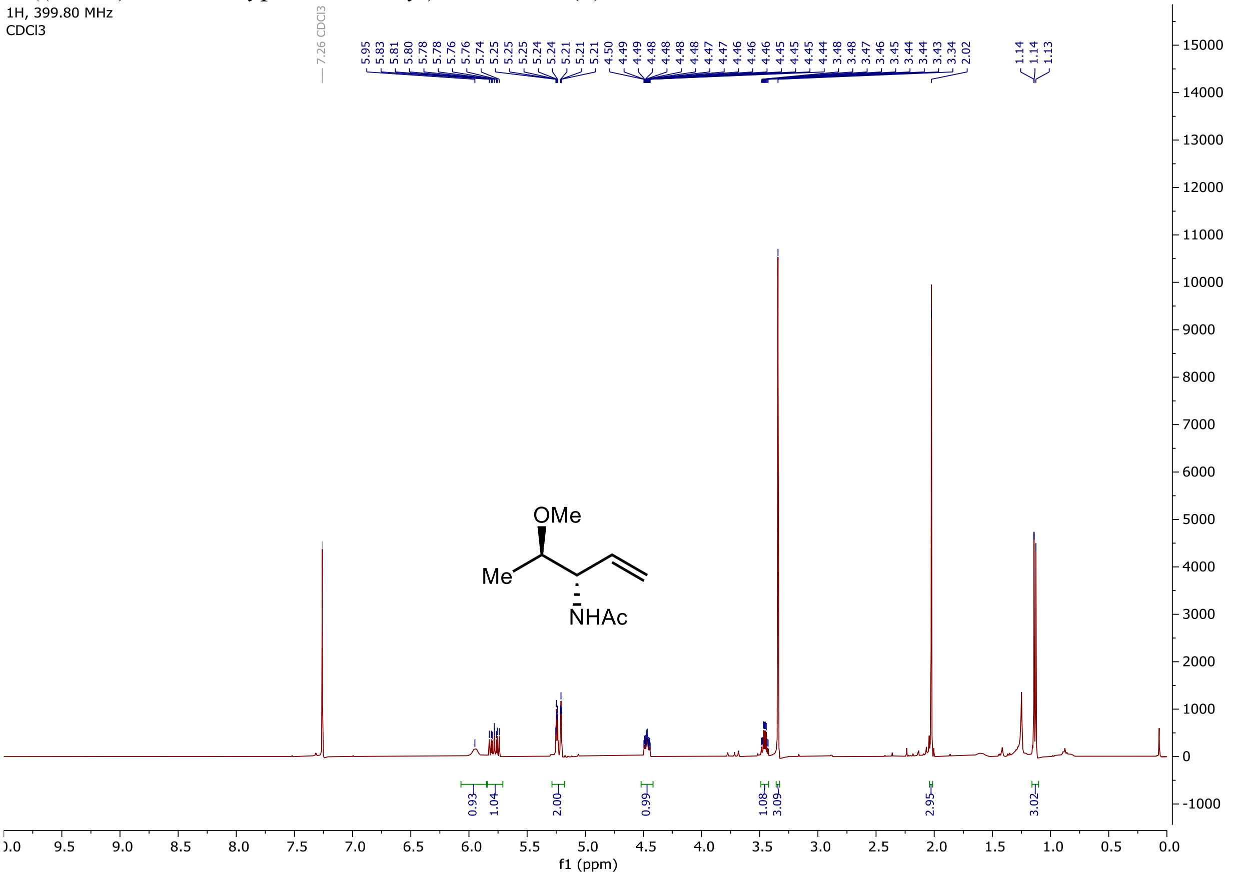

N-((±)-4-methoxypent-1-en-3-yl)acetamide (±-**4**):

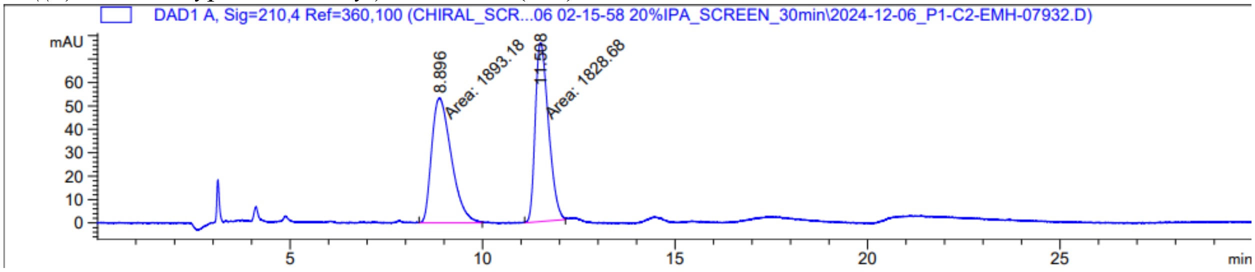

Peak #	RetTime [min]	Type	Width [min]	Area [mAU*s]	Height [mAU]	Area %
1	8.896	MM	0.5917	1893.17749	53.32618	50.8664
2	11.508	MM	0.3985	1828.68420	76.48618	49.1336

N-((3S,4R)-4-methoxypent-1-en-3-yl)acetamide (**4**):

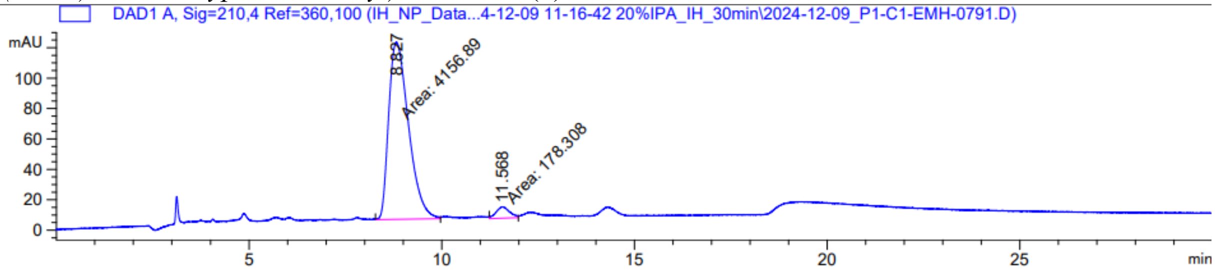

Peak #	RetTime [min]	Type	Width [min]	Area [mAU*s]	Height [mAU]	Area %
1	8.827	MM	0.5937	4156.88965	116.69800	95.8870
2	11.568	MM	0.3973	178.30843	7.48071	4.1130

Ethyl (4R,5S)-5-acetamido-4-methoxyhept-6-enoate (**5**):

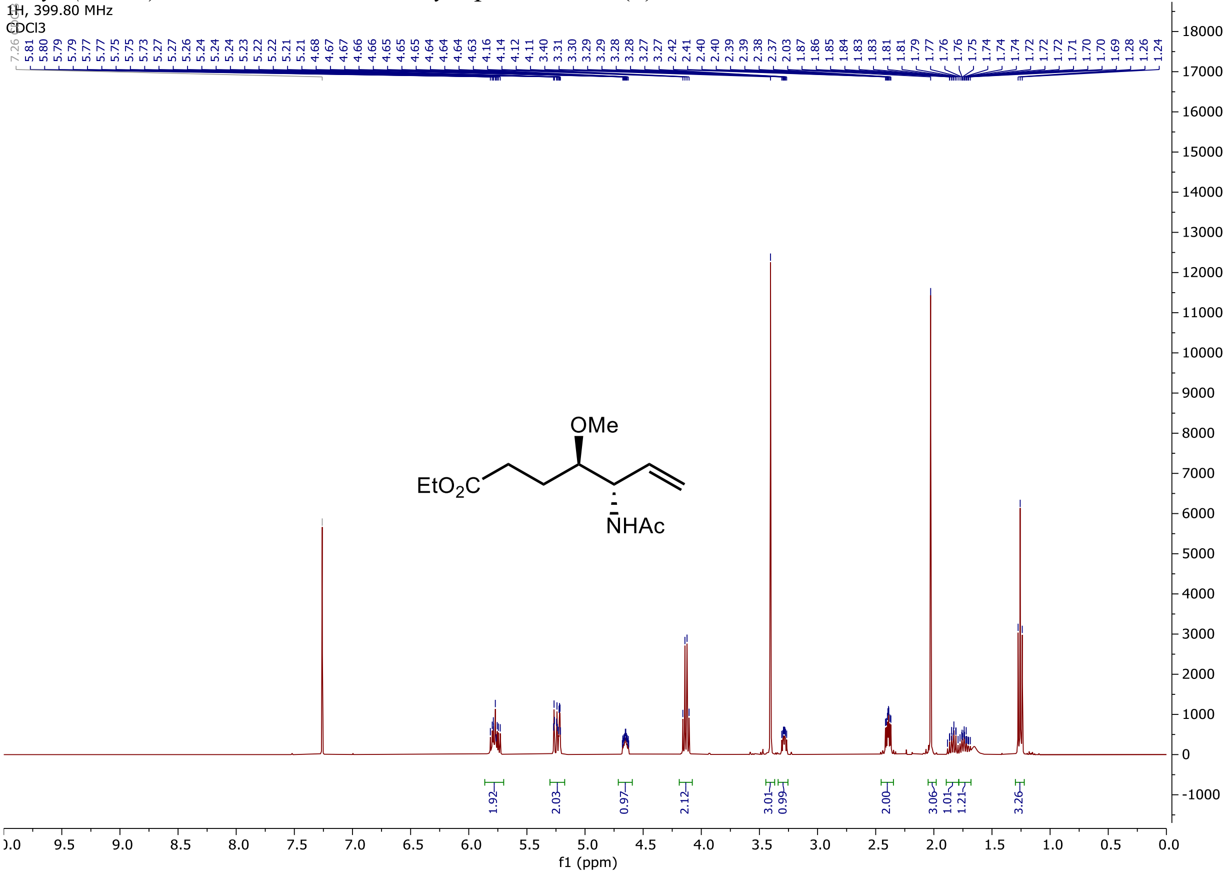

Ethyl (±)-5-acetamido-4-methoxyhept-6-enoate (±-**5**):

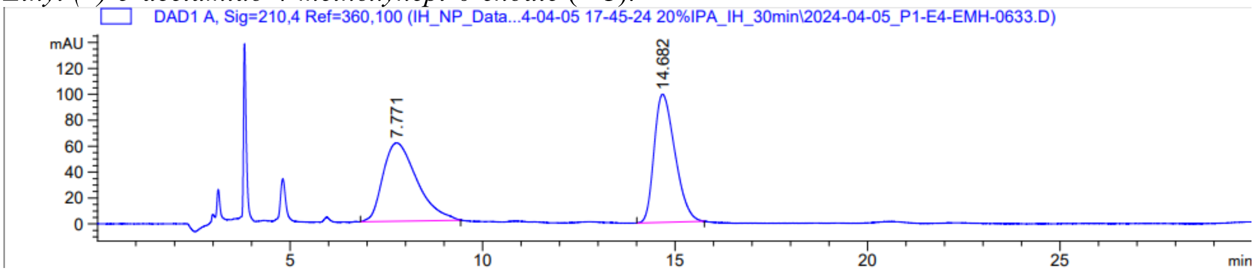

Peak #	RetTime [min]	Type	Width [min]	Area [mAU*s]	Height [mAU]	Area %
1	7.771	BV R	0.7178	3715.45483	60.51184	49.9736
2	14.682	VV R	0.4406	3719.37671	98.92846	50.0264

Ethyl (4R,5S)-5-acetamido-4-methoxyhept-6-enoate (**5**):

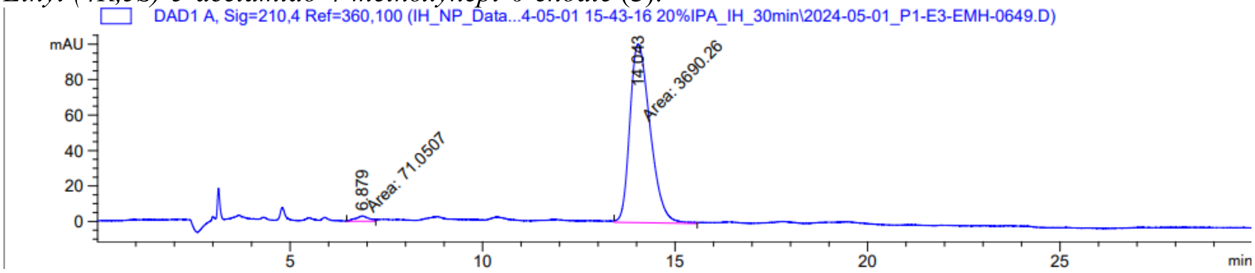

Peak #	RetTime [min]	Type	Width [min]	Area [mAU*s]	Height [mAU]	Area %
1	6.879	MM	0.4005	71.05071	2.95643	1.8890
2	14.043	MM	0.6101	3690.26392	100.81067	98.1110

N-((3S,4R)-7-((N,4-dimethylphenyl)sulfonamido)-4-methoxyhept-1-en-3-yl)acetamide (**6**):

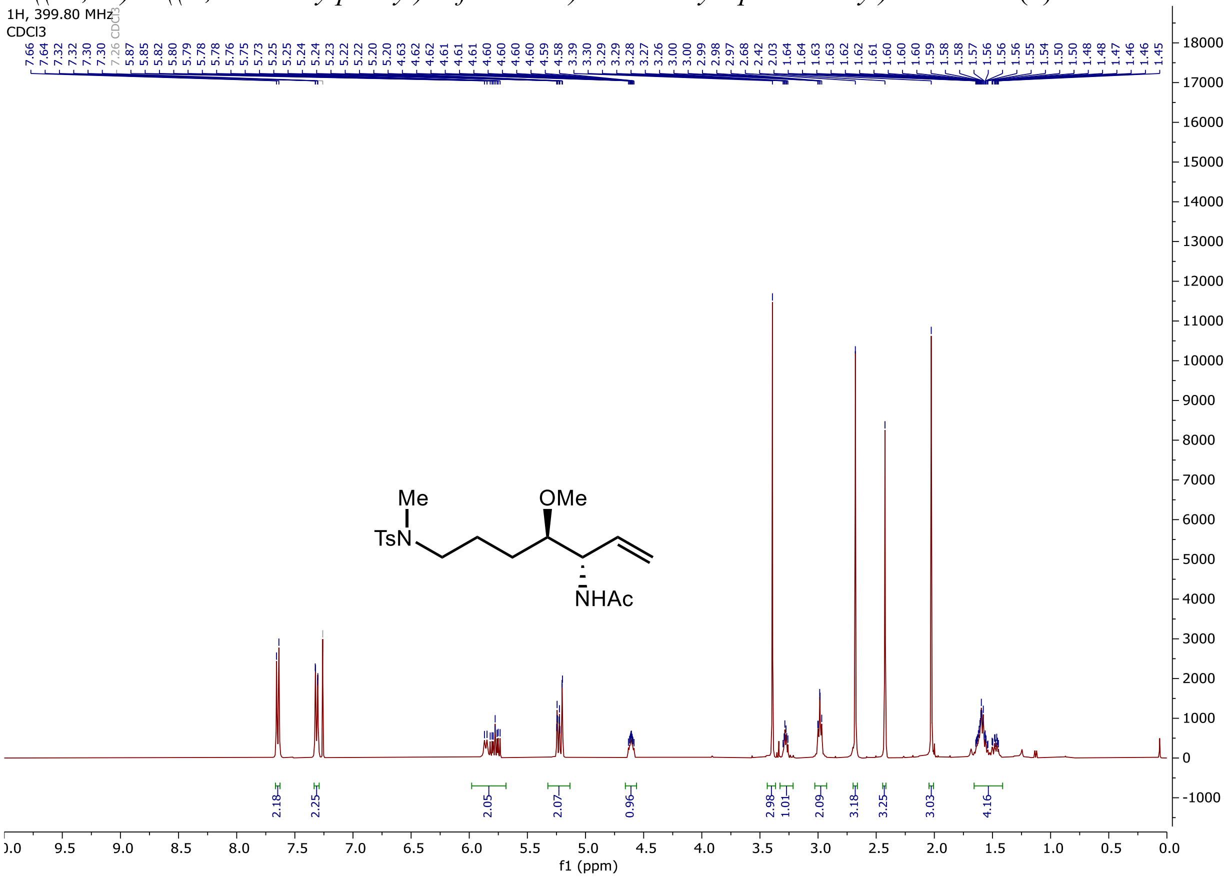

N-((±)-7-((N,4-dimethylphenyl)sulfonamido)-4-methoxyhept-1-en-3-yl)acetamide (±-**6**):

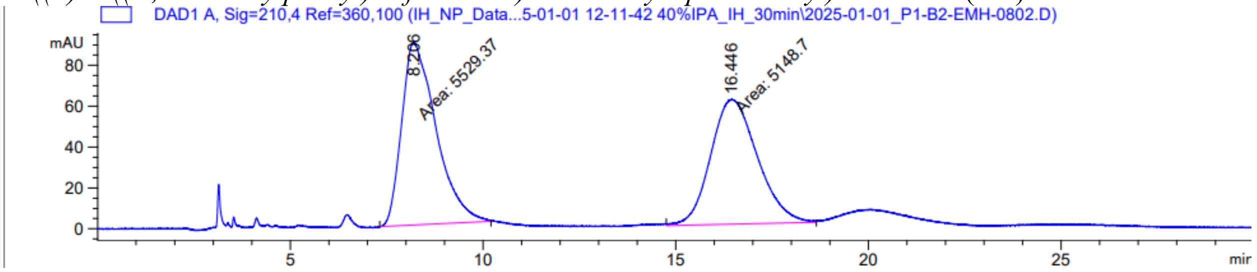

Peak #	RetTime [min]	Type	Width [min]	Area [mAU*s]	Height [mAU]	Area %
1	8.206	MM	1.0319	5529.37158	89.30863	51.7825
2	16.446	MM	1.4042	5148.70068	61.11019	48.2175

N-((3S,4R)-7-((N,4-dimethylphenyl)sulfonamido)-4-methoxyhept-1-en-3-yl)acetamide (**6**):

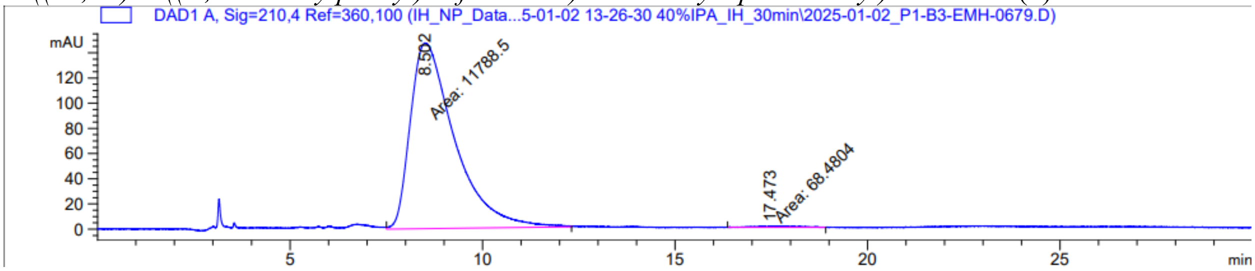

Peak #	RetTime [min]	Type	Width [min]	Area [mAU*s]	Height [mAU]	Area %
1	8.502	MM	1.3318	1.17885e4	147.52490	99.4224
2	17.473	MM	1.1816	68.48037	9.65918e-1	0.5776

(4R,5S)-5-acetamido-4-methoxyhept-6-en-1-yl acetate (**7**):

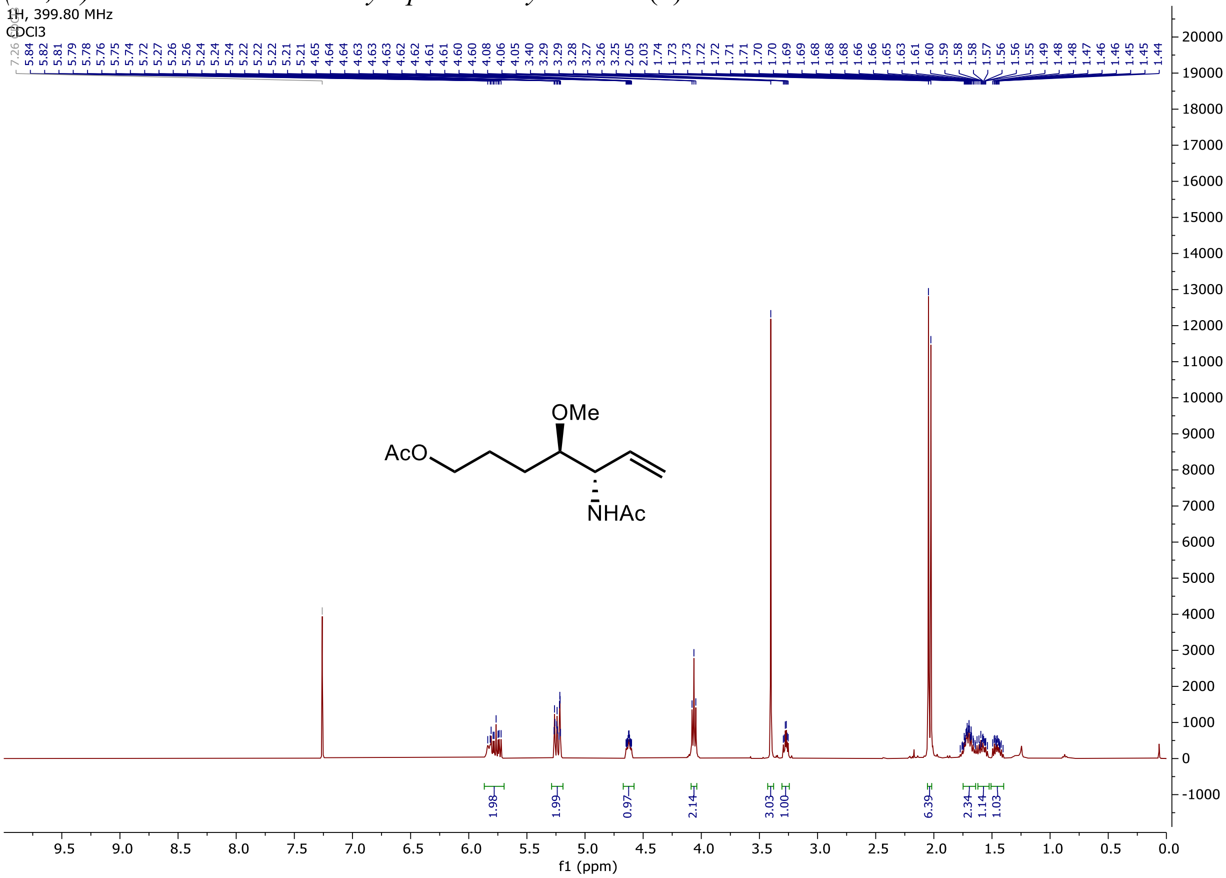

(±)-5-acetamido-4-methoxyhept-6-en-1-yl acetate (±-**7**):

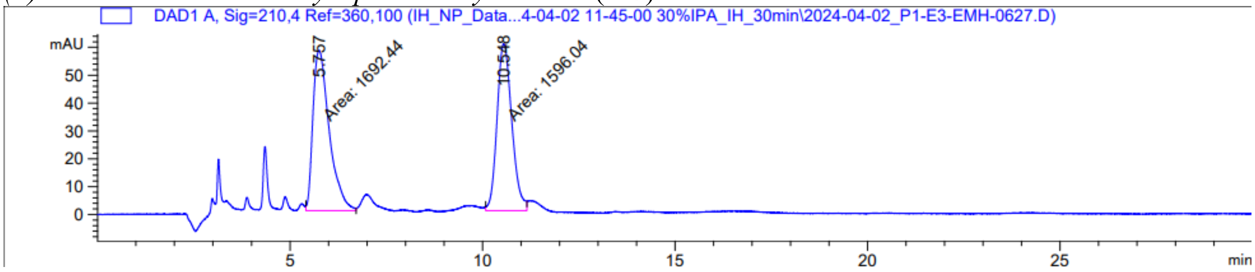

Peak #	RetTime [min]	Type	Width [min]	Area [mAU*s]	Height [mAU]	Area %
1	5.757	MM	0.4897	1692.43994	57.59734	51.4657
2	10.548	MM	0.4440	1596.04016	59.90839	48.5343

(4R,5S)-5-acetamido-4-methoxyhept-6-en-1-yl acetate (**7**):

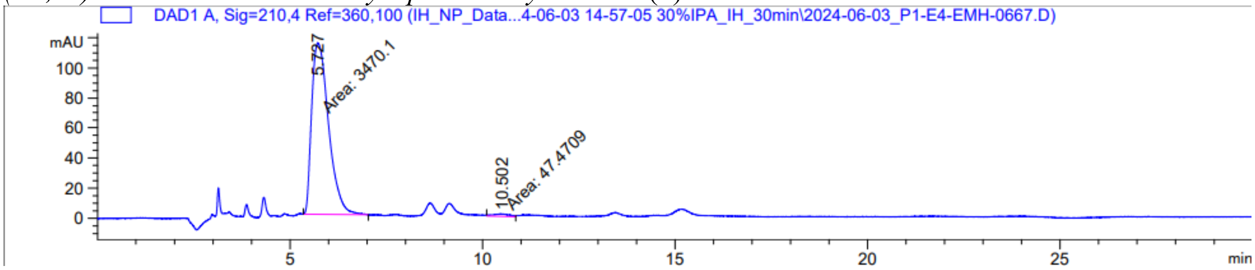

Peak #	RetTime [min]	Type	Width [min]	Area [mAU*s]	Height [mAU]	Area %
1	5.727	MM	0.5076	3470.10278	113.93488	98.6505
2	10.502	MM	0.4892	47.47094	1.61720	1.3495

N-((3S,4R)-7-bromo-4-methoxyhept-1-en-3-yl)acetamide (**8**):

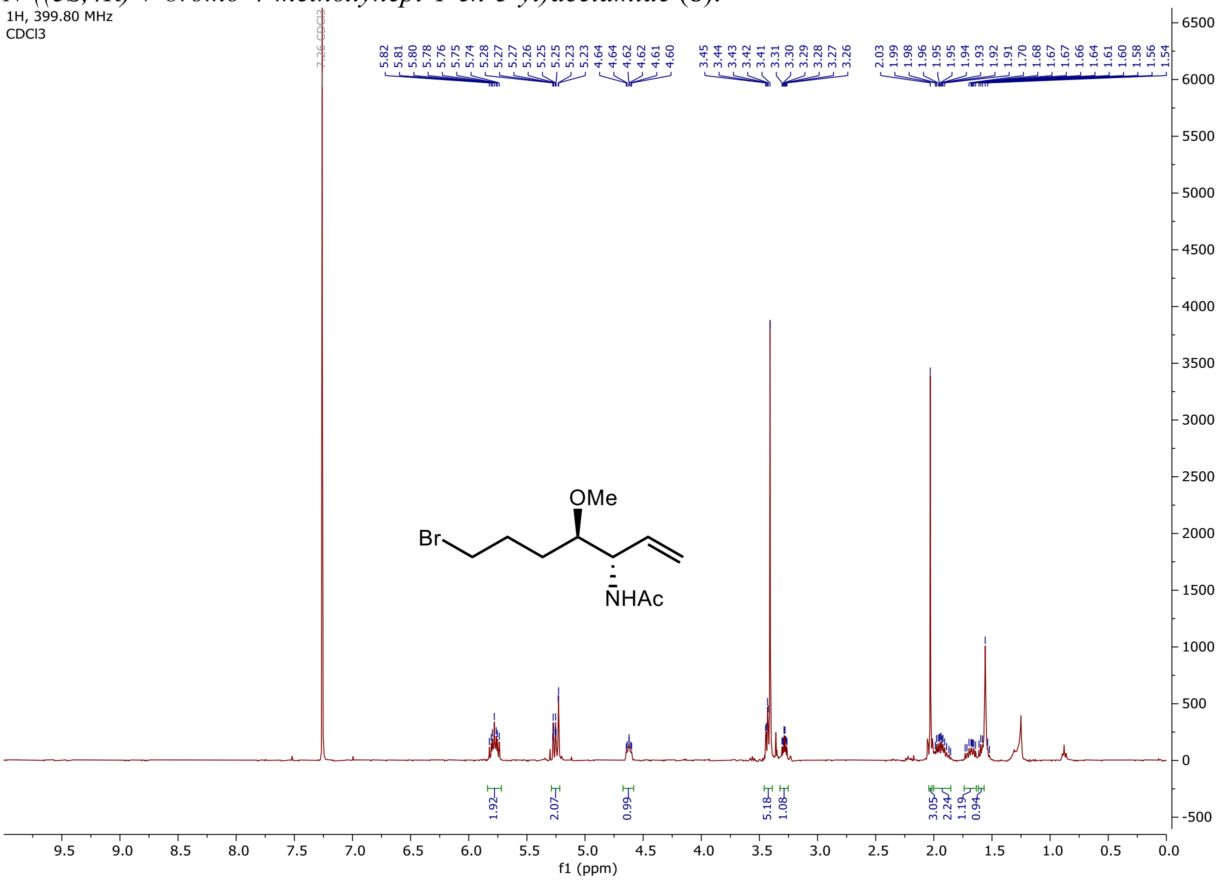

N-((±)-7-bromo-4-methoxyhept-1-en-3-yl)acetamide (±-**8**):

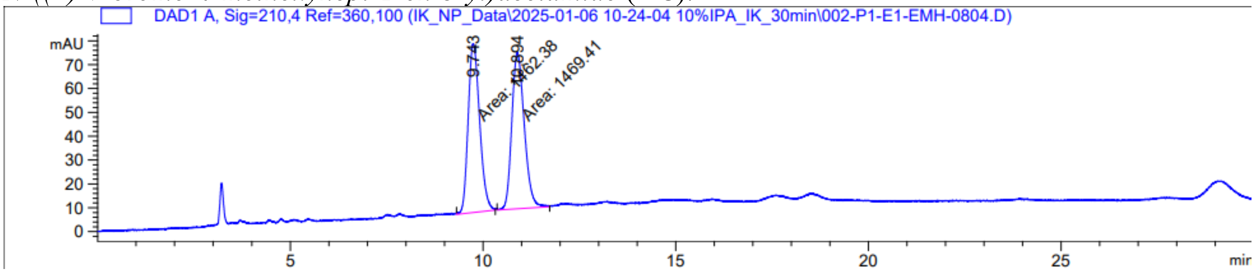

Peak #	RetTime [min]	Type	Width [min]	Area [mAU*s]	Height [mAU]	Area %
1	9.743	MM	0.3443	1462.37671	70.78133	49.8800
2	10.894	MM	0.3743	1469.41089	65.43211	50.1200

N-((3S,4R)-7-bromo-4-methoxyhept-1-en-3-yl)acetamide (**8**):

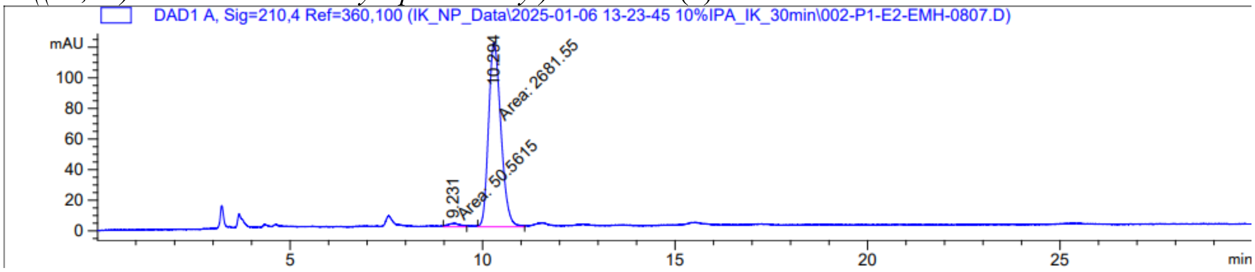

Peak #	RetTime [min]	Type	Width [min]	Area [mAU*s]	Height [mAU]	Area %
1	9.231	MM	0.3673	50.56152	2.29449	1.8506
2	10.294	MM	0.3724	2681.55347	120.02538	98.1494

N-((1S,2R)-1-methoxy-1-phenylbut-3-en-2-yl)acetamide (**9**):

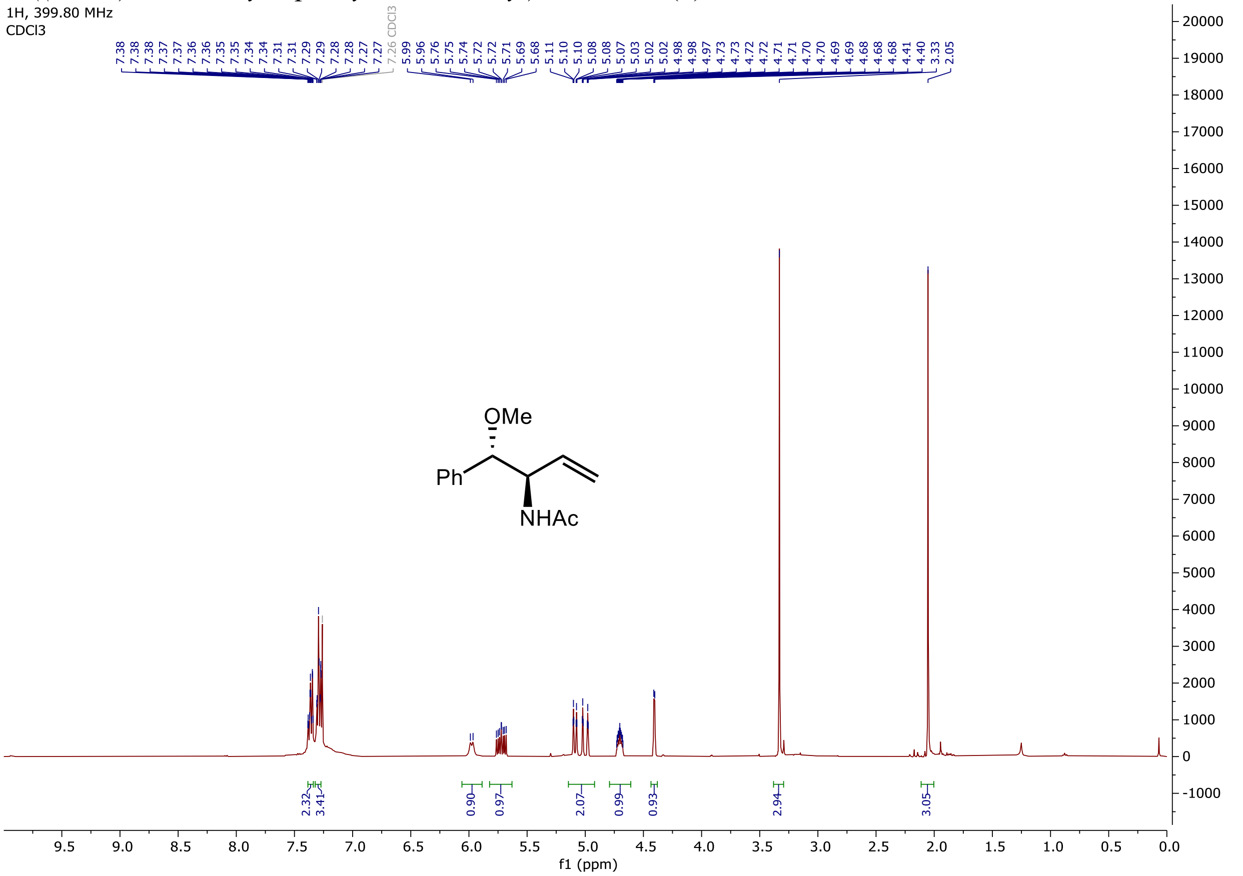

N-((±)-1-methoxy-1-phenylbut-3-en-2-yl)acetamide (±-**9**):

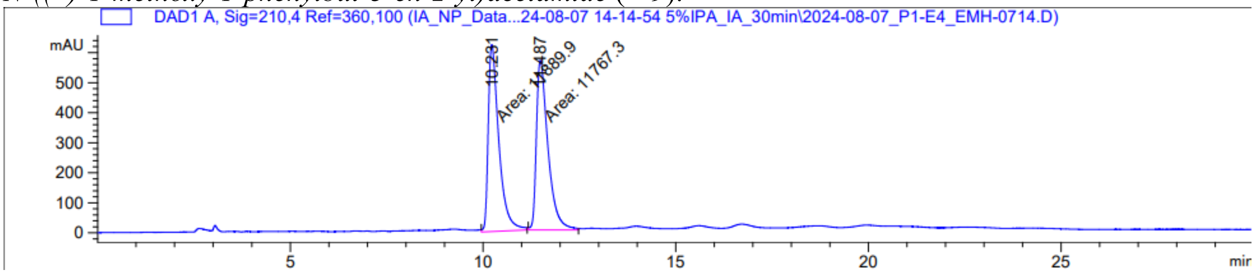

Peak #	RetTime [min]	Type	Width [min]	Area [mAU*s]	Height [mAU]	Area %
1	10.231	MM	0.3187	1.18899e4	621.84430	50.2591
2	11.487	MM	0.3473	1.17673e4	564.66492	49.7409

N-((1S,2R)-1-methoxy-1-phenylbut-3-en-2-yl)acetamide (**9**):

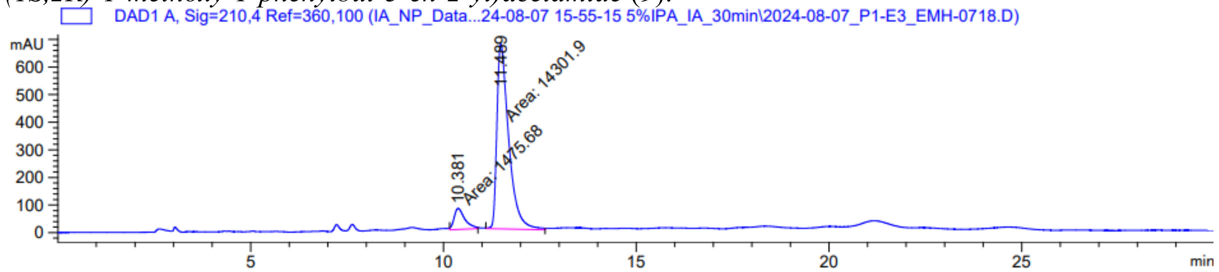

Peak #	RetTime [min]	Type	Width [min]	Area [mAU*s]	Height [mAU]	Area %
1	10.381	MM	0.3219	1475.67737	76.41270	9.3530
2	11.489	MM	0.3560	1.43019e4	669.60260	90.6470

N-((1S,2R)-1-(4-bromophenyl)-1-methoxybut-3-en-2-yl)acetamide (**10**):

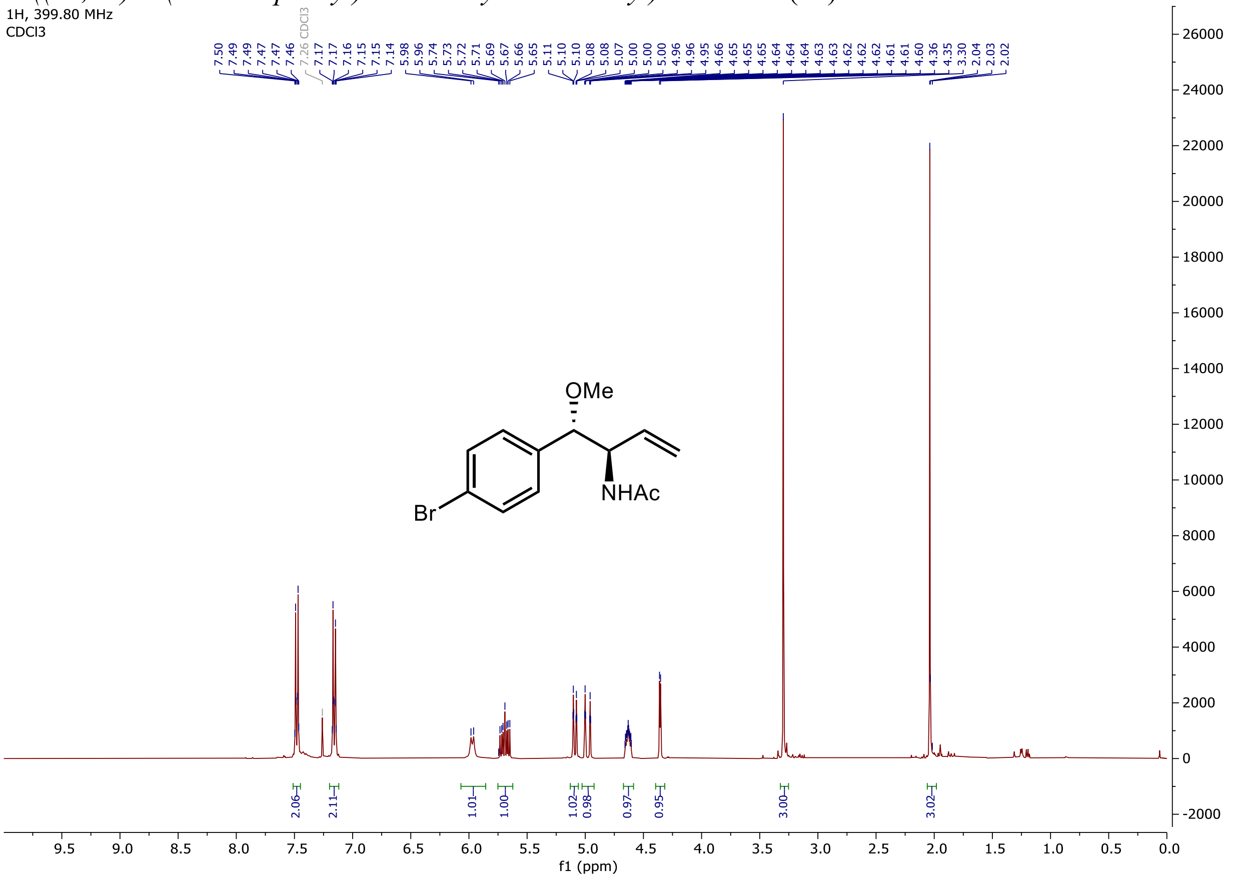

N-((±)-1-(4-bromophenyl)-1-methoxybut-3-en-2-yl)acetamide (±-**10**):

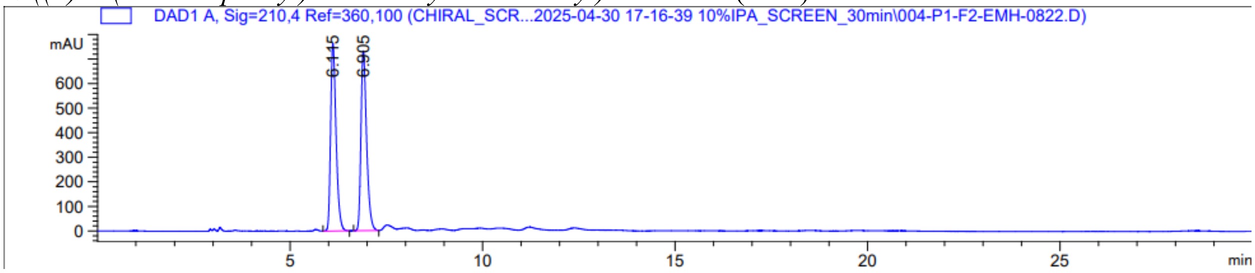

Peak #	RetTime [min]	Type	Width [min]	Area [mAU*s]	Height [mAU]	Area %
1	6.115	VV R	0.1236	7485.67139	762.37970	50.1664
2	6.905	VV R	0.1465	7436.00439	732.00635	49.8336

N-((1S,2R)-1-(4-bromophenyl)-1-methoxybut-3-en-2-yl)acetamide (**10**):

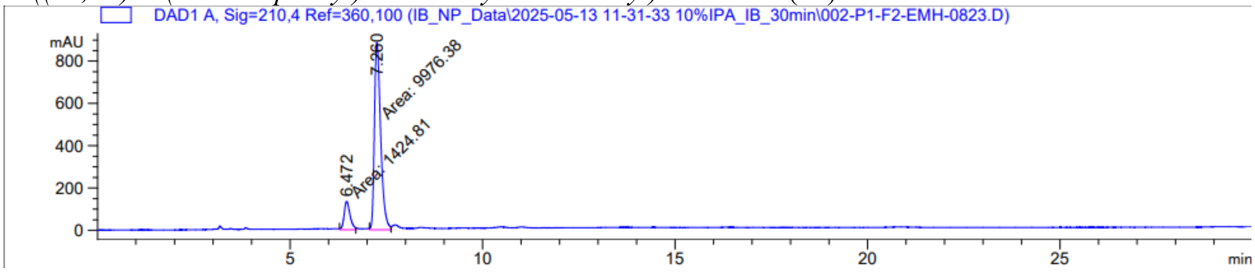

Peak #	RetTime [min]	Type	Width [min]	Area [mAU*s]	Height [mAU]	Area %
1	6.472	MM	0.1775	1424.80884	133.78577	12.4970
2	7.260	MM	0.1880	9976.37891	884.26947	87.5030

N-((S)-1-((R)-tetrahydrofuran-2-yl)allyl)acetamide (**11**):

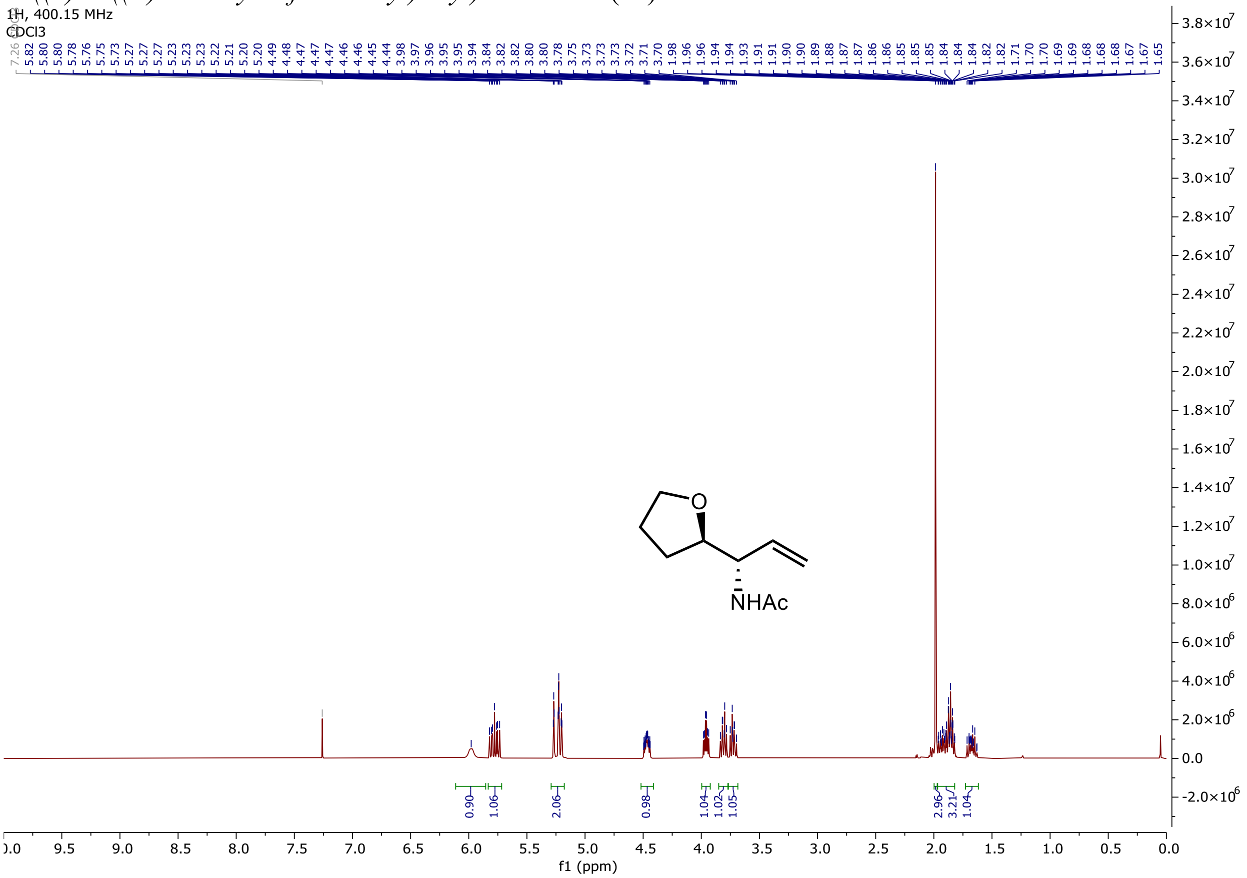

N-((±)-1-((±)-tetrahydrofuran-2-yl)allyl)acetamide (±-**11**):

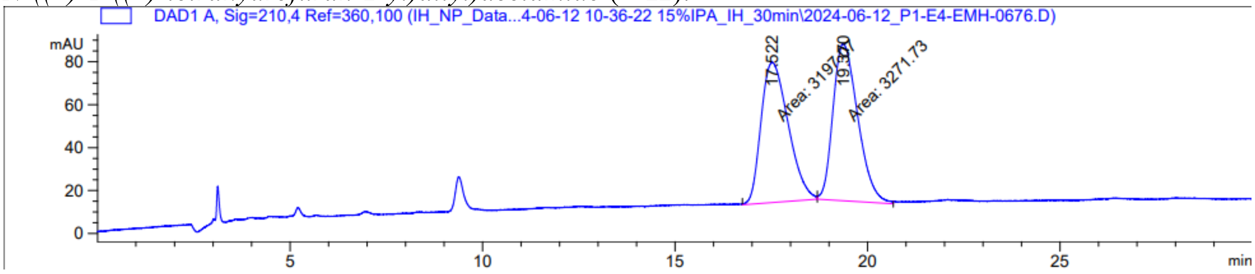

Peak #	RetTime [min]	Type	Width [min]	Area [mAU*s]	Height [mAU]	Area %
1	17.522	MM	0.8163	3197.06958	65.27864	49.4229
2	19.370	MM	0.7467	3271.72705	73.02818	50.5771

N-((S)-1-((R)-tetrahydrofuran-2-yl)allyl)acetamide (**11**):

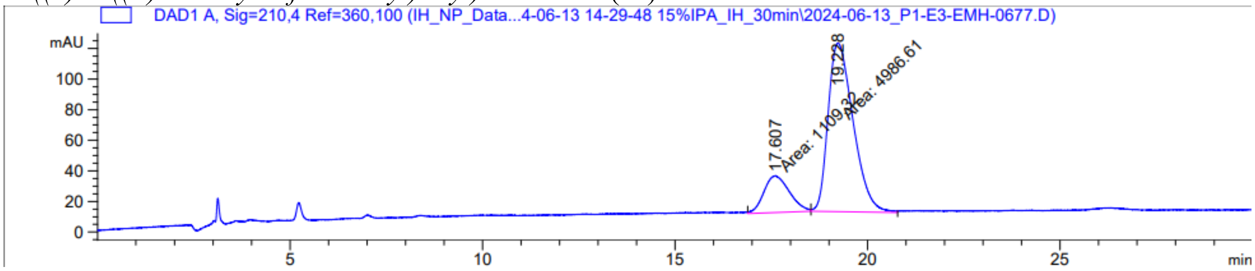

Peak #	RetTime [min]	Type	Width [min]	Area [mAU*s]	Height [mAU]	Area %
1	17.607	MM	0.7681	1109.32458	24.07012	18.1978
2	19.228	MM	0.7529	4986.61230	110.38737	81.8022

N-((S)-1-((S)-4-tosylmorpholin-3-yl)allyl)acetamide (**12**):

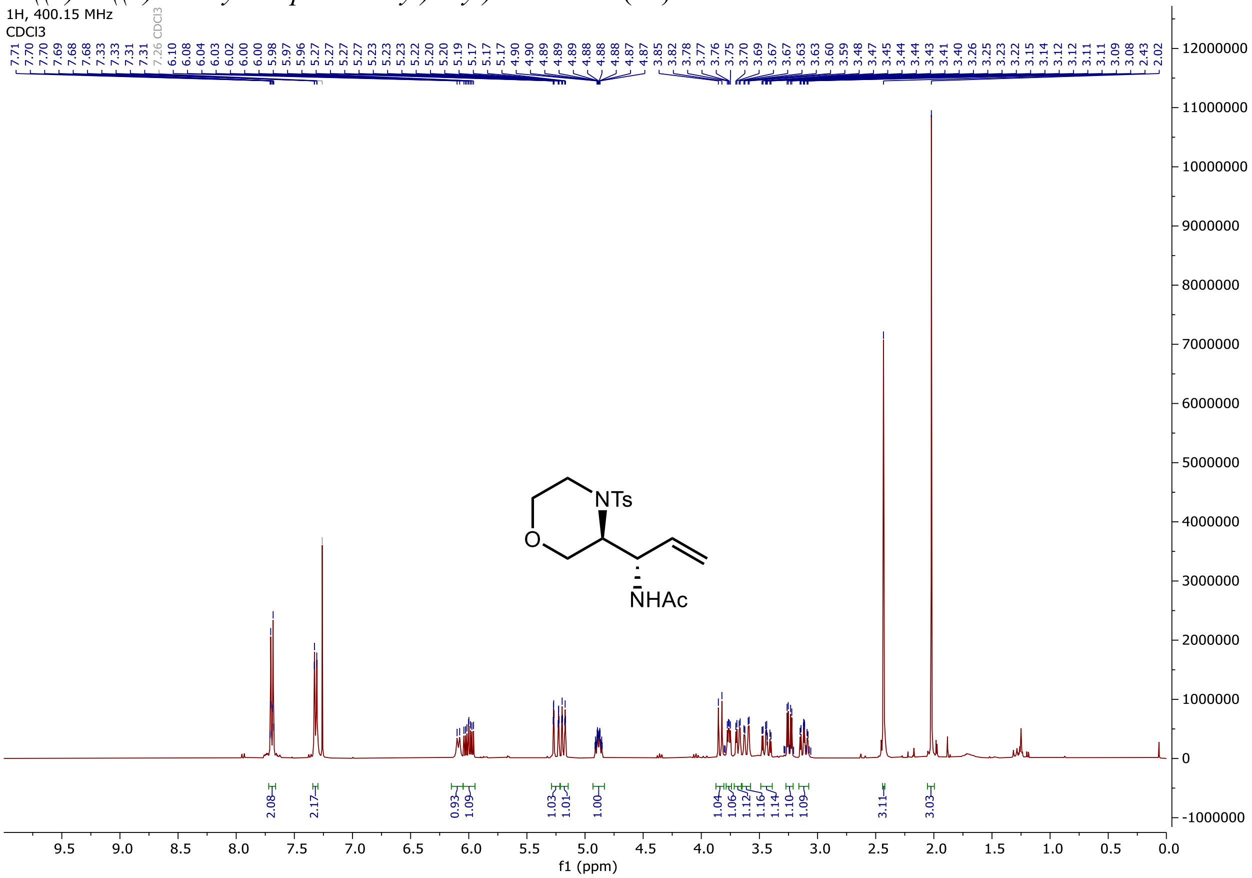

N-((±)-1-((±)-4-tosylmorpholin-3-yl)allyl)acetamide (±-**12**):

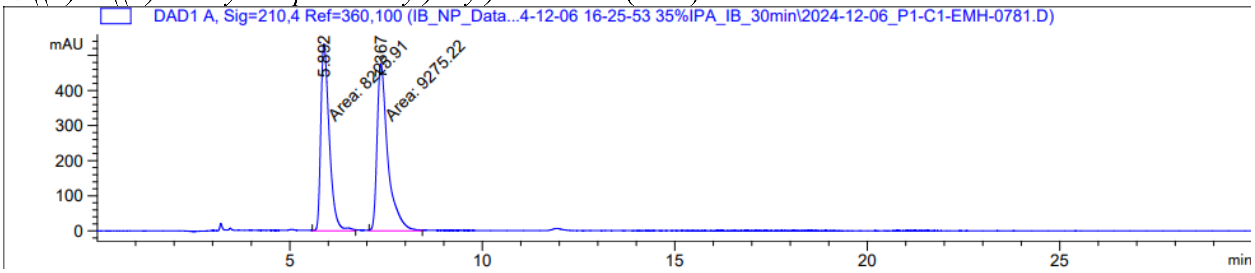

Peak #	RetTime [min]	Type	Width [min]	Area [mAU*s]	Height [mAU]	Area %
1	5.892	MM	0.2574	8225.91406	532.66852	47.0022
2	7.367	MM	0.3242	9275.22168	476.78091	52.9978

N-((*S*)-1-((*S*)-4-tosylmorpholin-3-yl)allyl)acetamide (**12**):

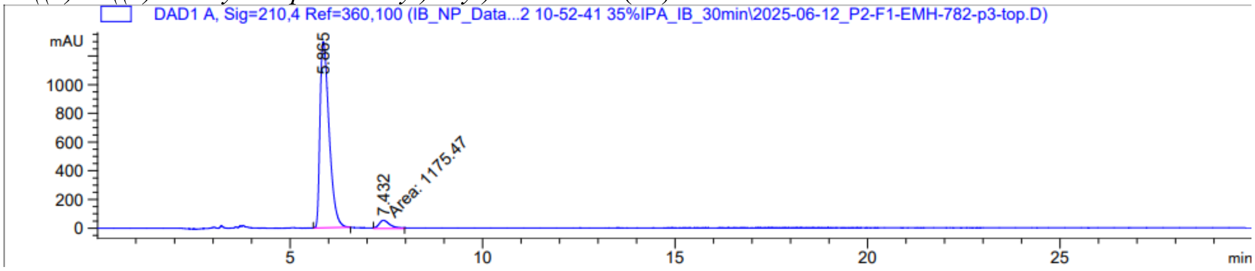

Peak #	RetTime [min]	Type	Width [min]	Area [mAU*s]	Height [mAU]	Area %
1	5.865	BV R	0.1939	2.12732e4	1296.79346	94.7638
2	7.432	MM	0.3398	1175.46729	57.65815	5.2362

Ethyl (4S,5R)-5-acetamido-4-(benzyloxy)hept-6-enoate (**14**):

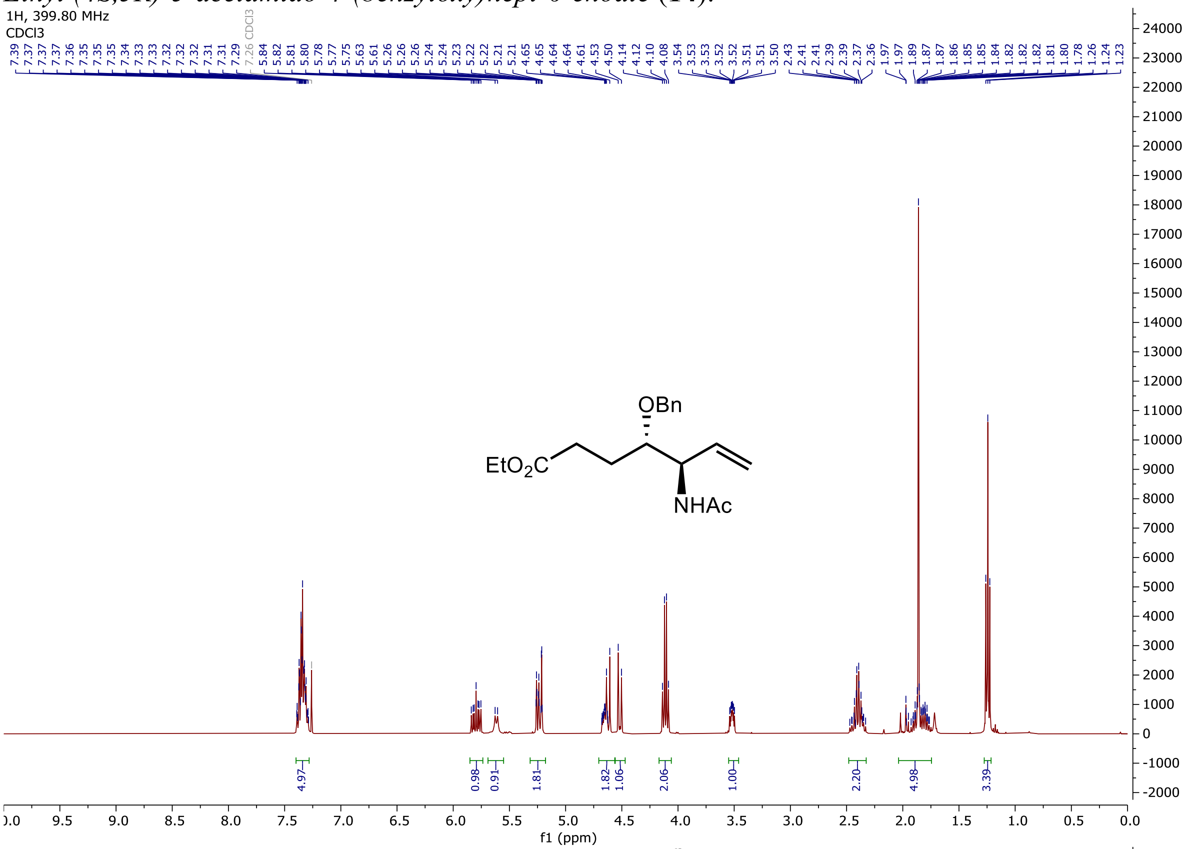

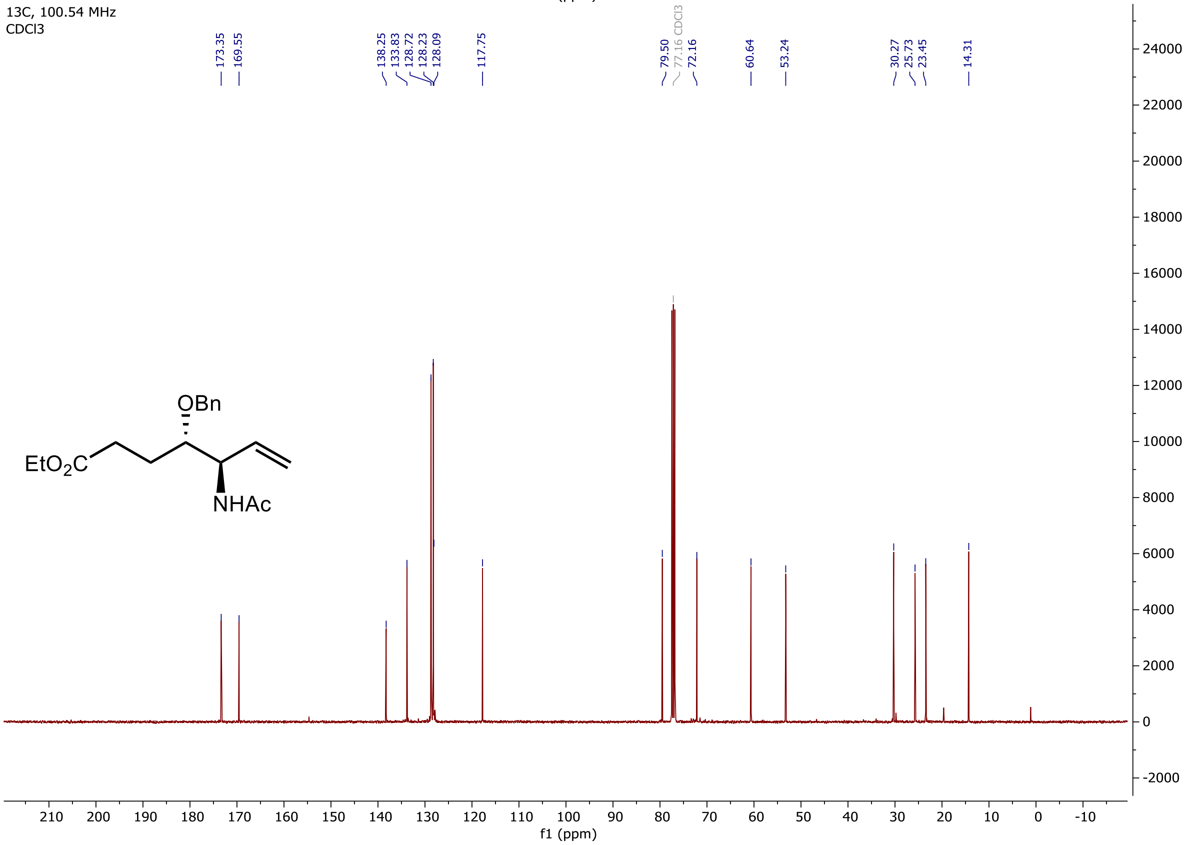

Ethyl (±)-5-acetamido-4-(benzyloxy)hept-6-enoate (±-**14**):

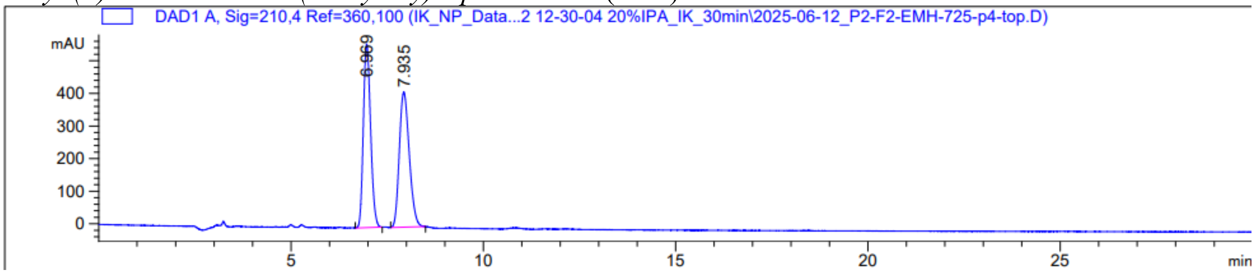

Peak #	RetTime [min]	Type	Width [min]	Area [mAU*s]	Height [mAU]	Area %
1	6.969	VV R	0.1695	7352.72266	562.66644	49.6506
2	7.935	BV R	0.2120	7456.20264	414.23038	50.3494

Ethyl (4S,5R)-5-acetamido-4-(benzyloxy)hept-6-enoate (**14**):

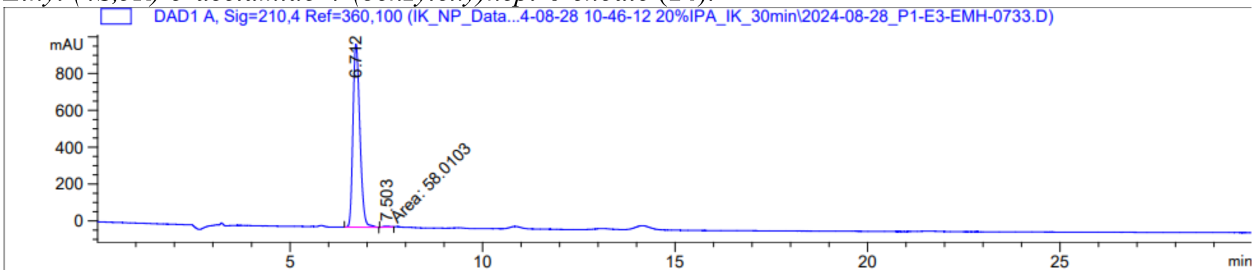

Peak #	RetTime [min]	Type	Width [min]	Area [mAU*s]	Height [mAU]	Area %
1	6.712	VV R	0.1514	1.23838e4	993.34058	99.5337
2	7.503	MM	0.1859	58.01028	5.20193	0.4663

Ethyl (4S,5R)-5-acetamido-4-(cyclopentyloxy)hept-6-enoate (**15**):

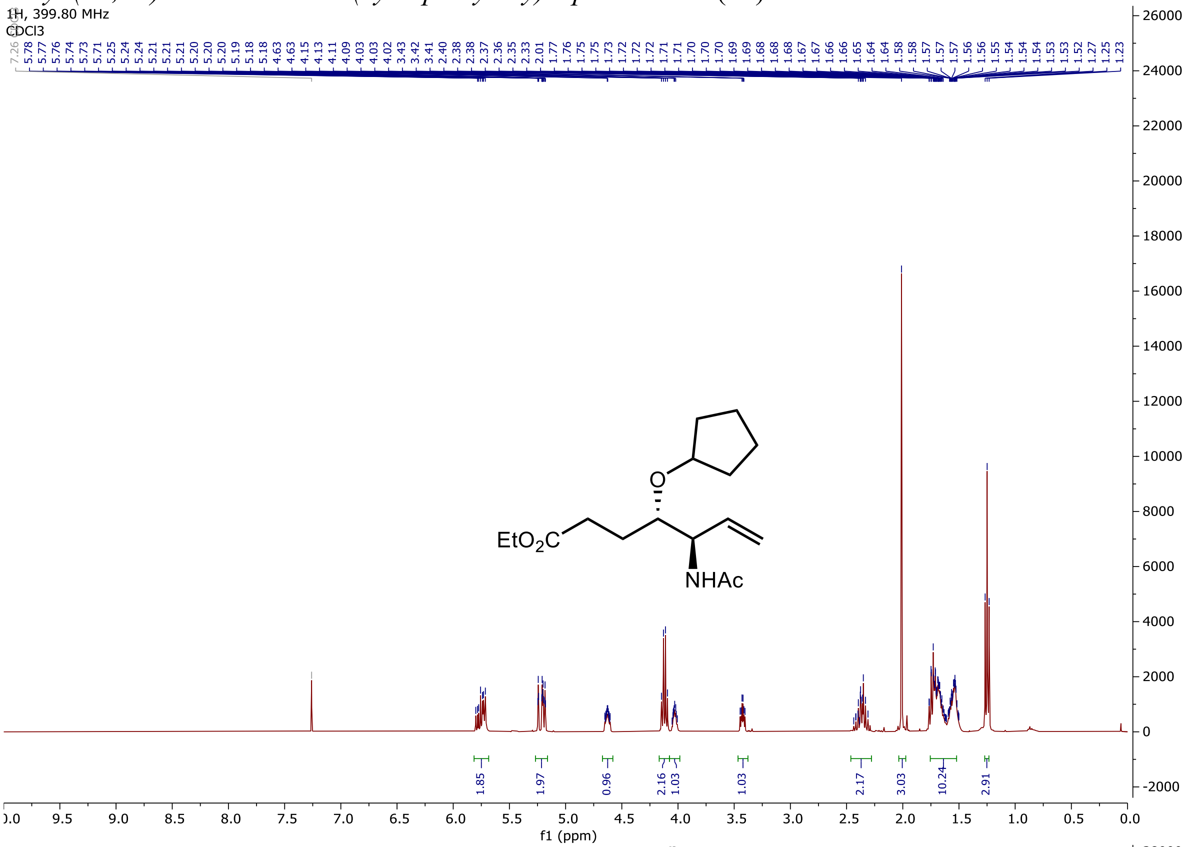

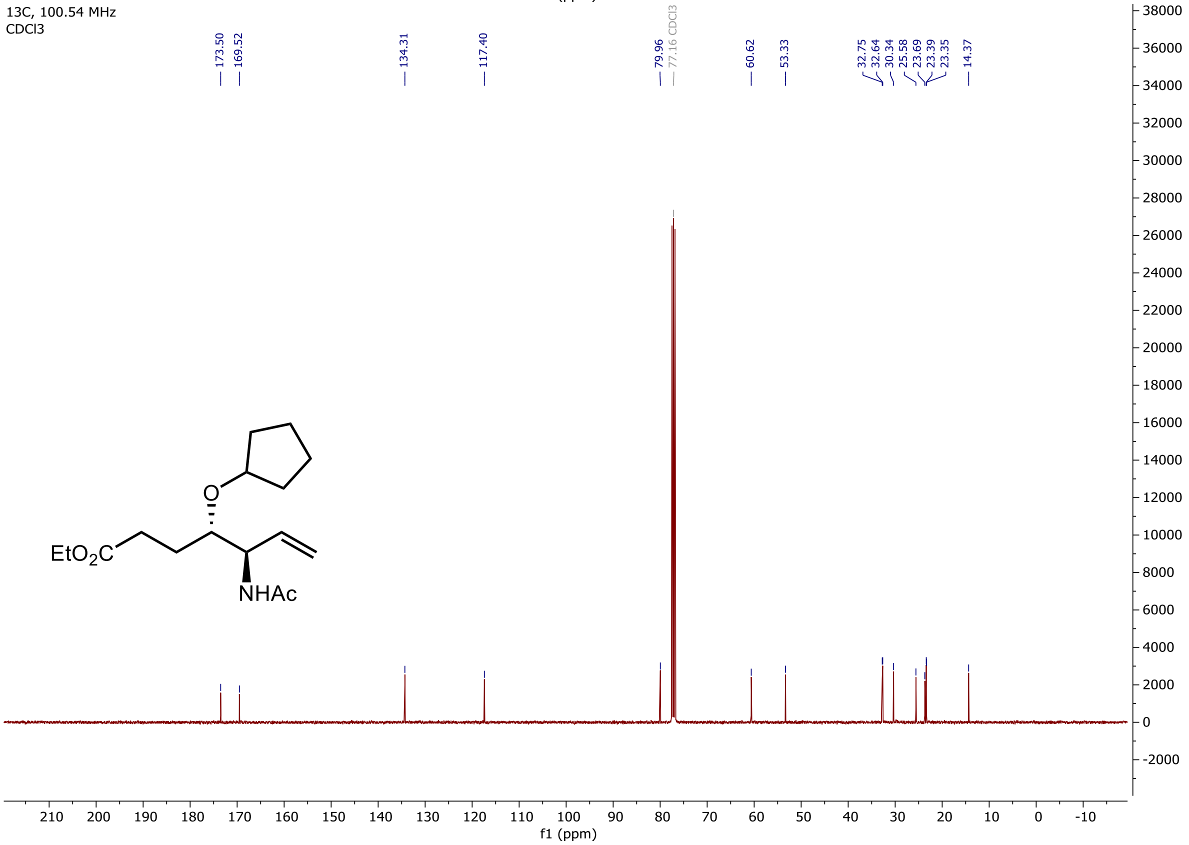

Ethyl (±)-5-acetamido-4-(cyclopentyloxy)hept-6-enoate (±-**15**):

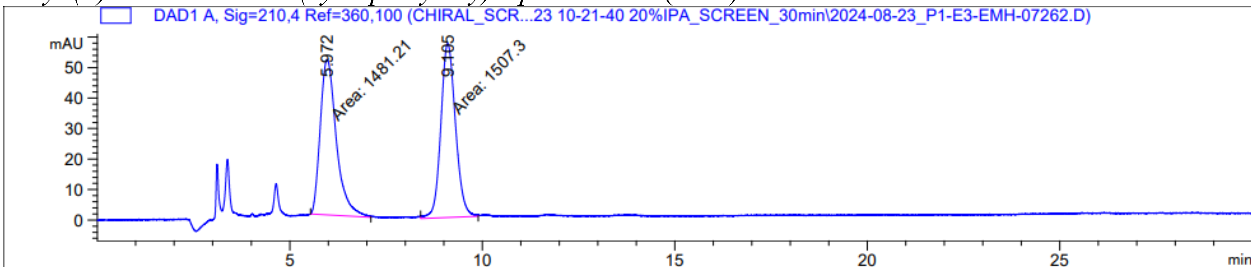

Peak #	RetTime [min]	Type	Width [min]	Area [mAU*s]	Height [mAU]	Area %
1	5.972	MM	0.4838	1481.20605	51.03013	49.5634
2	9.105	MM	0.4405	1507.29895	57.02596	50.4366

Ethyl (4S,5R)-5-acetamido-4-(cyclopentyloxy)hept-6-enoate (**15**):

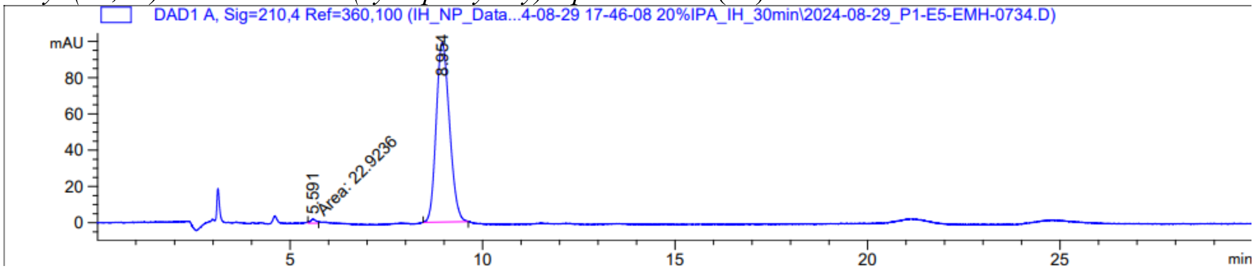

Peak #	RetTime [min]	Type	Width [min]	Area [mAU*s]	Height [mAU]	Area %
1	5.591	MM	0.1665	22.92362	2.29400	0.9542
2	8.954	VV R	0.2843	2379.53613	98.87262	99.0458

Ethyl (4R,5S)-5-acetamido-4-(furan-2-ylmethoxy)hept-6-enoate (**16**):

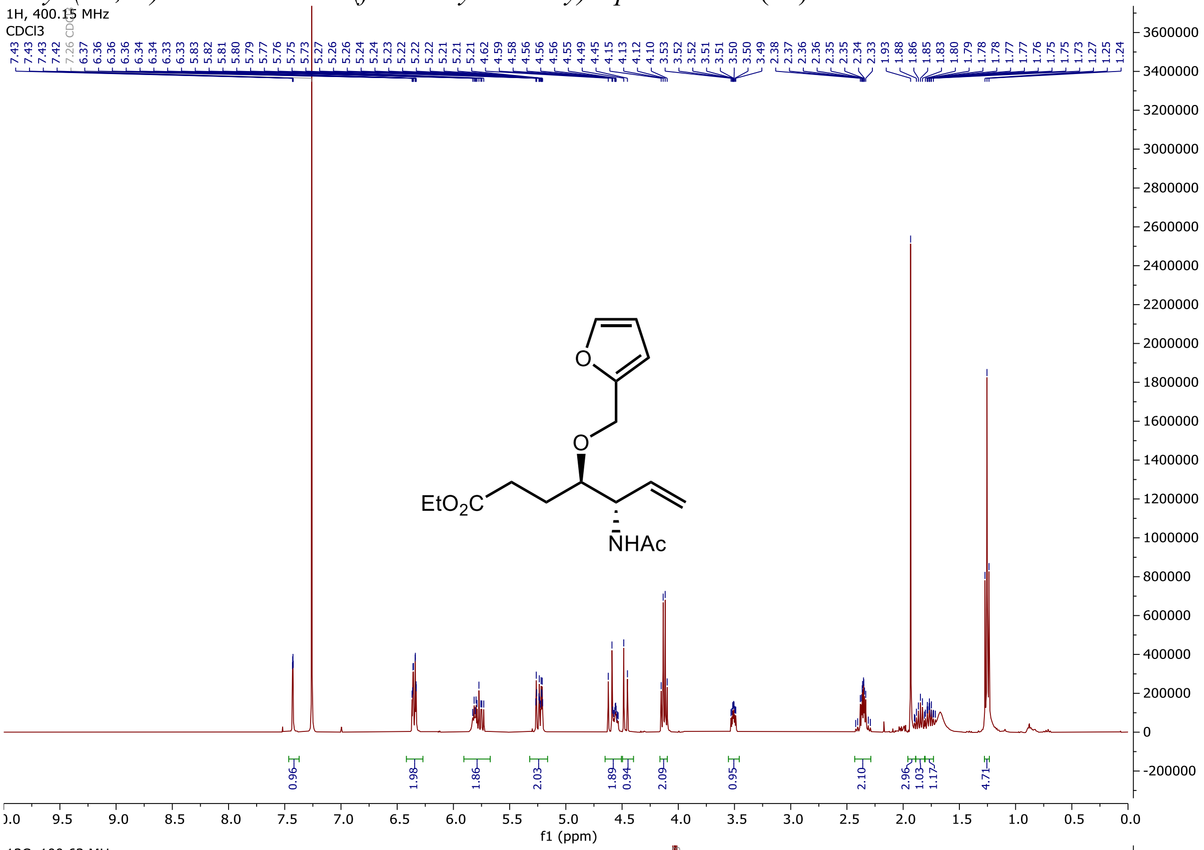

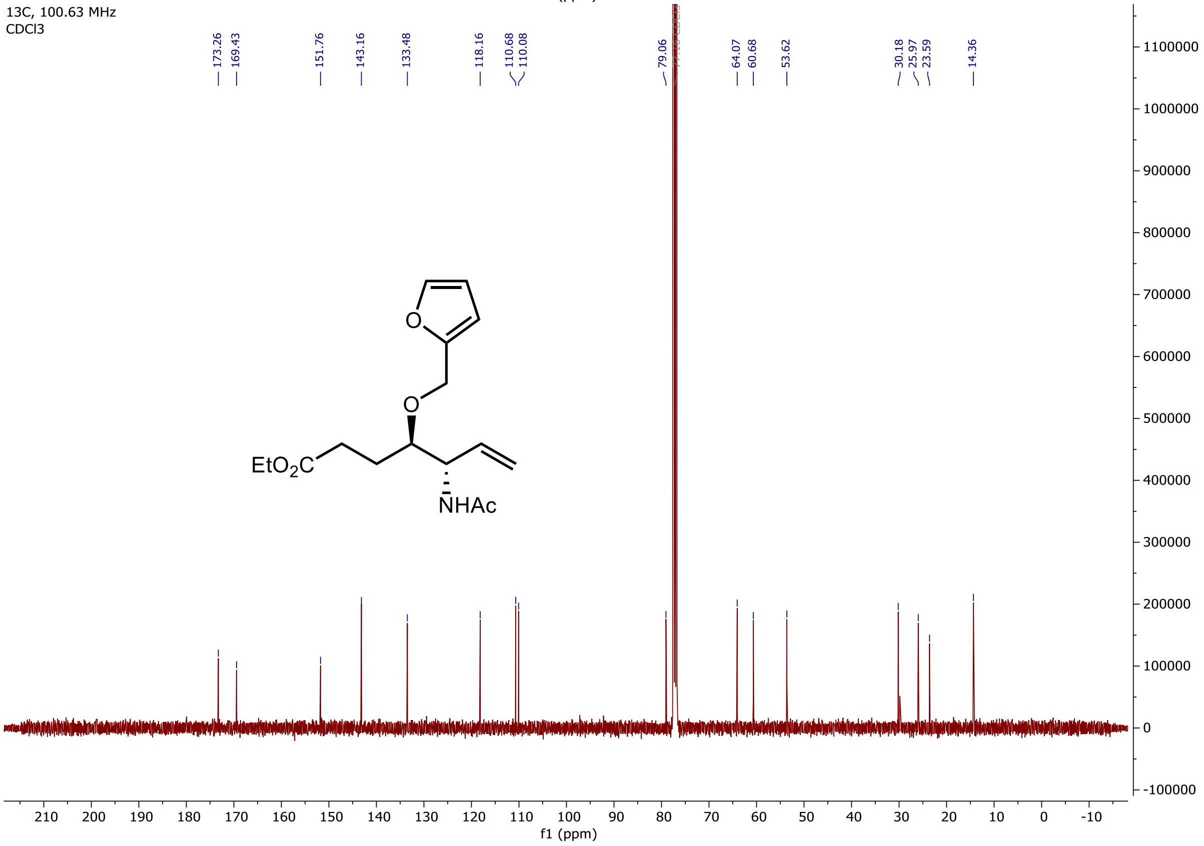

Ethyl (±)-5-acetamido-4-(furan-2-ylmethoxy)hept-6-enoate (±-**16**):

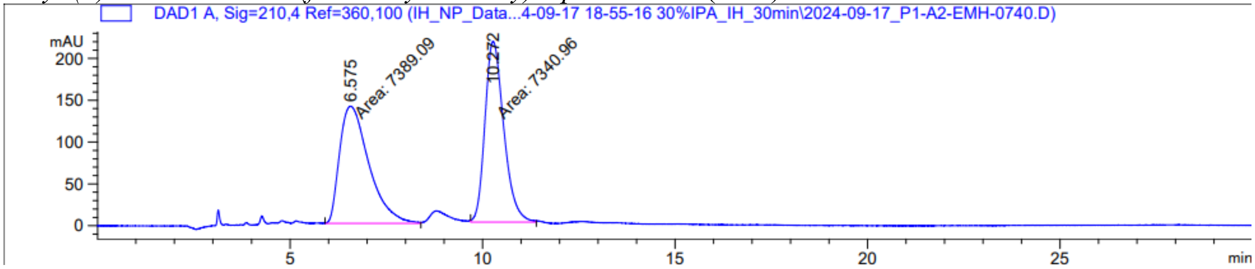

Peak #	RetTime [min]	Type	Width [min]	Area [mAU*s]	Height [mAU]	Area %
1	6.575	MM	0.8801	7389.08887	139.93521	50.1634
2	10.272	MM	0.5658	7340.95557	216.22455	49.8366

Ethyl (4R,5S)-5-acetamido-4-(furan-2-ylmethoxy)hept-6-enoate (**16**):

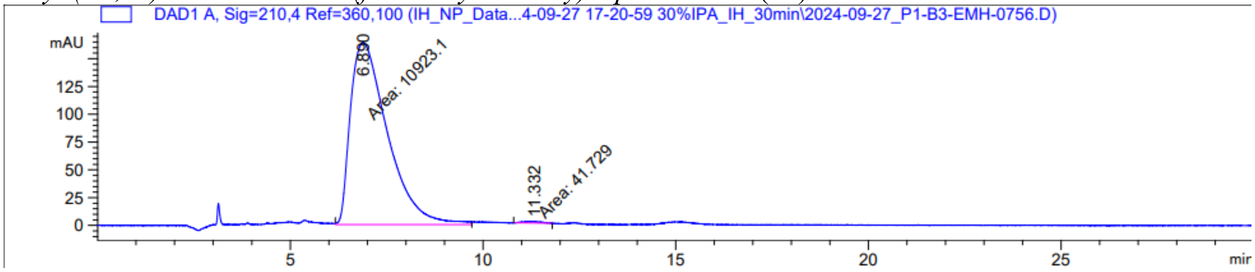

Peak #	RetTime [min]	Type	Width [min]	Area [mAU*s]	Height [mAU]	Area %
1	6.890	MM	1.1113	1.09231e4	163.82285	99.6194
2	11.332	MM	0.4935	41.72901	1.40932	0.3806

Ethyl (4R,5S)-5-acetamido-4-(thiophen-3-ylmethoxy)hept-6-enoate (**17**):

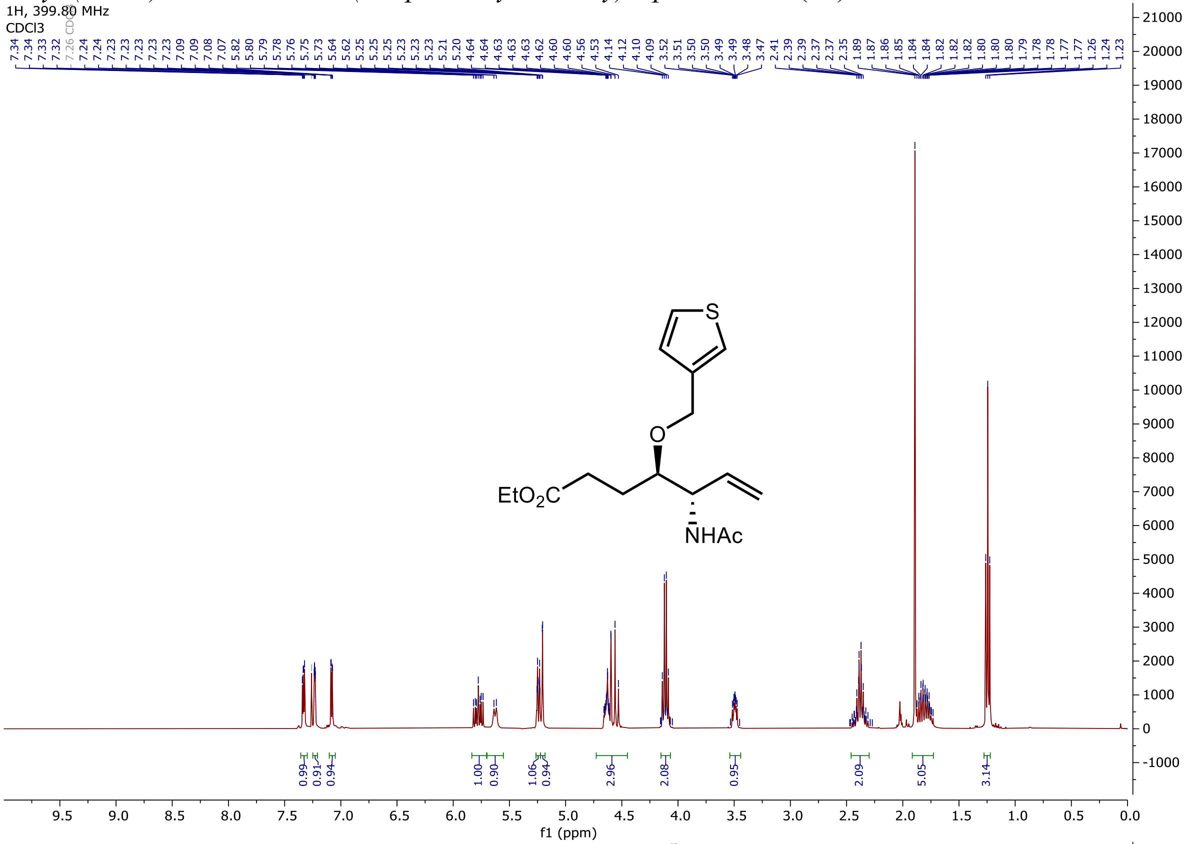

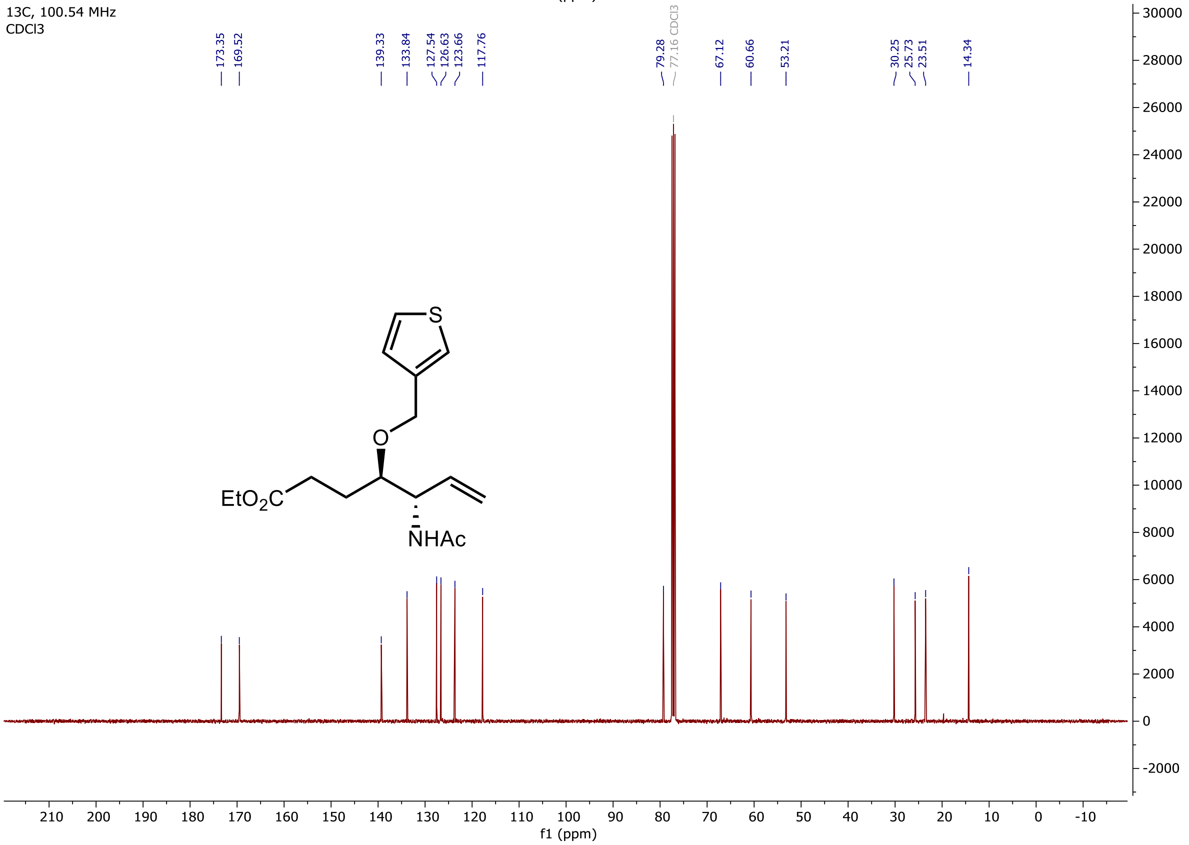

Ethyl (±)-5-acetamido-4-(thiophen-3-ylmethoxy)hept-6-enoate (±-**17**):

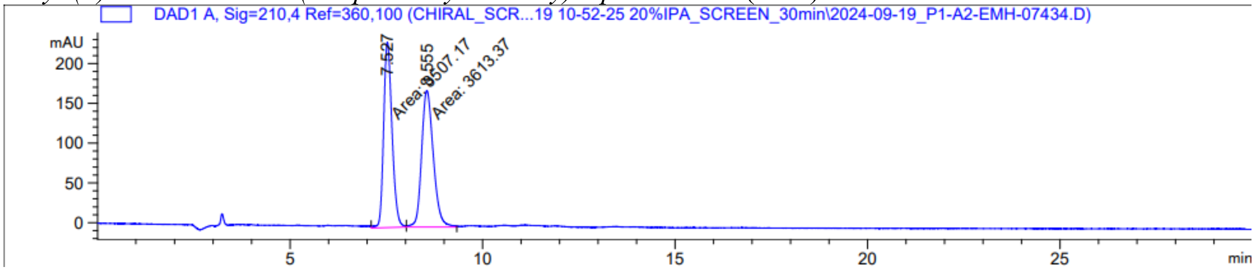

Peak #	RetTime [min]	Type	Width [min]	Area [mAU*s]	Height [mAU]	Area %
1	7.527	MM	0.2510	3507.16943	232.86288	49.2543
2	8.555	MM	0.3515	3613.37207	171.34924	50.7457

Ethyl (4R,5S)-5-acetamido-4-(thiophen-3-ylmethoxy)hept-6-enoate (**17**):

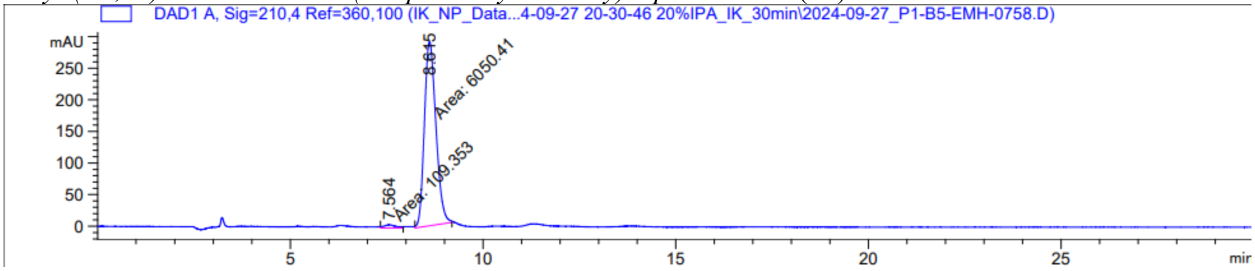

Peak #	RetTime [min]	Type	Width [min]	Area [mAU*s]	Height [mAU]	Area %
1	7.564	MM	0.3537	109.35323	5.15284	1.7753
2	8.615	MM	0.3463	6050.40723	291.18146	98.2247

tert-*Butyl 3-((((3S,4R)-3-acetamido-7-ethoxy-7-oxohept-1-en-4-yl)oxy)methyl)azetidine-1-carboxylate* (**18**):

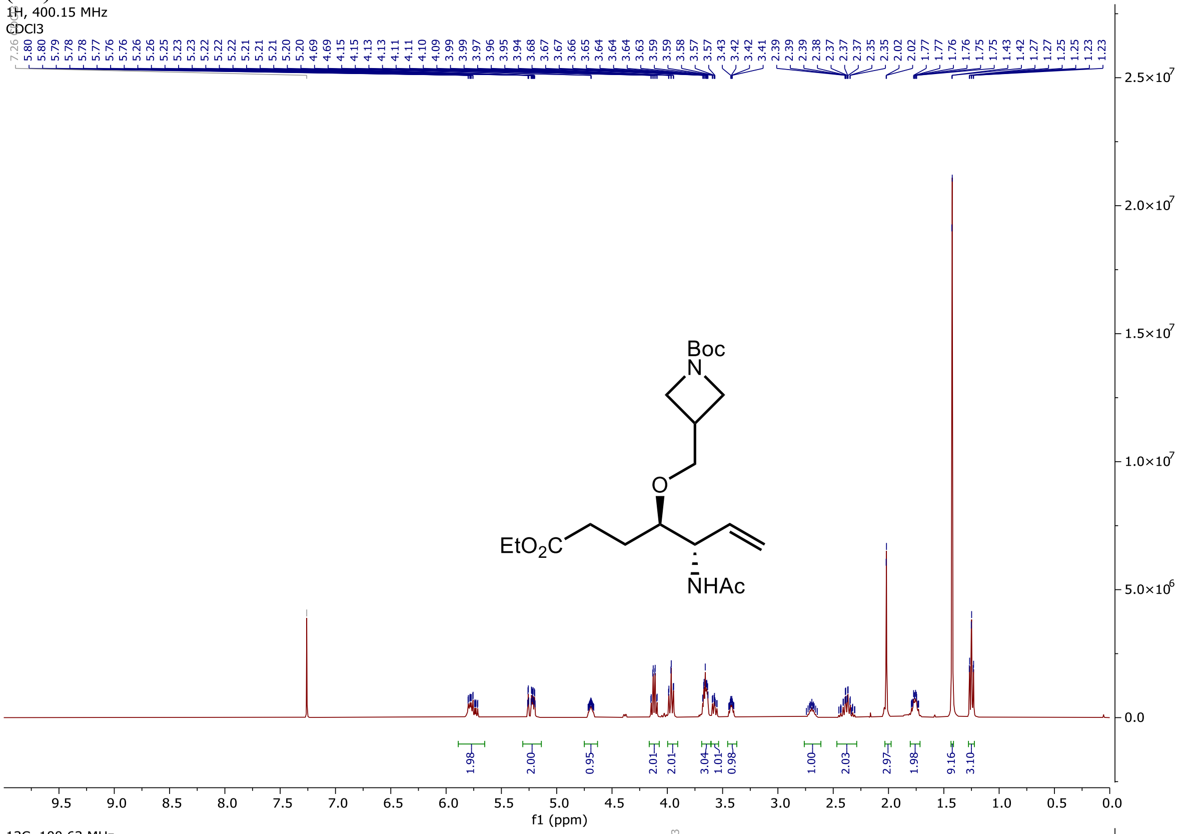

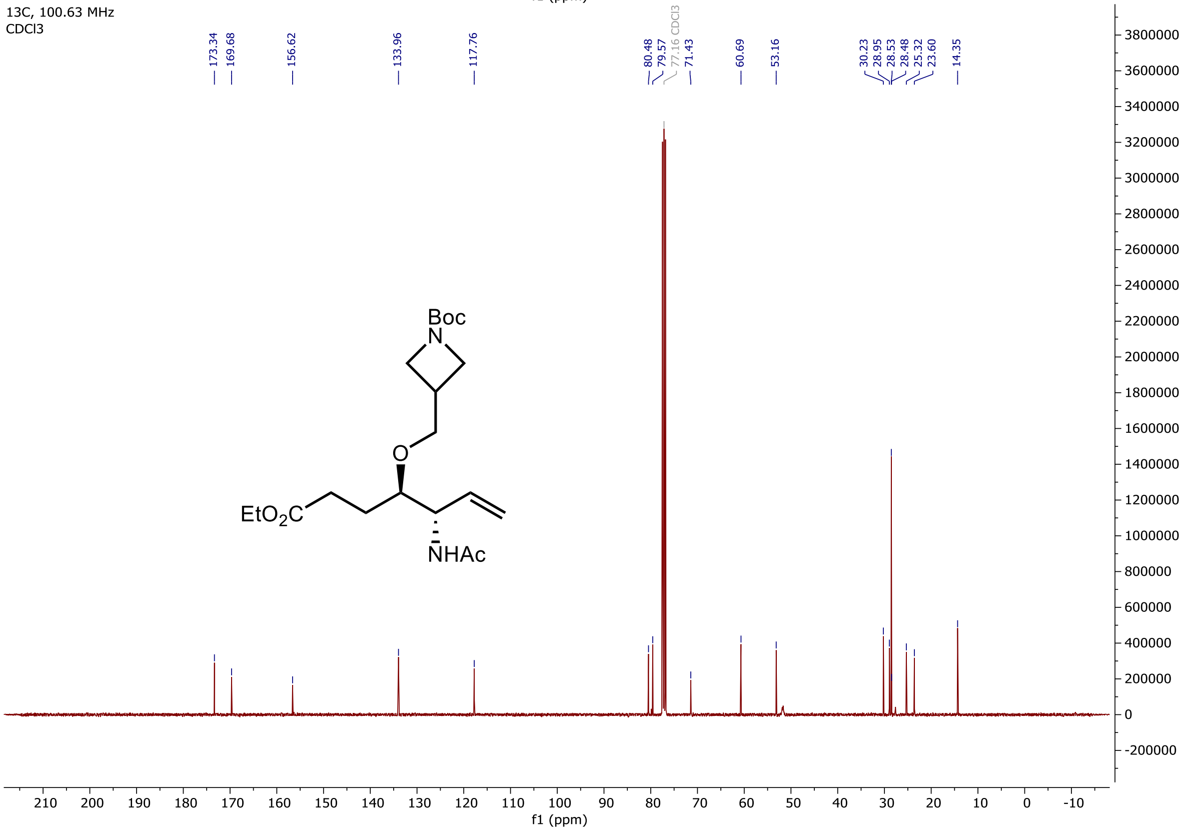

tert-*Butyl 3-((((±)-3-acetamido-7-ethoxy-7-oxohept-1-en-4-yl)oxy)methyl)azetidine-1-carboxylate* (±-**18**):

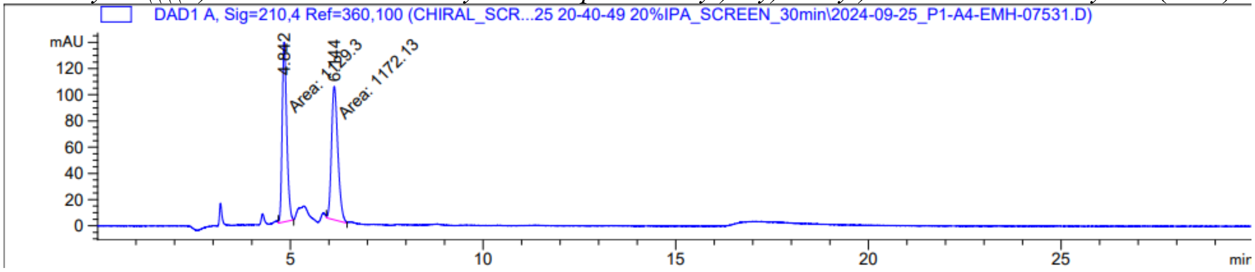

Peak #	RetTime [min]	Type	Width [min]	Area [mAU*s]	Height [mAU]	Area %
1	4.842	MM	0.1372	1129.30103	137.18788	49.0696
2	6.144	MM	0.1920	1172.12695	101.74653	50.9304

tert-*Butyl 3-((((3S,4R)-3-acetamido-7-ethoxy-7-oxohept-1-en-4-yl)oxy)methyl)azetidine-1-carboxylate* (**18**):

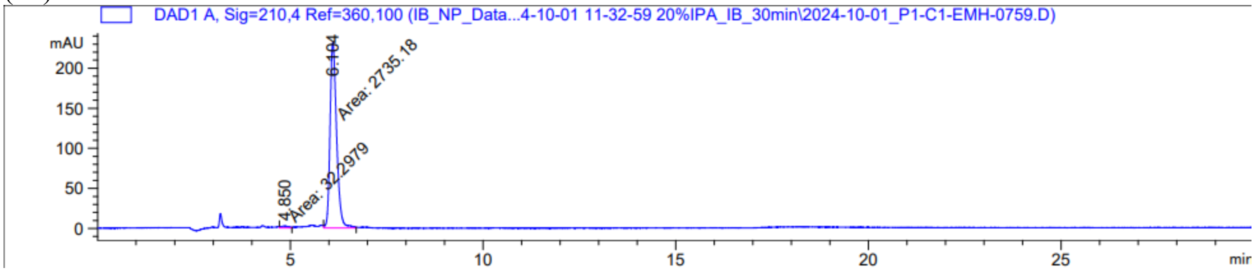

Peak #	RetTime [min]	Type	Width [min]	Area [mAU*s]	Height [mAU]	Area %
1	4.850	MM	0.2124	32.29794	2.53409	1.1671
2	6.104	MM	0.1973	2735.17822	231.00534	98.8329

Ethyl (4S,5R)-5-benzamido-4-methoxyhept-6-enoate (**19**):

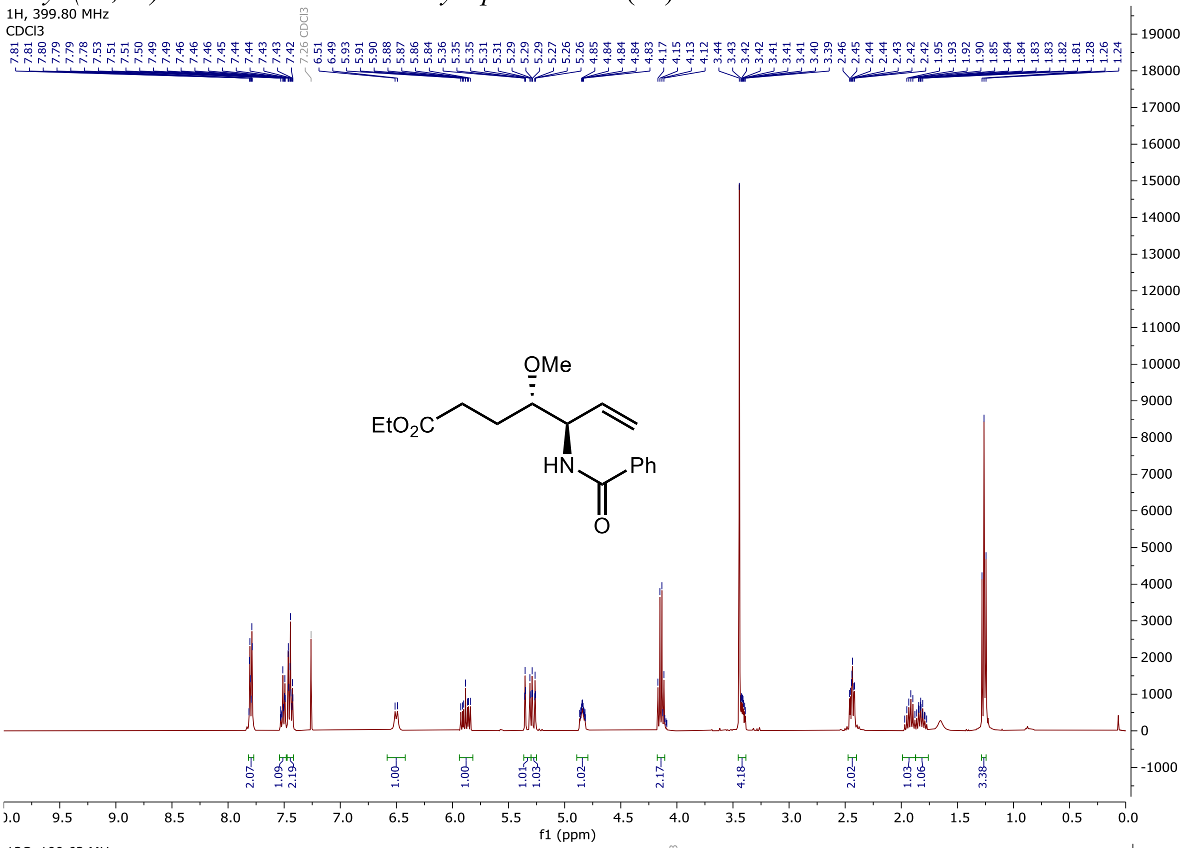

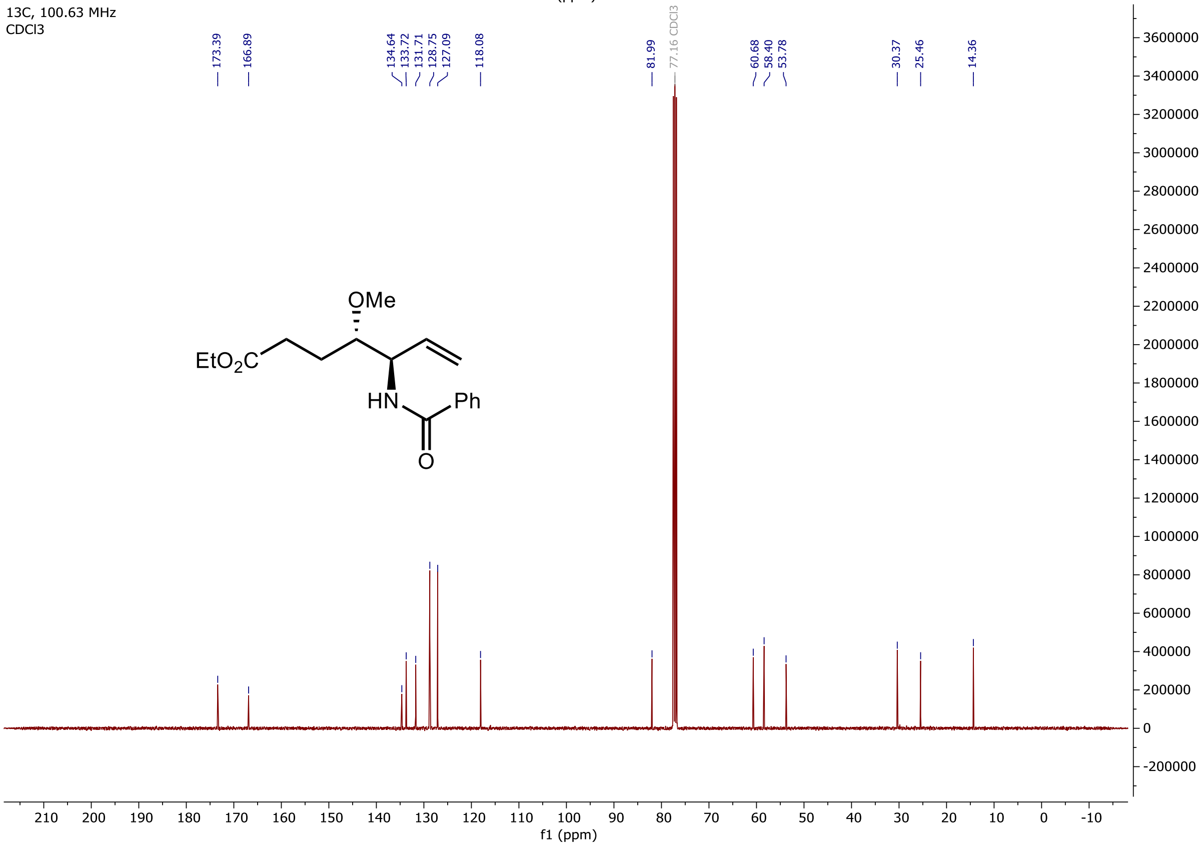

Ethyl (±)-5-benzamido-4-methoxyhept-6-enoate (±-**19**):

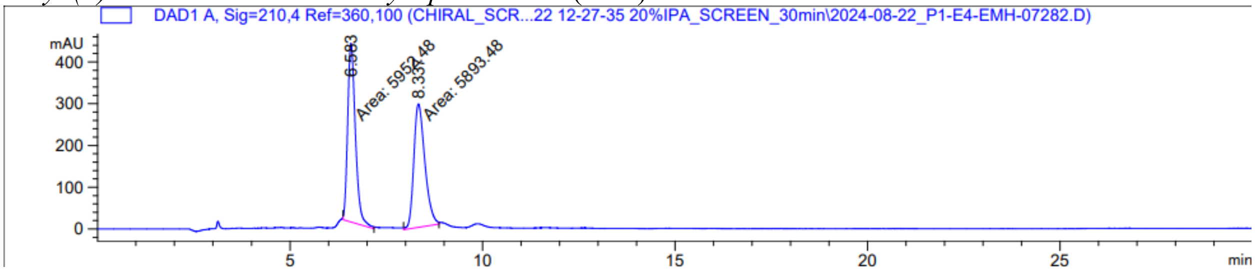

Peak #	RetTime [min]	Type	Width [min]	Area [mAU*s]	Height [mAU]	Area %
1	6.583	MM	0.2319	5952.48193	427.76956	50.2491
2	8.337	MM	0.3324	5893.47510	295.51959	49.7509

Ethyl (4S,5R)-5-benzamido-4-methoxyhept-6-enoate (**19**):

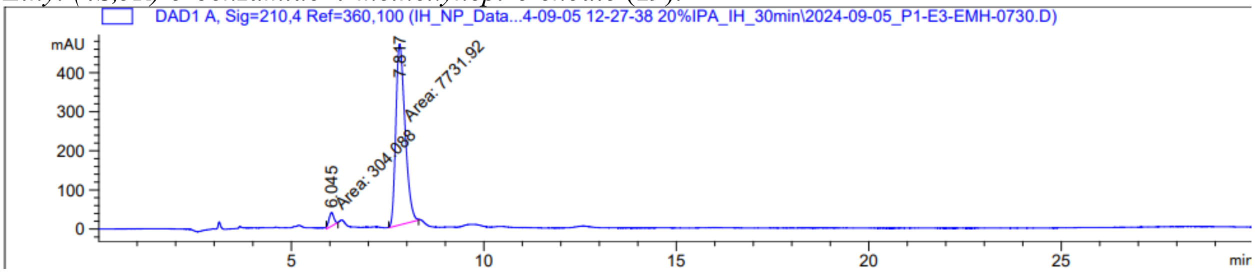

Peak #	RetTime [min]	Type	Width [min]	Area [mAU*s]	Height [mAU]	Area %
1	6.045	MM	0.1456	304.08832	34.82000	3.7841
2	7.817	MM	0.2787	7731.92334	462.43140	96.2159

Ethyl (4R,5S)-4-methoxy-5-propionamidohept-6-enoate (**20**):

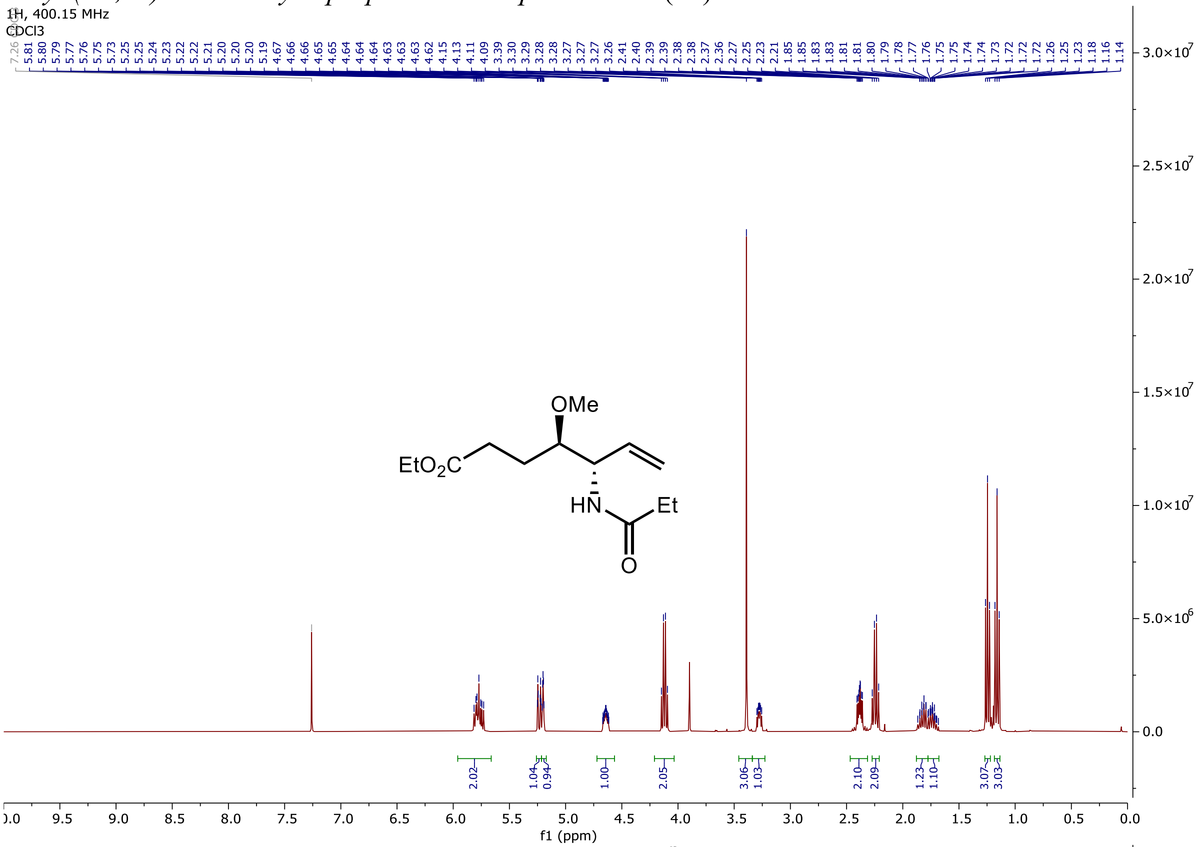

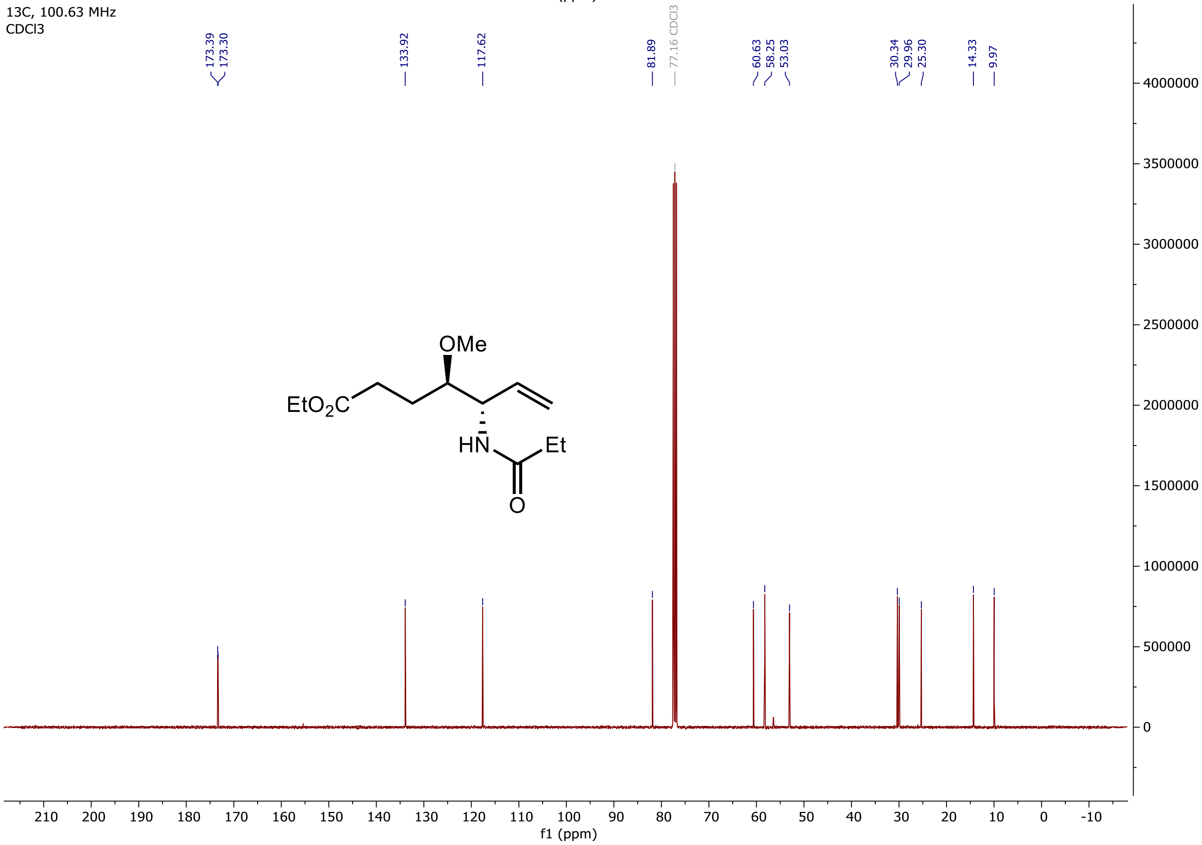

Ethyl (±)-4-methoxy-5-propionamidohept-6-enoate (±-**20**):

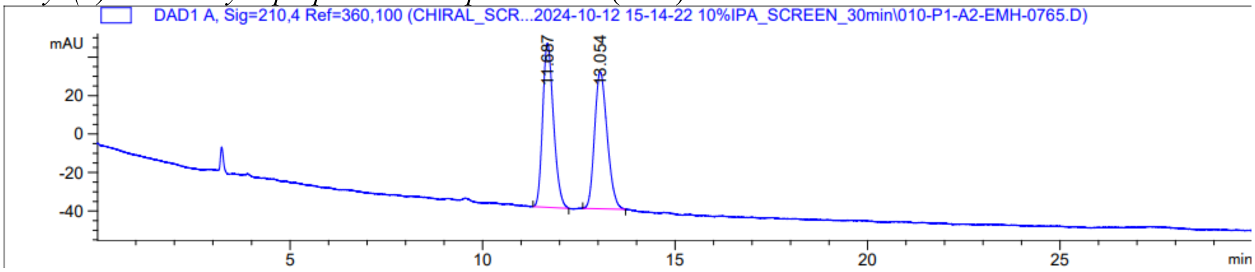

Peak #	RetTime [min]	Type	Width [min]	Area [mAU*s]	Height [mAU]	Area %
1	11.687	VV R	0.2331	1672.08948	85.31705	50.7407
2	13.054	VV R	0.2686	1623.27087	71.30059	49.2593

Ethyl (4R,5S)-4-methoxy-5-propionamidohept-6-enoate (**20**):

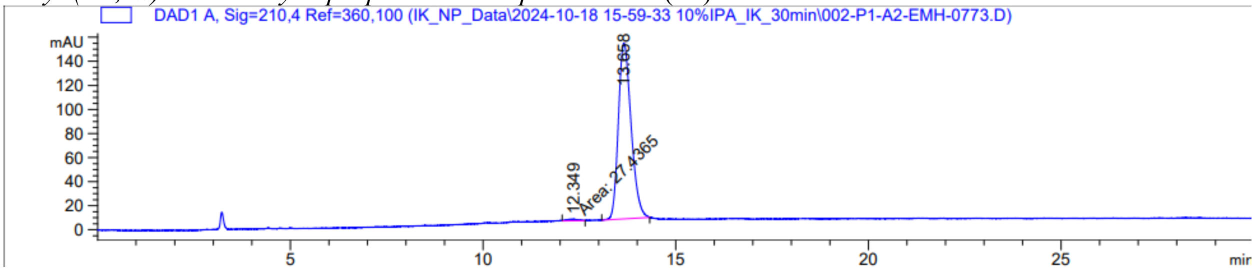

Peak #	RetTime [min]	Type	Width [min]	Area [mAU*s]	Height [mAU]	Area %
1	12.349	MM	0.3141	27.43649	1.45575	0.8126
2	13.658	VV R	0.2687	3348.95825	146.24956	99.1874

Ethyl (4R,5S)-5-isobutyramido-4-methoxyhept-6-enoate (**21**):

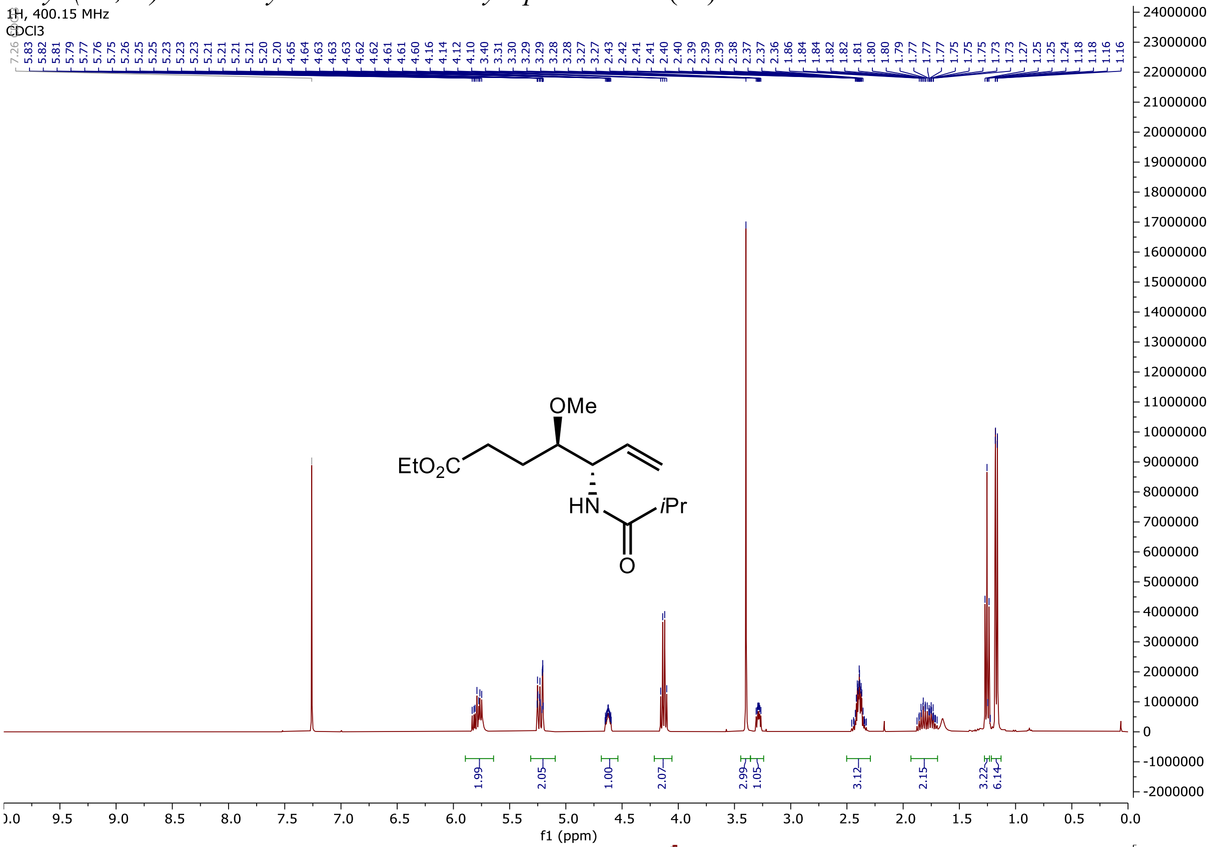

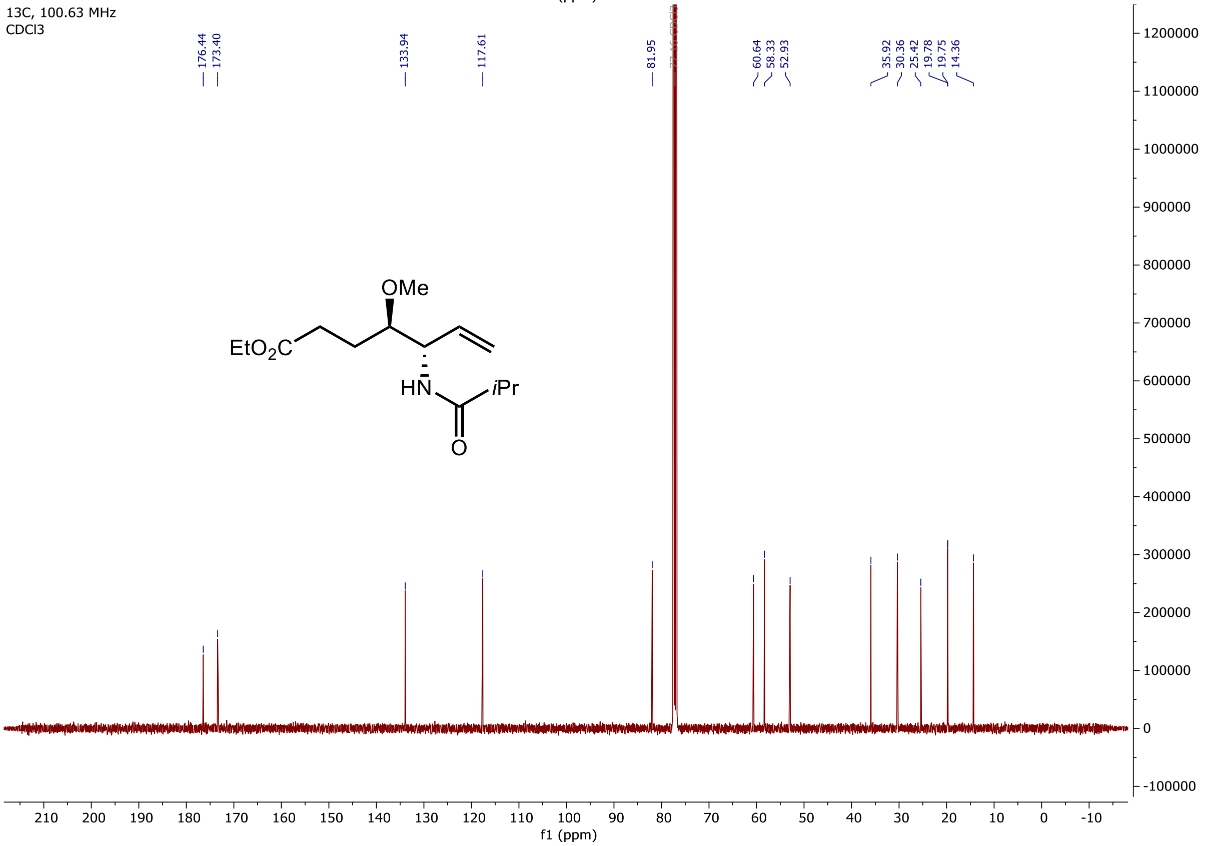

Ethyl (±)-5-isobutyramido-4-methoxyhept-6-enoate (±-**21**):

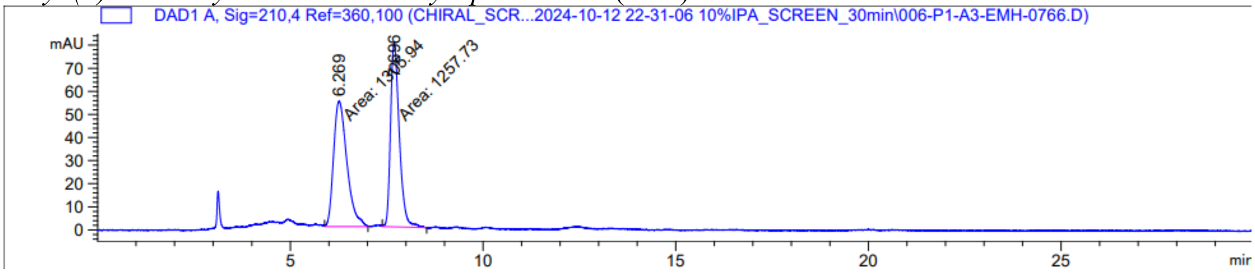

Peak #	RetTime [min]	Type	Width [min]	Area [mAU*s]	Height [mAU]	Area %
1	6.269	MM	0.4006	1305.94458	54.33277	50.9404
2	7.696	MM	0.2637	1257.72546	79.49033	49.0596

Ethyl (4R,5S)-5-isobutyramido-4-methoxyhept-6-enoate (**21**):

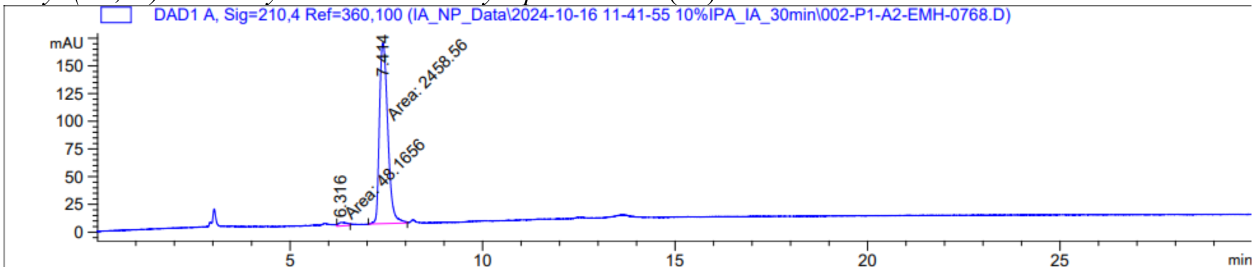

Peak #	RetTime [min]	Type	Width [min]	Area [mAU*s]	Height [mAU]	Area %
1	6.316	MM	0.2459	48.16561	3.26431	1.9215
2	7.414	MM	0.2511	2458.56152	163.15474	98.0785

Ethyl (4R,5S)-4-methoxy-5-pivalamidohept-6-enoate (**22**):

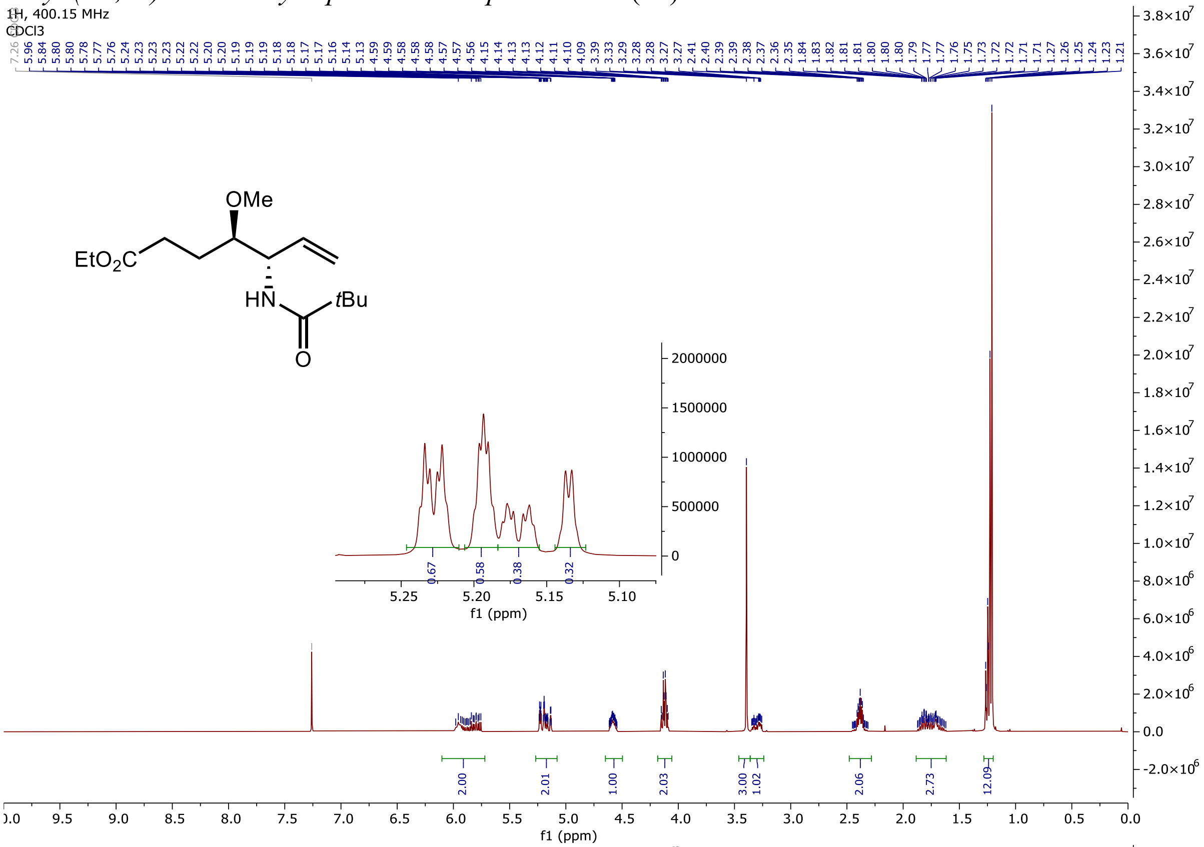

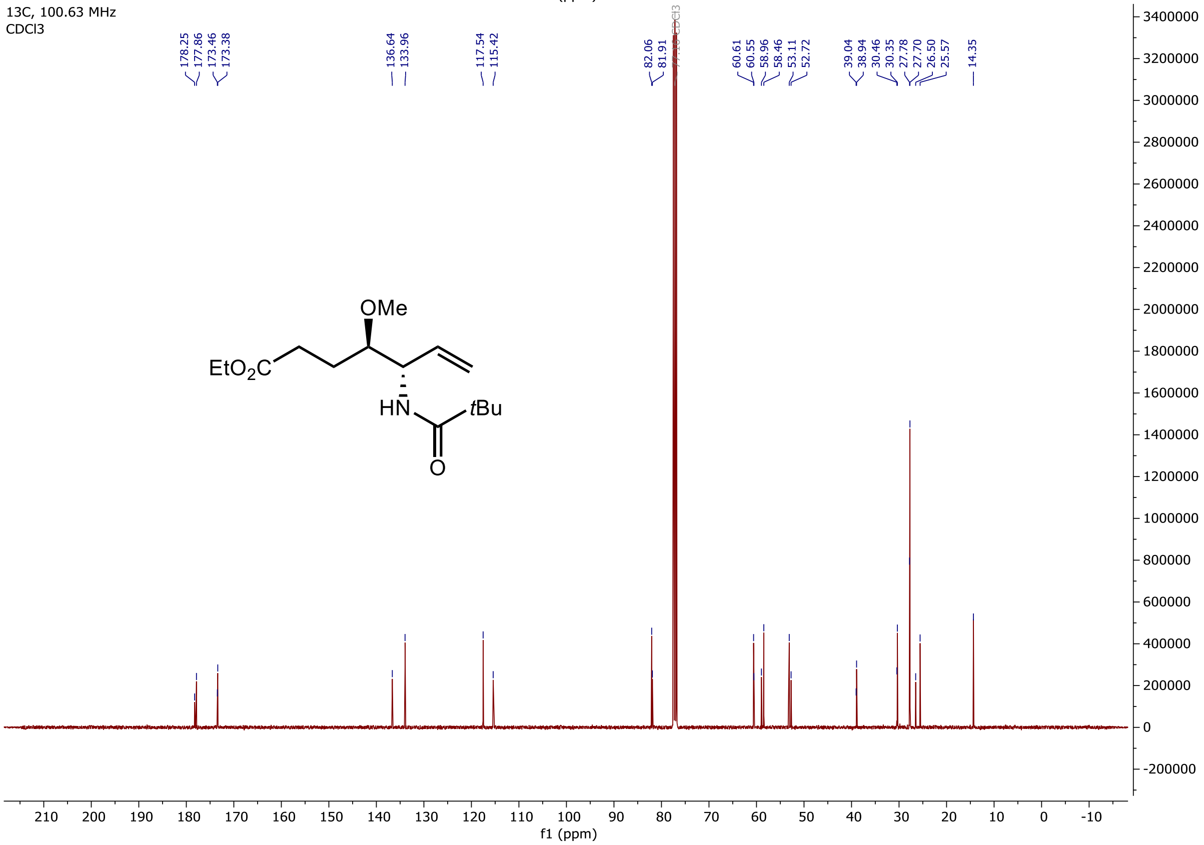

Ethyl (±)-4-methoxy-5-pivalamidohept-6-enoate (±-**22**):

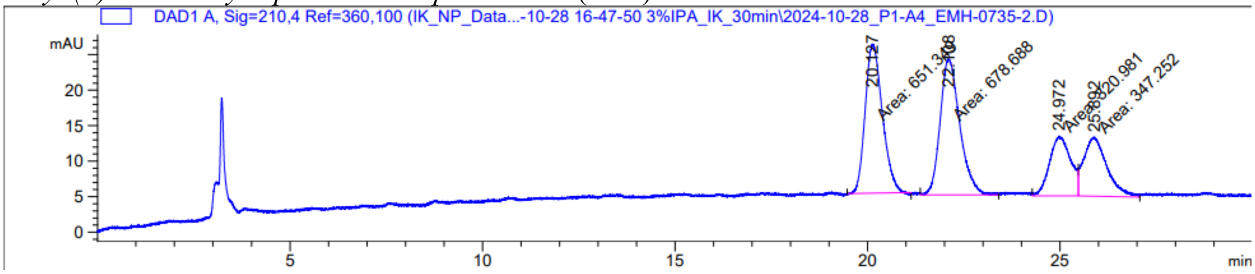

Peak #	RetTime [min]	Type	Width [min]	Area [mAU*s]	Height [mAU]	Area %
1	20.127	MM	0.5169	651.34924	21.00191	32.5957
2	22.108	MM	0.5907	678.68762	19.14783	33.9638
3	24.972	MM	0.6333	320.98087	8.44700	16.0629
4	25.892	MM	0.6957	347.25153	8.31919	17.3776

Ethyl (4R,5S)-4-methoxy-5-pivalamidohept-6-enoate (**22**):

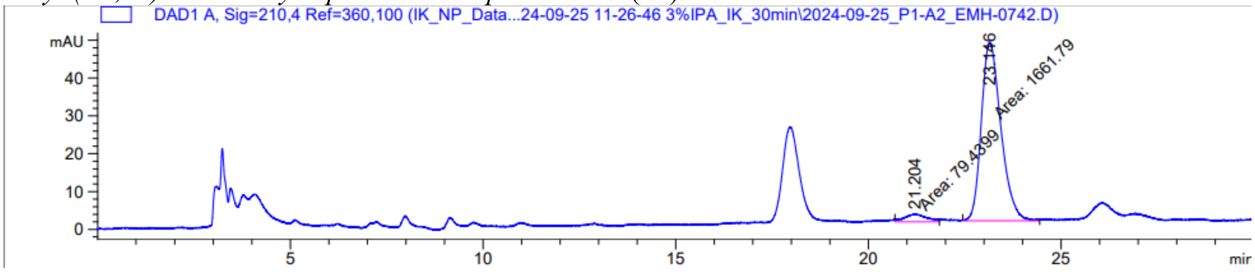

Peak #	RetTime [min]	Type	Width [min]	Area [mAU*s]	Height [mAU]	Area %
1	21.204	MM	0.6053	79.43988	2.18740	4.5623
2	23.146	MM	0.5876	1661.78784	47.13795	95.4377

Ethyl (4R,5S)-5-(hex-5-enamido)-4-methoxyhept-6-enoate (**23**):

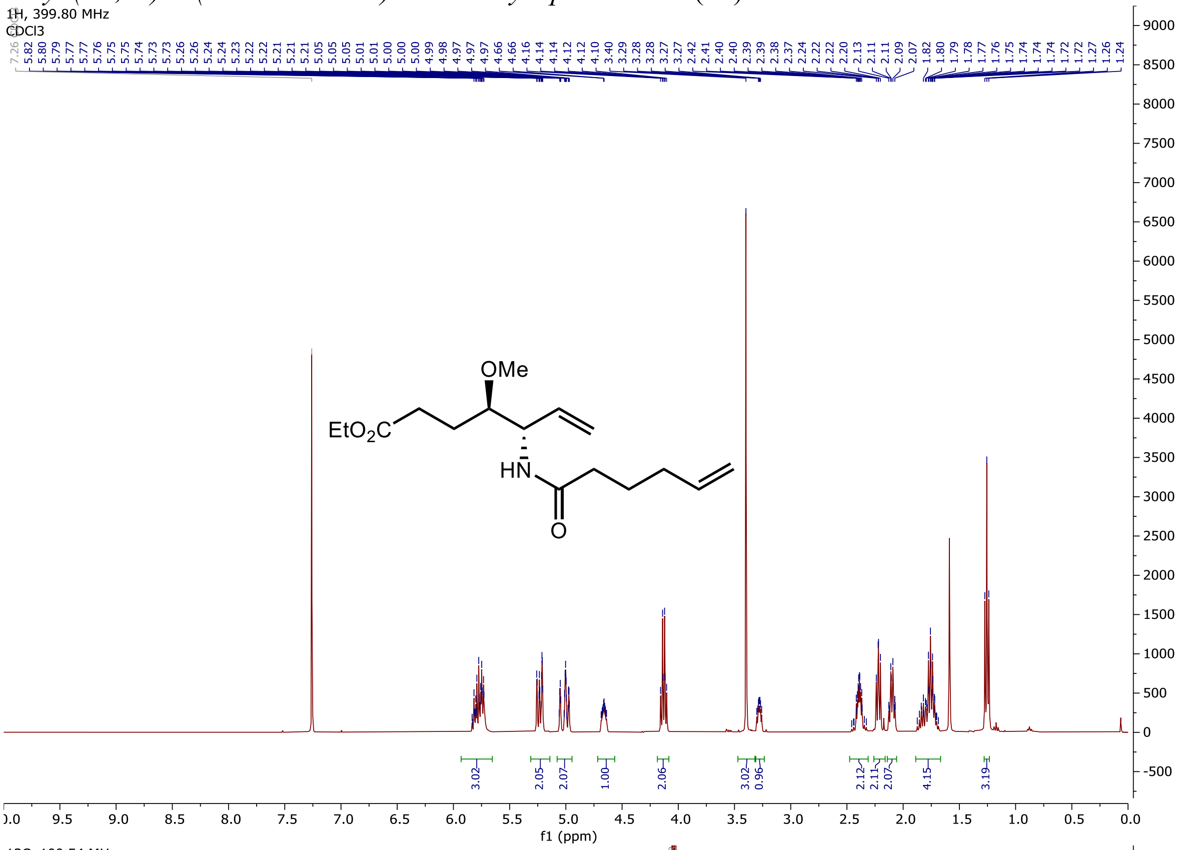

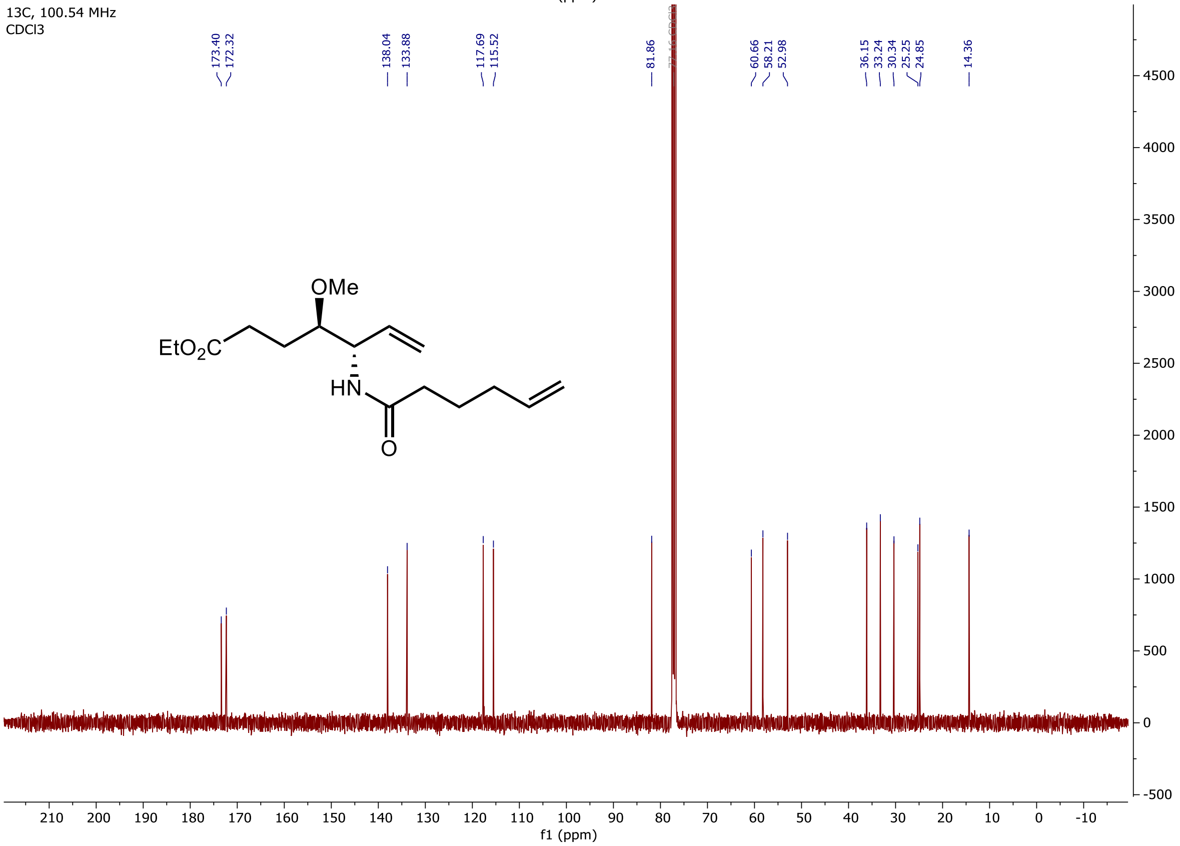

Ethyl (±)-5-(hex-5-enamido)-4-methoxyhept-6-enoate (±-**23**):

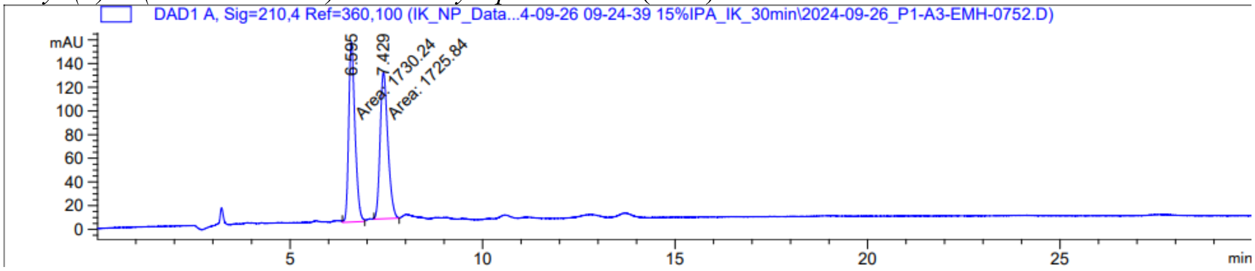

Peak #	RetTime [min]	Type	Width [min]	Area [mAU*s]	Height [mAU]	Area %
1	6.595	MM	0.1899	1730.24268	151.89420	50.0637
2	7.429	MM	0.2325	1725.83716	123.70194	49.9363

Ethyl (4R,5S)-5-(hex-5-enamido)-4-methoxyhept-6-enoate (**23**):

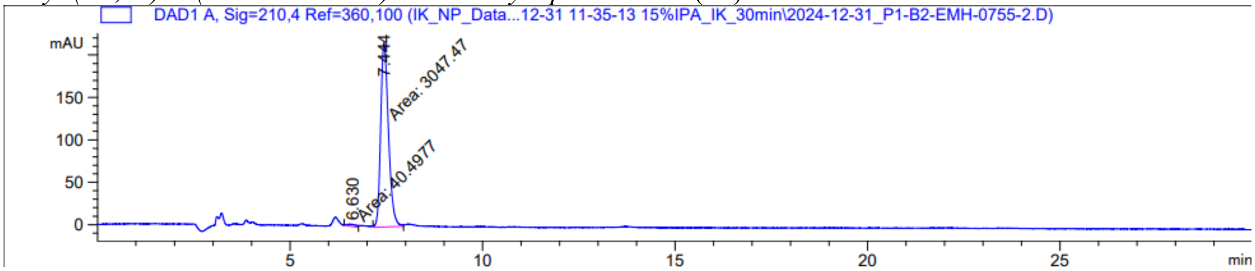

Peak #	RetTime [min]	Type	Width [min]	Area [mAU*s]	Height [mAU]	Area %
1	6.630	MM	0.2705	40.49768	2.49489	1.3115
2	7.444	MM	0.2340	3047.47021	217.01195	98.6885

Ethyl (4R,5S)-4-methoxy-5-((S)-2-phenylpropanamido)hept-6-enoate (**24a**):

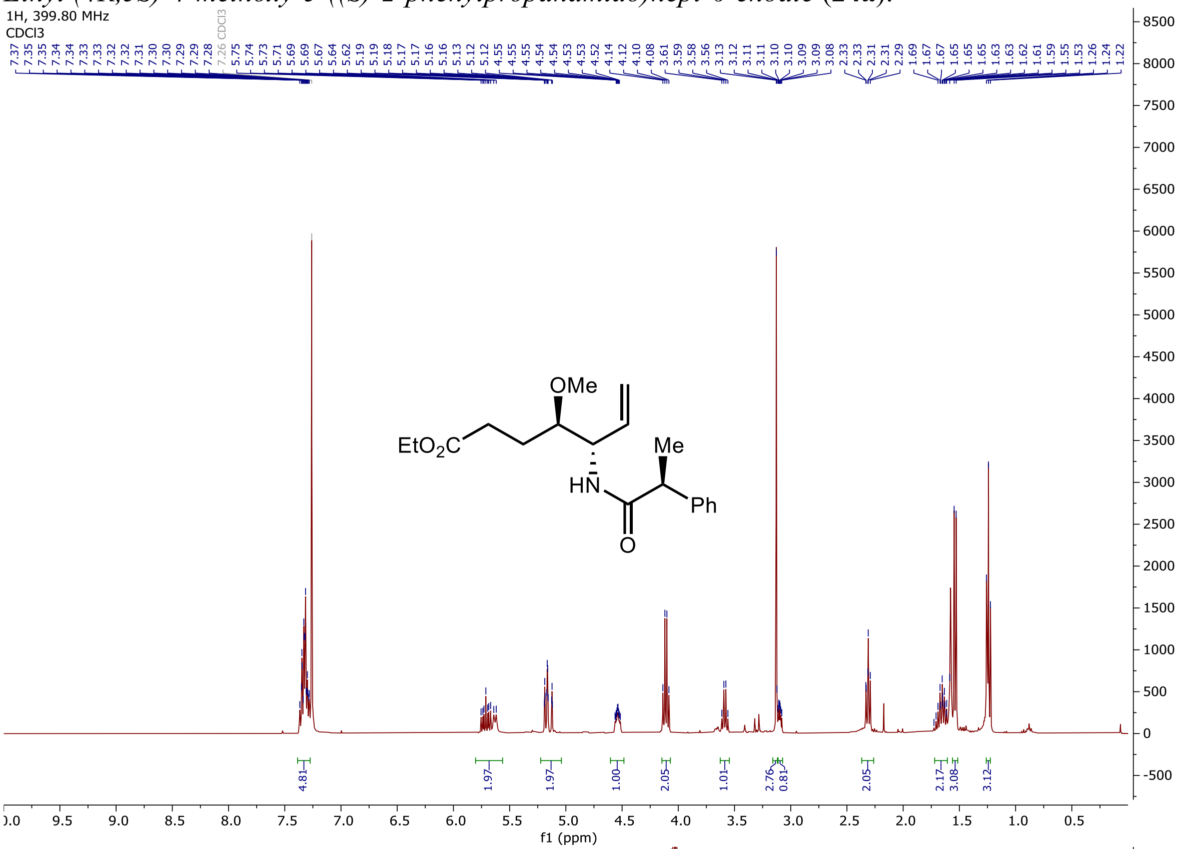

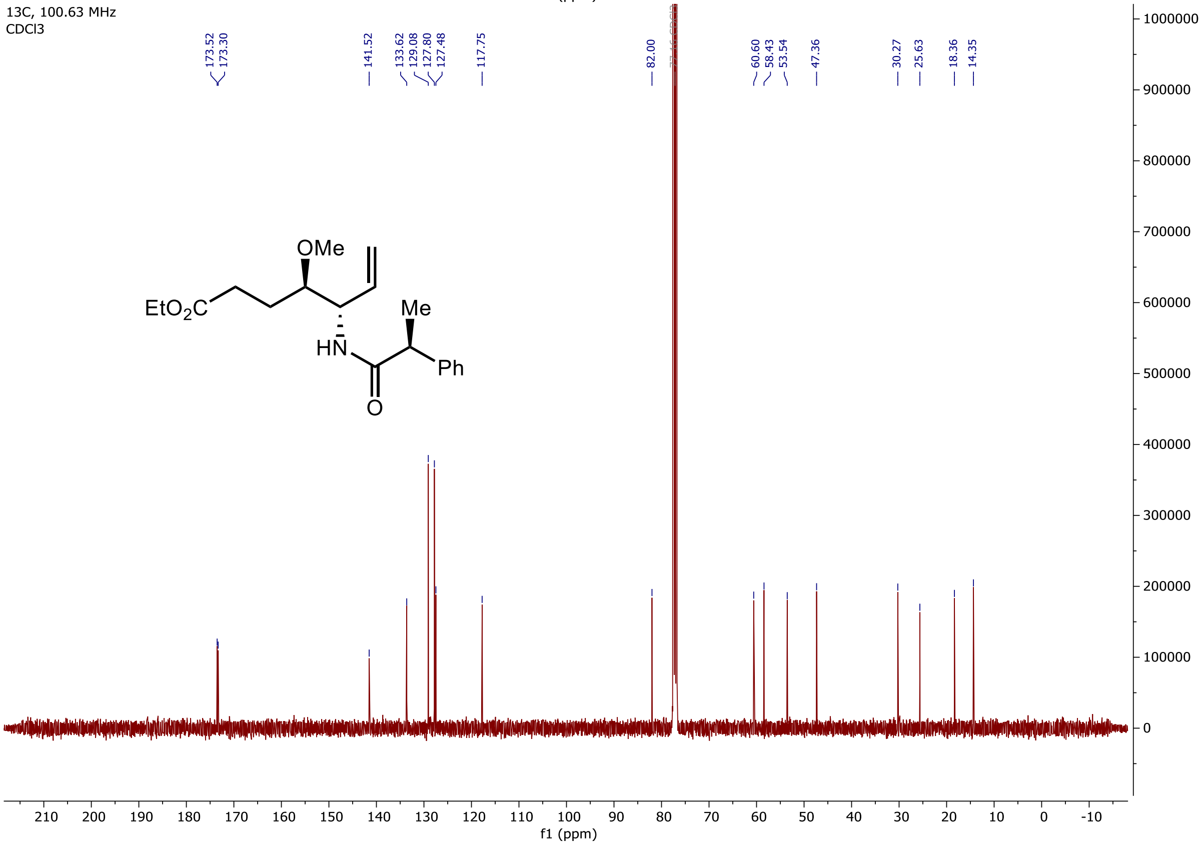

Ethyl (4S,5R)-4-methoxy-5-((S)-2-phenylpropanamido)hept-6-enoate (**24b**):

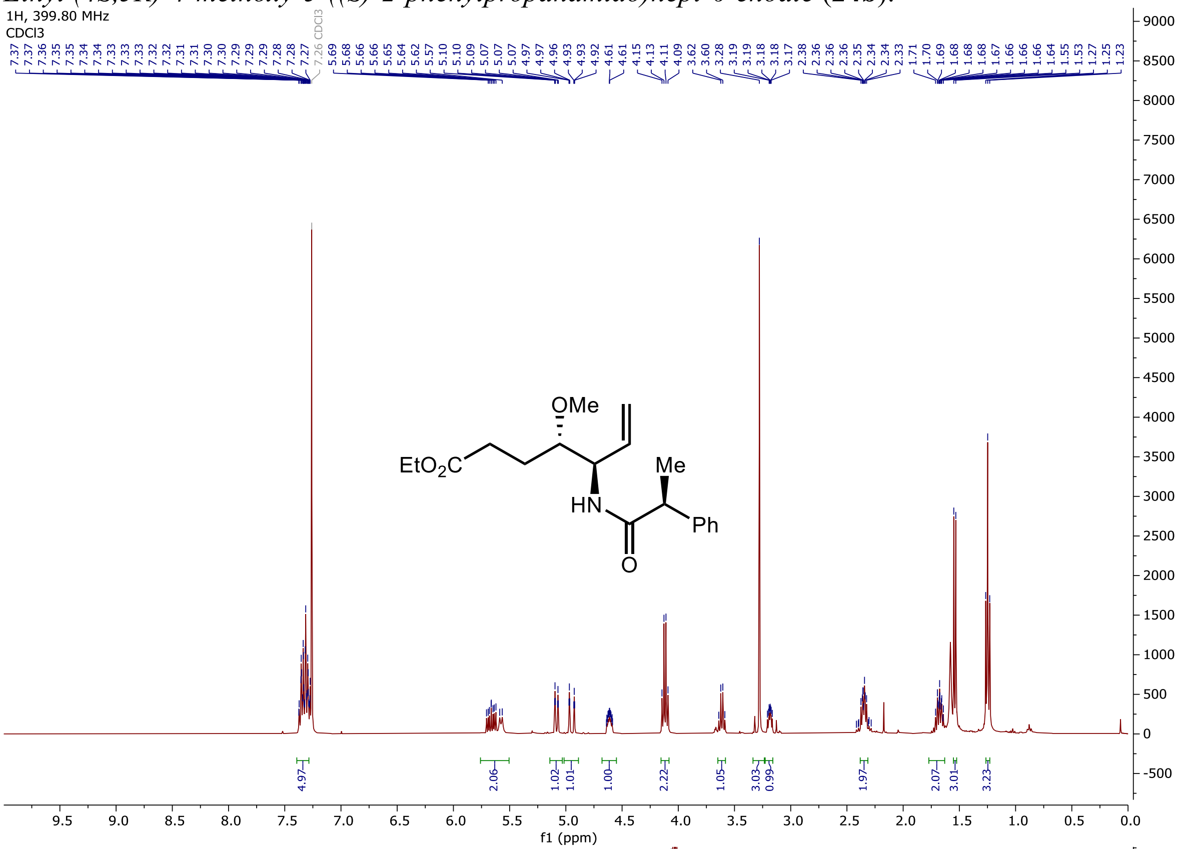

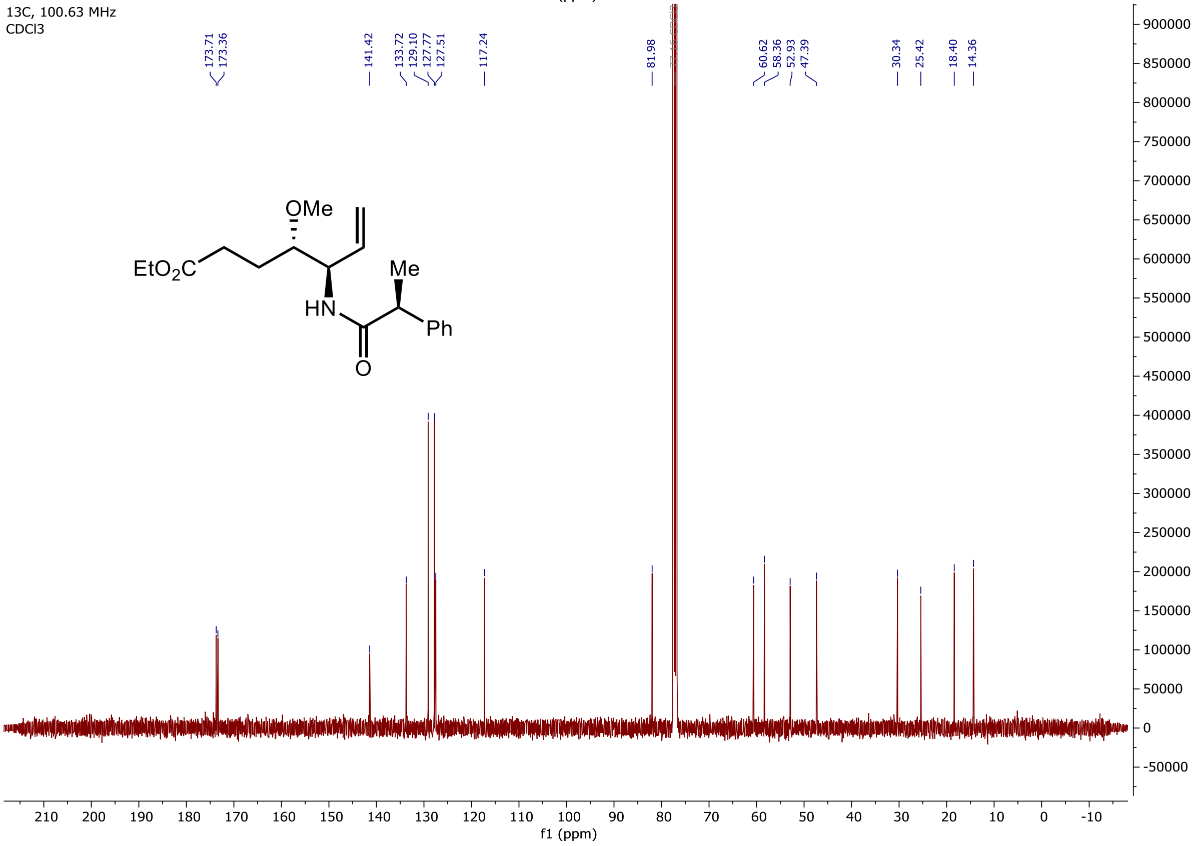

Ethyl (±)-4-methoxy-5-((S)-2-phenylpropanamido)hept-6-enoate (**±-24**):

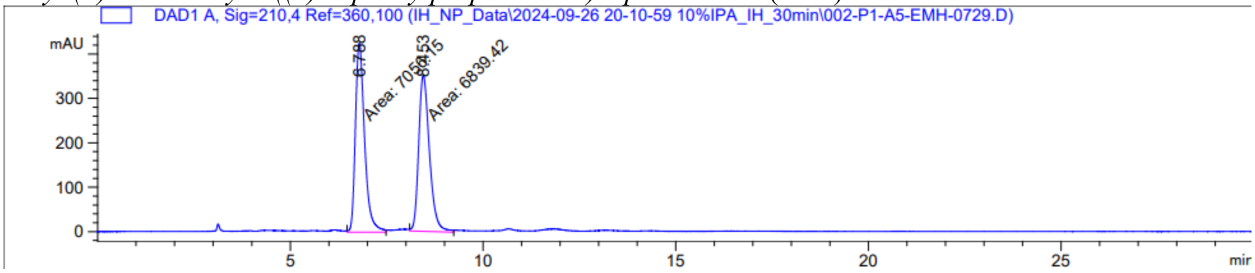

Peak #	RetTime [min]	Type	Width [min]	Area [mAU*s]	Height [mAU]	Area %
1	6.788	MM	0.2765	7056.14697	425.26071	50.7798
2	8.453	MM	0.3238	6839.41943	352.06586	49.2202

Ethyl (4R,5S)-4-methoxy-5-((S)-2-phenylpropanamido)hept-6-enoate (**24a**):

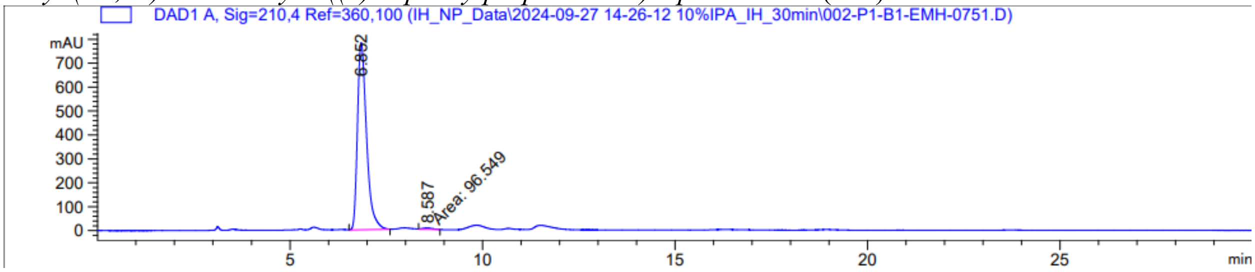

Peak #	RetTime [min]	Type	Width [min]	Area [mAU*s]	Height [mAU]	Area %
1	6.852	BV R	0.1987	1.27692e4	781.21289	99.2496
2	8.587	MM	0.2886	96.54895	5.57576	0.7504

Ethyl (4S,5R)-4-methoxy-5-((S)-2-phenylpropanamido)hept-6-enoate (**24b**):

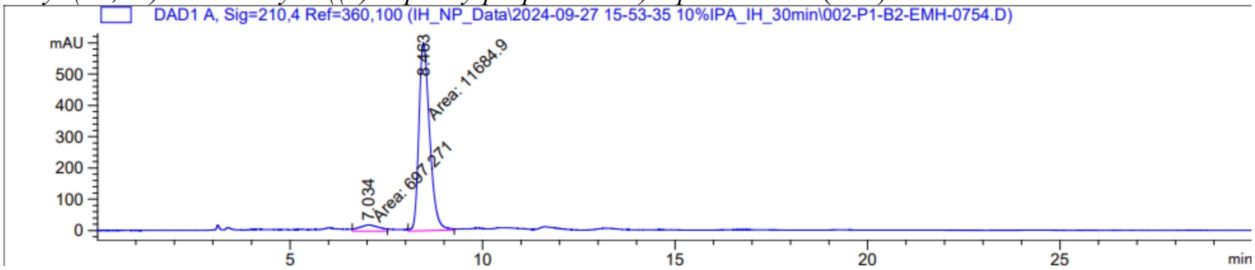

Peak #	RetTime [min]	Type	Width [min]	Area [mAU*s]	Height [mAU]	Area %
1	7.034	MM	0.5981	697.27148	19.42998	5.6313
2	8.463	MM	0.3241	1.16849e4	600.84796	94.3687

9. X-Ray Crystallographic Data

(*S,S*)-**Rh-2:**

Experimental. Single black plate-shaped crystals of (*S,S*)-**Rh-2** (CCDC 2434467) were crystallised from chloroform by slow evaporation. A suitable crystal with dimensions 0.22 × 0.10 × 0.02 mm^3 was selected and mounted on a loop with paratone on a XtaLAB Synergy-S diffractometer. The crystal was kept at a steady T = 104(6) K during data collection. The structure was solved with the ShelXT (Sheldrick, 2015) and Olex2 1.5-alpha (Dolomanov et al., 2009) as the graphical interface. The model was refined with olex2.refine 1.5-alpha (Bourhis et al., 2015) using full matrix least squares minimisation on $\boldsymbol{F^2}$.

Crystal Data. $C_{40.5}H_{42.5}Cl_{1.5}I_6Rh_2$, M_r = 1549.705, orthorhombic, $P2_12_12$ (No. 18), a = 21.1167(3) Å, b = 20.0156(3) Å, c = 10.49322(18) Å, $a = b = g = 90^{\circ}$, V = 4435.09(12) Å^3, T = 104(6) K, Z = 4, Z' = 1, m(Mo K_a) = 5.039, 97813 reflections measured, 22720 unique (R_{int} = 0.0735) which were used in all calculations. The final wR_2 was 0.0681 (all data) and R_1 was 0.0431 (I≥2 s(I)).

**Compound**	CCDC 2434467
Formula	$C_{40.5}H_{42.5}Cl_{1.5}I_6Rh_2$
$D_{calc.}$/ g cm^{-3}	2.321
m/mm^{-1}	5.039
Formula Weight	1549.705
Colour	black
Shape	plate-shaped
Size/mm^3	0.22×0.10×0.02
T/K	104(6)
Crystal System	orthorhombic
Flack Parameter	-0.009(11)
Hooft Parameter	-0.009(11)
Space Group	$P2_12_12$
a/Å	21.1167(3)
b/Å	20.0156(3)
c/Å	10.49322(18)
a/°	90
b/°	90
g/°	90
V/Å^3	4435.09(12)
Z	4
Z'	1
Wavelength/Å	0.71073
Radiation type	Mo K_a
Q_{min}/°	2.74
Q_{max}/°	37.84
Measured Refl's.	97813
Indep't Refl's	22720
Refl's I≥2 s(I)	16554
R_{int}	0.0735
Parameters	561

Restraints	716
Largest Peak	1.3470
Deepest Hole	-1.3421
GooF	0.9738
wR_2 (all data)	0.0681
wR_2	0.0622
R_1 (all data)	0.0726
R_1	0.0431

Structure Quality Indicators

**Reflections:**	d min (MoKα) 2Θ=75.7° 0.58	I/σ(I) 14.3	Rint m=4.31 7.35%	Full 50.5° 96% to 75.7° 99.7	
**Refinement:**	Shift -0.002	Max Peak 1.3	Min Peak -1.3	GooF 0.974	Hooft -.009(11)

A black plate-shaped-shaped crystal with dimensions 0.22 × 0.10 × 0.02 mm^3 was mounted on a loop with paratone. Data were collected using a XtaLAB Synergy, Dualflex, HyPix diffractometer equipped with an Oxford Cryosystems low-temperature device operating at T = 104(6) K.

Data were measured using ω scans with Mo K_α radiation. The diffraction pattern was indexed and the total number of runs and images was based on the strategy calculation from the program CrysAlisPro system (CCD 43.92a 64-bit (release 05-10-2023)). The maximum resolution that was achieved was Θ = 37.84° (0.58 Å).

The unit cell was refined using CrysAlisPro 1.171.44.57a (Rigaku OD, 2024) on 26525 reflections, 27% of the observed reflections. Data reduction, scaling and absorption corrections were performed using CrysAlisPro 1.171.44.57a (Rigaku OD, 2024). The final completeness is 99.69 % out to 37.84° in Θ. A numerical absorption correction based on gaussian integration over a multifaceted crystal model was performed using CrysAlisPro 1.171.42.74a (Rigaku Oxford Diffraction, 2022). An empirical absorption correction using spherical harmonics, implemented in SCALE3 ABSPACK scaling algorithm was also applied. The absorption coefficient μ of this material is 5.039 mm^{-1} at this wavelength (λ = 0.71073Å) and the minimum and maximum transmissions are 0.377 and 1.000.

The structure was solved and the space group $P2_12_12$ (# 18) determined by the ShelXT (Sheldrick, 2015) structure solution program using using dual methods and refined by full matrix least squares minimisation on $\boldsymbol{F}^2$ using version of olex2.refine 1.5-alpha (Bourhis et al., 2015). All non-hydrogen atoms were refined anisotropically. Hydrogen atom positions were located from the electron densities and refined using Hirshfeld scattering factors. Some hydrogen atom positions were refined using the riding model, but some hydrogen atoms were refined freely. SOFTWARE: ORCA 5.0 PARTITIONING: NoSpherA2 INT ACCURACY: Normal METHOD: PBE BASIS SET: x2c-SVP CHARGE: 0 MULTIPLICITY: 1 RELATIVISTIC: ZORA DATE: 2025-03-26_11-25-27

There is a single formula unit in the asymmetric unit (and half a chloroform molecule treated with solvent masking), which is represented by the reported sum formula. In other words: Z is 4 and Z' is 1. The moiety formula is C_{40} H_{42} I_6 Rh_2, 0.5[$CHCl_3$].

The Flack parameter is very close to zero: the refined value of the Flack parameter is -0.01(1). The absolute structure was determined using Bayesian statistics on Bijvoet differences and Olex2, resulting in a value of -0.009(11). The chiral atoms in this structure are: C1(S), C1B(S), C2(S), C2B(S), C3(R), C3B(R), C4(R), C4B(R), C5(R), C5B(R). Note: The Flack parameter is used to determine chirality of the crystal studied, the value should be near 0, a value of 1 means that the stereochemistry is wrong and the model should be inverted. A value of 0.5 means that the crystal consists of a racemic mixture of the two enantiomers.

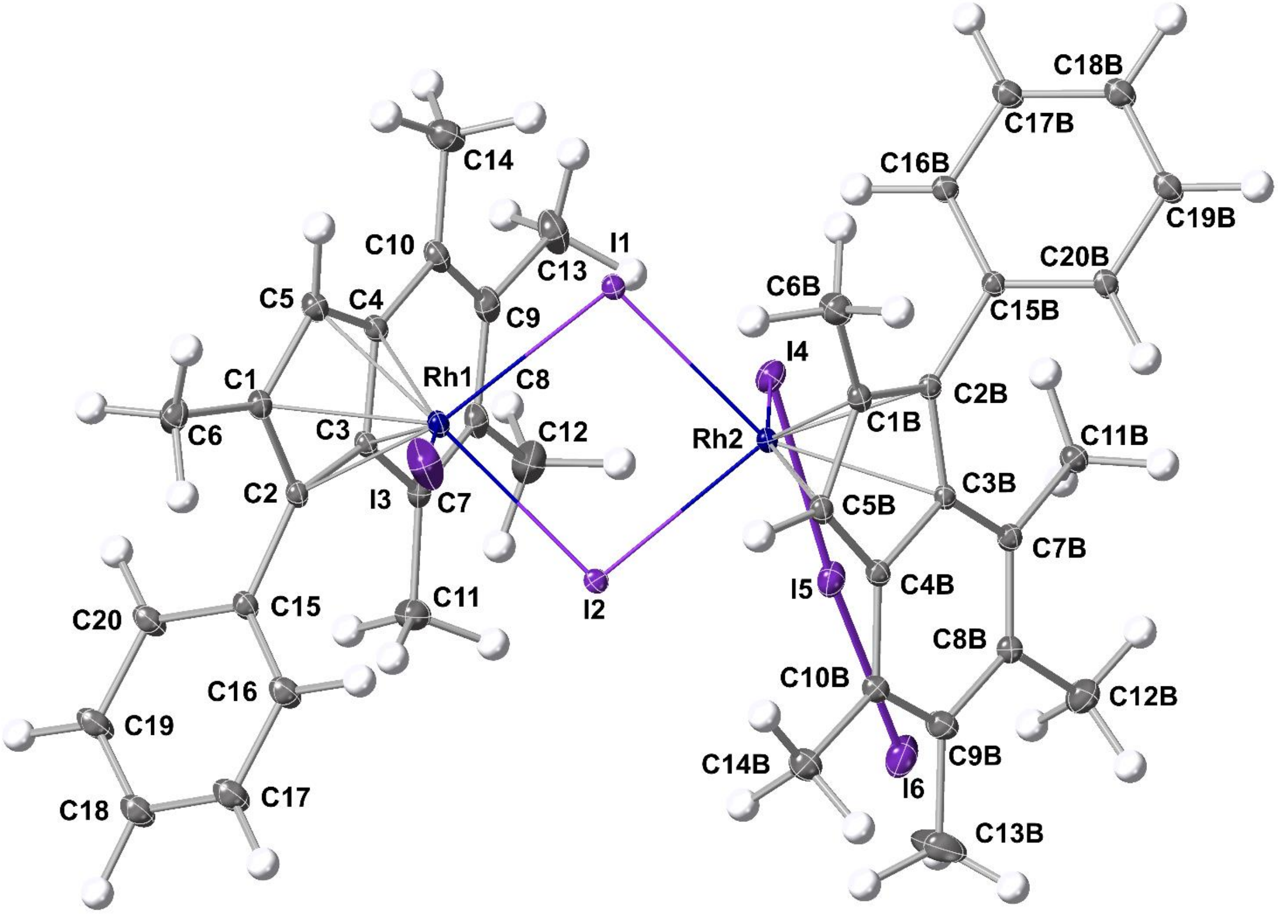

Figure 1 Thermal ellipsoidal representation of the asymmetric unit (the asymmetric unit also contains half a chloroform molecule treated with solvent masking). The chiral atoms in this structure are: C1(S), C1B(S), C2(S), C2B(S), C3(R), C3B(R), C4(R), C4B(R), C5(R), C5B(R). The ellipses are shown at the 30% probability level, and the hydrogen atoms are shown as spheres.

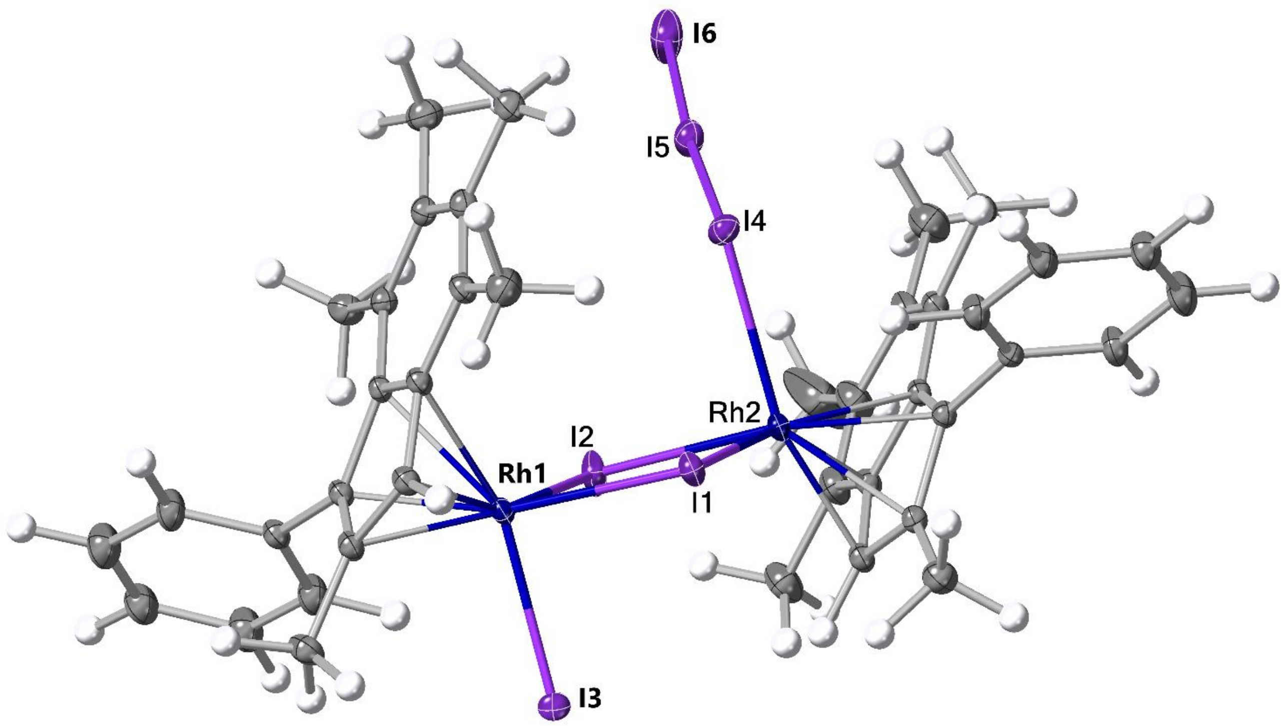

Figure 2

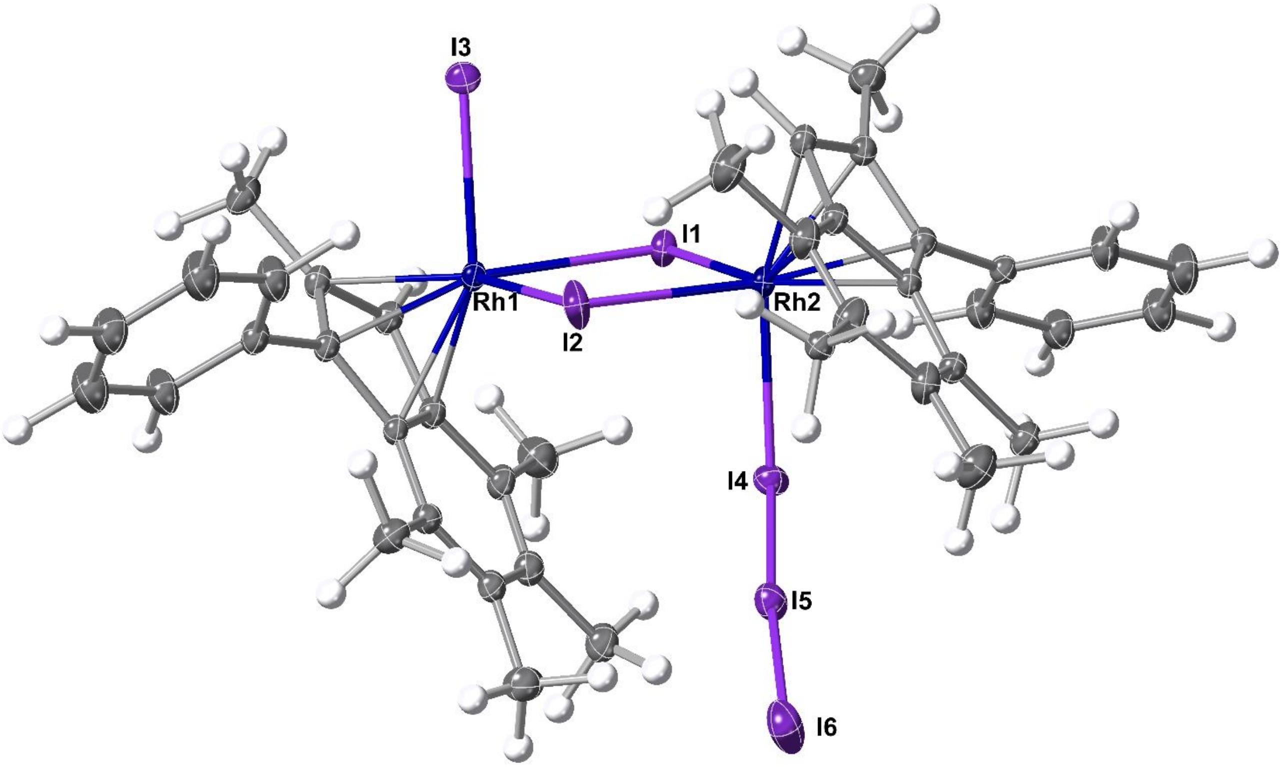

Figure 3

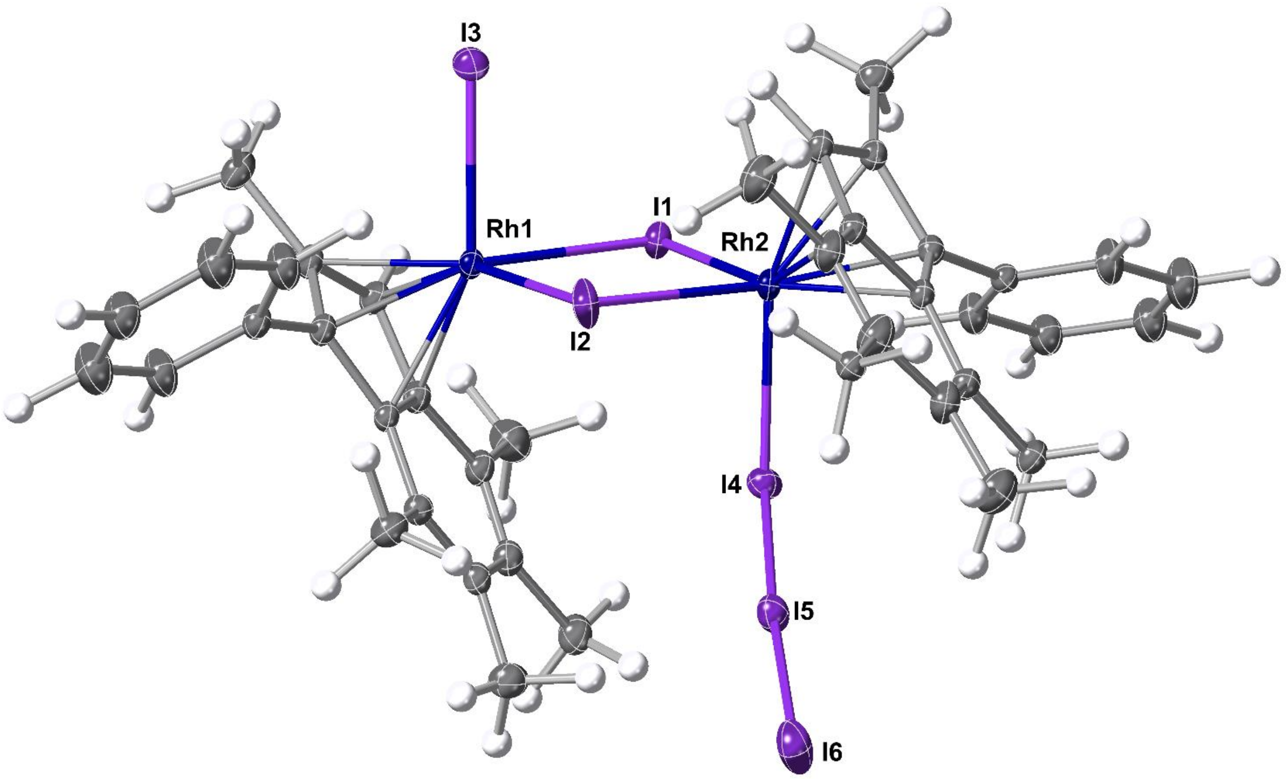

Figure 4

Data Plots: Diffraction Data

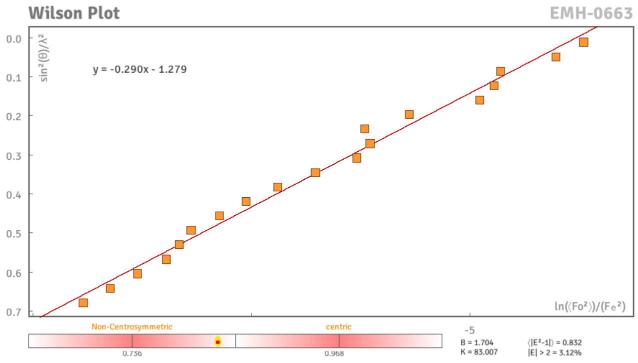

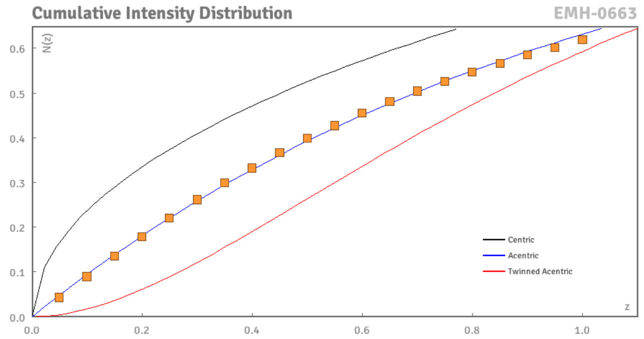

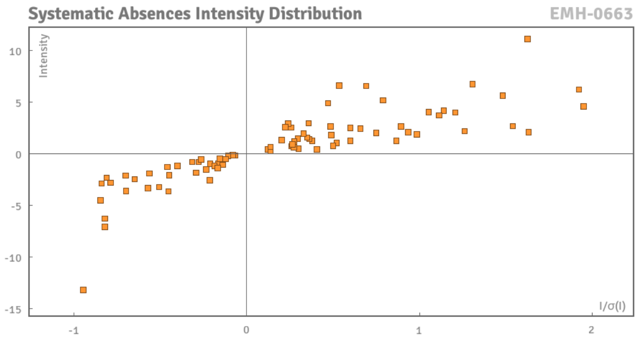

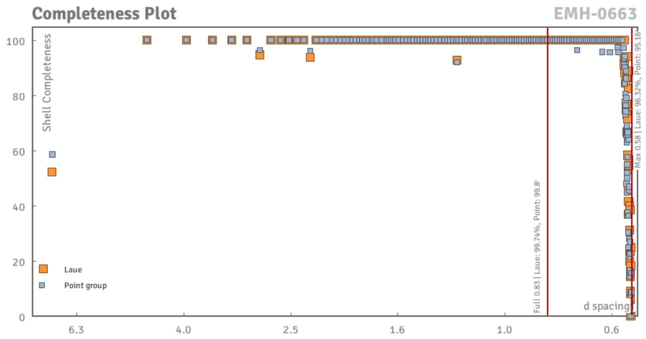

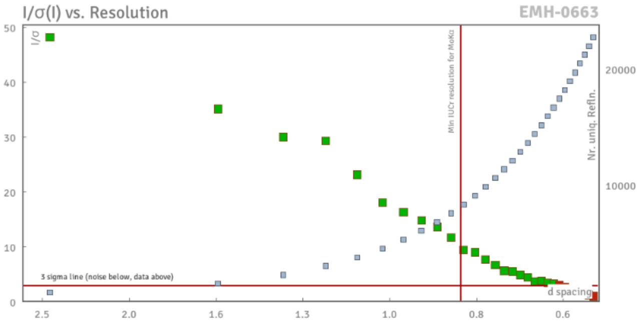

Data Plots: Refinement and Data

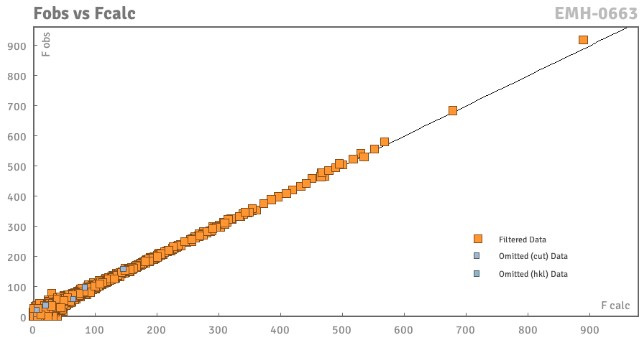

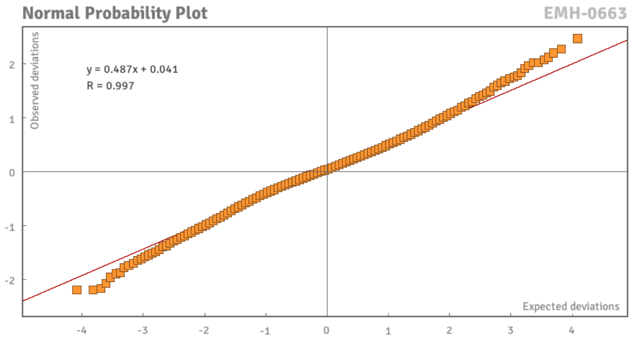

Reflection Statistics

Total reflections (after filtering)	97879	Unique reflections	22722
Completeness	0.952	Mean I/σ	11.25
hkl_{max} collected	(35, 34, 16)	hkl_{min} collected	(-35, -33, -18)
hkl_{max} used	(35, 34, 18)	hkl_{min} used	(-35, 0, 0)
Lim d_{max} collected	100.0	Lim d_{min} collected	0.36
d_{max} used	7.44	d_{min} used	0.58
Friedel pairs	19857	Friedel pairs merged	0
Inconsistent equivalents	0	R_{int}	0.0735
R_{sigma}	0.0698	Intensity transformed	0
Omitted reflections	0	Omitted by user (OMIT hkl)	25
Multiplicity	(34091, 18234, 7635, 1056, 30, 11)	Maximum multiplicity	11
Removed systematic absences	91	Filtered off (Shel/OMIT)	0

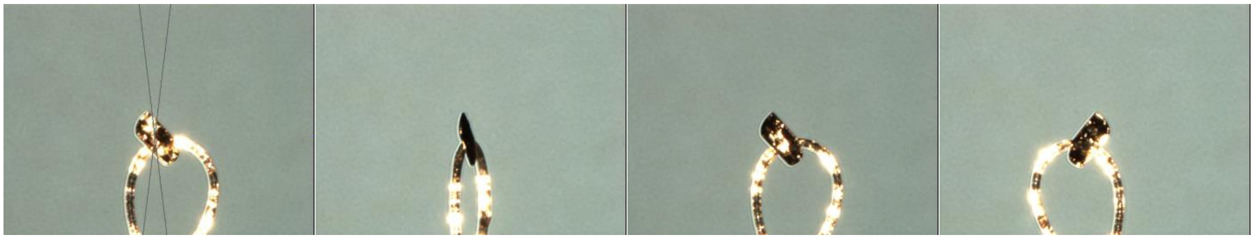

Table 1: Fractional Atomic Coordinates (×10^4) and Equivalent Isotropic Displacement Parameters (Å^2×10^3) for EMH-0663. U_{eq} is defined as 1/3 of the trace of the orthogonalised U_{ij}.

Atom	x	y	z	U_{eq}
I1	6213.3(2)	6414.2(3)	2039.3(6)	21.77(5)
I2	6188.4(3)	8235.9(3)	2111.4(7)	28.96(7)
I3	5046.3(3)	7277.9(4)	-68.1(6)	43.36(9)
I4	7279.8(3)	7221.3(3)	4370.4(5)	25.21(6)
I5	7730.5(3)	8570.0(3)	5285.6(6)	29.06(6)
I6	8237.0(3)	9769.9(3)	6280.0(8)	43.87(10)
Rh1	5261.9(3)	7316.8(3)	2400.0(6)	18.26(6)
Rh2	7121.3(3)	7320.5(3)	1834.5(6)	17.12(5)
C1	4256.5(17)	7264(2)	2841(4)	21.6(6)
C1B	7540.7(18)	6855(2)	164(4)	20.6(7)
C2	4523.6(17)	7869(2)	3349(4)	19.8(6)
C2B	8003.7(17)	6900(2)	1171(4)	18.1(6)
C3	4967.4(16)	7672(2)	4352(4)	18.2(6)
C3B	8130.4(16)	7602(2)	1394(4)	17.5(6)
C4	4997.4(18)	6963(2)	4382(4)	20.4(7)
C4B	7713.2(18)	7985(2)	575(4)	19.9(6)
C5	4585.1(19)	6717(2)	3392(4)	23.1(7)
C5B	7331.8(18)	7517(2)	-126(4)	21.4(7)
C6	3712.1(19)	7236(3)	1926(4)	30.3(9)
C6B	7348(2)	6237(2)	-485(5)	30.3(9)
C7	5336.8(18)	8061(2)	5226(4)	21.8(7)
C7B	8560.4(18)	7944(2)	2213(4)	20.2(7)
C8	5702.6(18)	7716(3)	6099(4)	25.0(7)
C8B	8584.0(19)	8626(2)	2154(5)	26.8(8)
C9	5735(2)	7002(2)	6118(5)	27.2(7)
C9B	8163(2)	9006(2)	1345(5)	33.4(10)
C10	5390.2(19)	6614(2)	5268(4)	25.1(7)
C10B	7728(2)	8698(2)	557(5)	27.1(8)
C11	5327(2)	8814(2)	5175(5)	28.5(9)
C11B	8968.5(18)	7557(2)	3137(4)	23.6(8)
C12	6074(2)	8088(3)	7093(5)	36.2(11)
C12B	9065(2)	8993(3)	2933(6)	37.7(11)
C13	6158(2)	6655(3)	7059(5)	37.5(11)
C13B	8186(3)	9754(3)	1394(9)	84(3)
C14	5406(3)	5866(2)	5223(5)	35.8(10)
C14B	7288(2)	9071(2)	-332(5)	36.9(11)
C15	4238.4(18)	8532(2)	3096(4)	22.3(7)
C15B	8373.0(17)	6314(2)	1612(4)	17.9(7)
C16	4428(2)	8953(2)	2124(5)	33.8(10)
C16B	8123(2)	5821(2)	2386(4)	25.8(8)
C17	4132(2)	9572(3)	1957(6)	41.0(12)
C17B	8482(2)	5268(2)	2732(4)	28.2(9)
C18	3642(2)	9773(3)	2768(5)	37.4(11)
C18B	9098(2)	5205(2)	2291(5)	31.2(9)
C19	3440(3)	9347(3)	3720(6)	41.6(12)
C19B	9350(2)	5688(3)	1503(5)	36.5(11)
C20	3738(2)	8729(2)	3897(5)	34.2(10)
C20B	8990(2)	6249(2)	1151(5)	29.2(10)

Table 2: Anisotropic Displacement Parameters (×10⁴) for EMH-0663. The anisotropic displacement factor exponent takes the form: $-2\pi^2[h^2a^{*2} \times U_{11} + ... + 2hka^* \times b^* \times U_{12}]$

Atom	U_{11}	U_{22}	U_{33}	U_{23}	U_{13}	U_{12}
I1	15.37(10)	17.48(11)	32.47(14)	0.54(9)	-0.05(10)	-1.11(11)
I2	19.63(12)	17.18(12)	50.1(2)	3.02(10)	11.87(12)	3.08(13)

Atom	U_{11}	U_{22}	U_{33}	U_{23}	U_{13}	U_{12}
I3	35.73(16)	74.7(3)	19.69(13)	21.69(18)	-2.36(12)	0.43(17)
I4	24.66(12)	30.28(15)	20.68(12)	-5.68(11)	3.21(10)	-1.11(11)
I5	21.83(12)	32.46(15)	32.90(15)	-3.00(11)	4.67(11)	-5.24(12)
I6	29.35(15)	33.58(18)	68.7(3)	-7.58(13)	15.35(16)	-18.42(18)
Rh1	13.65(11)	20.93(14)	20.21(13)	2.38(11)	-0.73(10)	1.24(13)
Rh2	12.61(11)	17.34(13)	21.40(13)	1.38(10)	0.48(10)	0.65(13)
C1	15.5(11)	24.6(10)	24.6(14)	1.5(6)	-0.3(7)	2.1(6)
C1B	17.6(12)	24.3(10)	19.9(13)	3.5(6)	0.2(7)	1.0(6)
C2	14.8(12)	23.5(9)	20.9(13)	2.1(5)	-0.6(7)	3.9(5)
C2B	16.0(11)	19.8(8)	18.4(13)	1.7(4)	1.7(7)	3.2(5)
C3	14.4(11)	21.1(9)	19.2(11)	3.4(6)	0.3(6)	2.0(6)
C3B	14.4(11)	19.7(9)	18.4(12)	2.0(5)	1.1(7)	3.5(5)
C4	17.6(13)	21.1(9)	22.4(13)	3.5(6)	-0.9(7)	2.2(6)
C4B	16.7(12)	21.8(8)	21.2(14)	2.4(5)	-0.8(7)	4.9(5)
C5	19.8(14)	24.9(10)	24.7(14)	3.0(6)	-2.5(8)	0.7(6)
C5B	15.9(13)	24.2(10)	24.1(16)	2.6(6)	-0.2(7)	1.9(6)
C6	20.3(13)	40(3)	31.0(17)	-4.5(12)	-5.9(8)	8.3(15)
C6B	31(2)	27.7(13)	32(2)	1.7(9)	-4.7(15)	-4.1(9)
C7	17.9(13)	27.4(11)	20.1(12)	-0.6(6)	0.6(7)	0.2(6)
C7B	16.9(12)	22.4(10)	21.1(13)	0.8(5)	-0.7(7)	2.6(6)
C8	18.4(14)	36.7(11)	20.0(13)	4.1(7)	3.5(7)	3.4(7)
C8B	18.8(14)	22.4(10)	39.3(19)	0.9(5)	0.4(9)	3.4(6)
C9	20.6(15)	36.7(11)	24.4(15)	4.3(7)	2.1(8)	3.5(7)
C9B	28.0(15)	23.5(11)	49(2)	2.0(6)	-8.3(10)	3.6(7)
C10	20.3(14)	30.5(12)	24.5(14)	7.6(6)	-0.4(8)	5.9(6)
C10B	21.1(14)	21.6(9)	38.7(18)	2.1(5)	-0.5(9)	5.2(5)
C11	29(2)	27.4(11)	29(2)	-0.7(6)	-6.0(17)	0.3(6)
C11B	19.7(15)	25.8(19)	25.4(17)	0.8(10)	-3.2(9)	5.2(10)
C12	32(2)	51(3)	25.8(19)	-4.2(13)	-0.6(10)	-0.6(12)
C12B	31(2)	34(2)	48(3)	-7.8(11)	-6.6(12)	3.6(13)
C13	28(2)	56(3)	29(2)	12.3(13)	1.2(10)	9.5(13)
C13B	71(4)	23.5(11)	157(7)	2.5(7)	-64(4)	1.7(7)
C14	43(3)	30.4(12)	34(2)	8.0(7)	5(2)	5.8(6)
C14B	29(2)	32(2)	50(3)	4.0(12)	-6.6(12)	11.8(12)
C15	17.7(13)	24.1(9)	25.1(14)	3.2(6)	-1.2(9)	4.3(7)
C15B	17.4(10)	18.8(10)	17.4(15)	1.7(5)	-0.6(7)	1.2(7)
C16	30.1(18)	33.1(15)	38.2(18)	10.9(9)	9.9(11)	14.8(9)
C16B	22.1(14)	23.8(13)	31.6(19)	4.5(7)	5.9(9)	9.3(8)
C17	39(2)	35.3(16)	49(2)	15.2(10)	7.7(13)	15.1(9)
C17B	28.0(13)	25.1(15)	32(2)	7.4(8)	2.7(10)	7.1(9)
C18	34.6(19)	34.4(19)	43(2)	13.4(10)	2.8(12)	13.2(11)
C18B	28.8(13)	28.0(17)	37(2)	8.6(8)	4.8(10)	9.3(11)
C19	42(2)	34.2(16)	48(2)	18.7(9)	10.5(13)	15.2(11)
C19B	28.3(15)	32.9(15)	48(3)	12.1(7)	8.7(11)	17.1(12)
C20	33.0(17)	32.1(16)	37.6(19)	14.4(9)	12.7(11)	14.7(10)
C20B	21.3(11)	28.3(17)	38(2)	7.2(7)	8.3(8)	13.4(11)

Table 3: Bond Lengths in Å for EMH-0663.

Atom	Atom	Length/Å
I1	Rh1	2.7278(8)
I1	Rh2	2.6478(8)
I2	Rh1	2.7021(8)
I2	Rh2	2.7056(8)
I3	Rh1	2.6297(8)

Atom	Atom	Length/Å
I4	I5	3.0183(8)
I4	Rh2	2.6882(8)
I5	I6	2.8277(9)
Rh1	C1	2.175(4)
Rh1	C2	2.154(4)

Atom	Atom	Length/Å
Rh1	C3	2.255(4)
Rh1	C4	2.266(4)
Rh1	C5	2.136(4)
Rh2	C1B	2.172(4)
Rh2	C2B	2.160(4)
Rh2	C3B	2.252(3)
Rh2	C4B	2.253(4)
Rh2	C5B	2.140(4)
C1	C2	1.437(6)
C1	C5	1.418(6)
C1	C6	1.499(5)
C1B	C2B	1.442(6)
C1B	C5B	1.427(6)
C1B	C6B	1.470(6)
C2	C3	1.463(5)
C2	C15	1.482(6)
C2B	C3B	1.450(5)
C2B	C15B	1.482(5)
C3	C4	1.420(6)
C3	C7	1.434(6)
C3B	C4B	1.450(5)
C3B	C7B	1.425(6)
C4	C5	1.442(6)
C4	C10	1.428(6)
C4B	C5B	1.438(6)
C4B	C10B	1.426(6)
C5	H5	1.21(6)
C5B	H5B	1.12(6)
C6	H6a	1.0970
C6	H6b	1.0970
C6	H6c	1.0970
C6B	H6Ba	1.0970
C6B	H6Bb	1.0970
C6B	H6Bc	1.0970
C7	C8	1.383(6)
C7	C11	1.509(6)
C7B	C8B	1.366(6)
C7B	C11B	1.510(5)
C8	C9	1.432(7)
C8	C12	1.502(7)
C8B	C9B	1.445(6)
C8B	C12B	1.497(6)
C9	C10	1.389(6)
C9	C13	1.501(6)
C9B	C10B	1.382(7)
C9B	C13B	1.499(7)
C10	C14	1.498(6)

Atom	Atom	Length/Å
C10B	C14B	1.514(6)
C11	H11a	1.0970
C11	H11b	1.0970
C11	H11c	1.0970
C11B	H11d	1.0970
C11B	H11e	1.0970
C11B	H11f	1.0970
C12	H12a	1.0970
C12	H12b	1.0970
C12	H12c	1.0970
C12B	H12d	1.0970
C12B	H12e	1.0970
C12B	H12f	1.0970
C13	H13a	1.0970
C13	H13b	1.0970
C13	H13c	1.0970
C13B	H13d	1.0970
C13B	H13e	1.0970
C13B	H13f	1.0970
C14	H14a	1.0970
C14	H14b	1.0970
C14	H14c	1.0970
C14B	H14d	1.0970
C14B	H14e	1.0970
C14B	H14f	1.0970
C15	C16	1.381(6)
C15	C20	1.406(6)
C15B	C16B	1.383(6)
C15B	C20B	1.396(5)
C16	H16	1.1030
C16	C17	1.399(6)
C16B	H16B	1.1030
C16B	C17B	1.389(6)
C17	H17	1.1030
C17	C18	1.397(7)
C17B	H17B	1.1030
C17B	C18B	1.386(6)
C18	H18	1.1030
C18	C19	1.381(7)
C18B	H18B	1.1030
C18B	C19B	1.378(7)
C19	H19	1.1030
C19	C20	1.400(6)
C19B	H19B	1.1030
C19B	C20B	1.405(6)
C20	H20	1.1030
C20B	H20B	1.1030

Table 4: Bond Angles in ° for EMH-0663.

Atom	Atom	Atom	Angle/°
Rh2	I1	Rh1	95.23(3)
Rh2	I2	Rh1	94.50(2)
Rh2	I4	I5	106.74(3)
I6	I5	I4	174.70(3)

Atom	Atom	Atom	Angle/°
I2	Rh1	I1	84.36(2)
I3	Rh1	I1	88.36(3)
I3	Rh1	I2	92.02(3)
C1	Rh1	I1	135.73(12)

Atom	Atom	Atom	Angle/˚
C1	Rh1	I2	139.78(12)
C1	Rh1	I3	92.23(11)
C2	Rh1	I1	159.46(11)
C2	Rh1	I2	103.12(11)
C2	Rh1	I3	110.17(11)
C2	Rh1	C1	38.78(15)
C3	Rh1	I1	122.52(10)
C3	Rh1	I2	95.00(11)
C3	Rh1	I3	148.83(10)
C3	Rh1	C1	63.45(14)
C3	Rh1	C2	38.67(13)
C4	Rh1	I1	95.85(10)
C4	Rh1	I2	119.60(11)
C4	Rh1	I3	148.34(11)
C4	Rh1	C1	63.20(14)
C4	Rh1	C2	63.75(15)
C4	Rh1	C3	36.61(14)
C5	Rh1	I1	100.86(12)
C5	Rh1	I2	157.10(12)
C5	Rh1	I3	110.29(12)
C5	Rh1	C1	38.39(15)
C5	Rh1	C2	65.08(15)
C5	Rh1	C3	63.27(16)
C5	Rh1	C4	38.09(15)
I2	Rh2	I1	85.85(2)
I4	Rh2	I1	87.67(3)
I4	Rh2	I2	91.97(3)
C1B	Rh2	I1	93.86(11)
C1B	Rh2	I2	132.32(11)
C1B	Rh2	I4	135.70(11)
C2B	Rh2	I1	112.57(11)
C2B	Rh2	I2	157.92(11)
C2B	Rh2	I4	100.54(11)
C2B	Rh2	C1B	38.87(15)
C3B	Rh2	I1	150.83(10)
C3B	Rh2	I2	122.79(10)
C3B	Rh2	I4	95.96(10)
C3B	Rh2	C1B	63.64(15)
C3B	Rh2	C2B	38.30(14)
C4B	Rh2	I1	148.61(11)
C4B	Rh2	I2	93.84(10)
C4B	Rh2	I4	123.68(11)
C4B	Rh2	C1B	63.52(15)
C4B	Rh2	C2B	64.08(14)
C4B	Rh2	C3B	37.54(14)
C5B	Rh2	I1	110.73(11)
C5B	Rh2	I2	97.47(11)
C5B	Rh2	I4	159.79(11)
C5B	Rh2	C1B	38.65(15)
C5B	Rh2	C2B	65.33(15)
C5B	Rh2	C3B	63.92(14)
C5B	Rh2	C4B	38.12(15)
C2	C1	Rh1	69.8(2)
C5	C1	Rh1	69.3(2)
C5	C1	C2	107.8(3)
C6	C1	Rh1	127.9(3)
C6	C1	C2	124.7(4)

Atom	Atom	Atom	Angle/˚
C6	C1	C5	127.4(4)
C2B	C1B	Rh2	70.1(2)
C5B	C1B	Rh2	69.4(2)
C5B	C1B	C2B	108.0(4)
C6B	C1B	Rh2	128.4(3)
C6B	C1B	C2B	125.3(4)
C6B	C1B	C5B	126.6(4)
C1	C2	Rh1	71.4(2)
C3	C2	Rh1	74.4(2)
C3	C2	C1	106.9(3)
C15	C2	Rh1	132.1(3)
C15	C2	C1	121.9(3)
C15	C2	C3	129.1(4)
C1B	C2B	Rh2	71.0(2)
C3B	C2B	Rh2	74.3(2)
C3B	C2B	C1B	107.6(3)
C15B	C2B	Rh2	131.4(3)
C15B	C2B	C1B	122.5(4)
C15B	C2B	C3B	128.3(3)
C2	C3	Rh1	66.9(2)
C4	C3	Rh1	72.1(3)
C4	C3	C2	108.3(4)
C7	C3	Rh1	127.0(3)
C7	C3	C2	131.4(4)
C7	C3	C4	120.3(4)
C2B	C3B	Rh2	67.4(2)
C4B	C3B	Rh2	71.3(2)
C4B	C3B	C2B	107.8(3)
C7B	C3B	Rh2	126.8(3)
C7B	C3B	C2B	132.9(3)
C7B	C3B	C4B	119.4(4)
C3	C4	Rh1	71.3(2)
C5	C4	Rh1	66.1(2)
C5	C4	C3	107.3(4)
C10	C4	Rh1	127.3(3)
C10	C4	C3	121.9(4)
C10	C4	C5	130.7(4)
C3B	C4B	Rh2	71.2(2)
C5B	C4B	Rh2	66.7(2)
C5B	C4B	C3B	107.3(3)
C10B	C4B	Rh2	127.6(3)
C10B	C4B	C3B	121.6(4)
C10B	C4B	C5B	131.1(4)
C1	C5	Rh1	72.3(2)
C4	C5	Rh1	75.8(2)
C4	C5	C1	109.0(4)
H5	C5	Rh1	117(3)
H5	C5	C1	128(3)
H5	C5	C4	123(3)
C1B	C5B	Rh2	71.9(2)
C4B	C5B	Rh2	75.2(2)
C4B	C5B	C1B	108.8(3)
H5B	C5B	Rh2	117(3)
H5B	C5B	C1B	119(3)
H5B	C5B	C4B	132(3)
H6a	C6	C1	109.5
H6b	C6	C1	109.5

Atom	Atom	Atom	Angle/°
H6b	C6	H6a	109.5
H6c	C6	C1	109.5
H6c	C6	H6a	109.5
H6c	C6	H6b	109.5
H6Ba	C6B	C1B	109.5
H6Bb	C6B	C1B	109.5
H6Bb	C6B	H6Ba	109.5
H6Bc	C6B	C1B	109.5
H6Bc	C6B	H6Ba	109.5
H6Bc	C6B	H6Bb	109.5
C8	C7	C3	117.1(4)
C11	C7	C3	120.9(4)
C11	C7	C8	122.0(4)
C8B	C7B	C3B	118.3(4)
C11B	C7B	C3B	120.3(4)
C11B	C7B	C8B	121.4(4)
C9	C8	C7	122.3(4)
C12	C8	C7	120.3(4)
C12	C8	C9	117.3(4)
C9B	C8B	C7B	122.0(4)
C12B	C8B	C7B	119.3(4)
C12B	C8B	C9B	118.7(4)
C10	C9	C8	121.6(4)
C13	C9	C8	120.0(4)
C13	C9	C10	118.4(4)
C10B	C9B	C8B	121.7(4)
C13B	C9B	C8B	119.1(5)
C13B	C9B	C10B	119.2(5)
C9	C10	C4	116.7(4)
C14	C10	C4	118.8(4)
C14	C10	C9	124.5(4)
C9B	C10B	C4B	116.9(4)
C14B	C10B	C4B	119.2(4)
C14B	C10B	C9B	123.8(4)
H11a	C11	C7	109.5
H11b	C11	C7	109.5
H11b	C11	H11a	109.5
H11c	C11	C7	109.5
H11c	C11	H11a	109.5
H11c	C11	H11b	109.5
H11d	C11B	C7B	109.5
H11e	C11B	C7B	109.5
H11e	C11B	H11d	109.5
H11f	C11B	C7B	109.5
H11f	C11B	H11d	109.5
H11f	C11B	H11e	109.5
H12a	C12	C8	109.5
H12b	C12	C8	109.5
H12b	C12	H12a	109.5
H12c	C12	C8	109.5
H12c	C12	H12a	109.5
H12c	C12	H12b	109.5
H12d	C12B	C8B	109.5
H12e	C12B	C8B	109.5
H12e	C12B	H12d	109.5
H12f	C12B	C8B	109.5
H12f	C12B	H12d	109.5

Atom	Atom	Atom	Angle/°
H12f	C12B	H12e	109.5
H13a	C13	C9	109.5
H13b	C13	C9	109.5
H13b	C13	H13a	109.5
H13c	C13	C9	109.5
H13c	C13	H13a	109.5
H13c	C13	H13b	109.5
H13d	C13B	C9B	109.5
H13e	C13B	C9B	109.5
H13e	C13B	H13d	109.5
H13f	C13B	C9B	109.5
H13f	C13B	H13d	109.5
H13f	C13B	H13e	109.5
H14a	C14	C10	109.5
H14b	C14	C10	109.5
H14b	C14	H14a	109.5
H14c	C14	C10	109.5
H14c	C14	H14a	109.5
H14c	C14	H14b	109.5
H14d	C14B	C10B	109.5
H14e	C14B	C10B	109.5
H14e	C14B	H14d	109.5
H14f	C14B	C10B	109.5
H14f	C14B	H14d	109.5
H14f	C14B	H14e	109.5
C16	C15	C2	124.1(4)
C20	C15	C2	116.7(4)
C20	C15	C16	119.2(4)
C16B	C15B	C2B	123.1(3)
C20B	C15B	C2B	117.2(4)
C20B	C15B	C16B	119.6(4)
H16	C16	C15	119.9(3)
C17	C16	C15	120.2(5)
C17	C16	H16	119.9(3)
H16B	C16B	C15B	119.6(2)
C17B	C16B	C15B	120.8(4)
C17B	C16B	H16B	119.6(3)
H17	C17	C16	119.7(3)
C18	C17	C16	120.6(5)
C18	C17	H17	119.7(3)
H17B	C17B	C16B	120.1(3)
C18B	C17B	C16B	119.9(4)
C18B	C17B	H17B	120.1(3)
H18	C18	C17	120.3(3)
C19	C18	C17	119.4(5)
C19	C18	H18	120.3(3)
H18B	C18B	C17B	120.0(3)
C19B	C18B	C17B	119.9(4)
C19B	C18B	H18B	120.0(3)
H19	C19	C18	119.9(3)
C20	C19	C18	120.2(5)
C20	C19	H19	119.9(3)
H19B	C19B	C18B	119.7(3)
C20B	C19B	C18B	120.6(4)
C20B	C19B	H19B	119.7(3)
C19	C20	C15	120.4(5)
H20	C20	C15	119.8(3)

Atom	Atom	Atom	Angle/˚
H20	C20	C19	119.8(3)
C19B	C20B	C15B	119.2(4)

Atom	Atom	Atom	Angle/˚
H20B	C20B	C15B	120.4(2)
H20B	C20B	C19B	120.4(3)

Table 5: Torsion Angles in ˚ for EMH-0663.

Atom	Atom	Atom	Atom	Angle/˚
Rh1	C1	C2	C3	-66.5(2)
Rh1	C1	C2	C15	128.6(2)
Rh1	C1	C5	C4	67.6(2)
Rh1	C2	C1	C5	59.1(3)
Rh1	C2	C1	C6	-122.8(2)
Rh1	C2	C3	C4	-60.6(3)
Rh1	C2	C3	C7	119.8(2)
Rh1	C2	C15	C16	-1.4(5)
Rh1	C2	C15	C20	177.5(4)
Rh1	C3	C2	C1	64.5(2)
Rh1	C3	C2	C15	-132.1(2)
Rh1	C3	C4	C5	-56.3(2)
Rh1	C3	C4	C10	122.9(2)
Rh1	C3	C7	C8	-91.3(4)
Rh1	C3	C7	C11	88.1(4)
Rh1	C4	C3	C2	57.3(2)
Rh1	C4	C3	C7	-122.9(2)
Rh1	C4	C5	C1	-65.2(2)
Rh1	C4	C10	C9	91.1(4)
Rh1	C4	C10	C14	-88.8(4)
Rh1	C5	C1	C2	-59.4(3)
Rh1	C5	C1	C6	122.5(2)
Rh1	C5	C4	C3	59.6(2)
Rh1	C5	C4	C10	-119.6(3)
Rh2	C1B	C2B	C3B	-65.8(2)
Rh2	C1B	C2B	C15B	127.6(2)
Rh2	C1B	C5B	C4B	66.7(2)
Rh2	C2B	C1B	C5B	59.3(2)
Rh2	C2B	C1B	C6B	-123.6(3)
Rh2	C2B	C3B	C4B	-60.1(2)
Rh2	C2B	C3B	C7B	120.0(3)
Rh2	C2B	C15B	C16B	16.4(5)
Rh2	C2B	C15B	C20B	-167.4(4)
Rh2	C3B	C2B	C1B	63.6(2)
Rh2	C3B	C2B	C15B	-130.8(2)
Rh2	C3B	C4B	C5B	-57.0(2)
Rh2	C3B	C4B	C10B	123.2(2)
Rh2	C3B	C7B	C8B	-90.8(4)
Rh2	C3B	C7B	C11B	88.2(3)
Rh2	C4B	C3B	C2B	57.7(2)
Rh2	C4B	C3B	C7B	-122.4(2)
Rh2	C4B	C5B	C1B	-64.6(2)
Rh2	C4B	C10B	C9B	90.4(4)
Rh2	C4B	C10B	C14B	-91.2(4)
Rh2	C5B	C1B	C2B	-59.7(2)
Rh2	C5B	C1B	C6B	123.2(3)
Rh2	C5B	C4B	C3B	59.8(2)
Rh2	C5B	C4B	C10B	-120.4(3)
C1	C2	C3	C4	3.9(4)

Atom	Atom	Atom	Atom	Angle/˚
C1	C2	C3	C7	-175.8(3)
C1	C2	C15	C16	-94.6(5)
C1	C2	C15	C20	84.3(4)
C1	C5	C4	C3	-5.7(4)
C1	C5	C4	C10	175.2(3)
C1B	C2B	C3B	C4B	3.5(3)
C1B	C2B	C3B	C7B	-176.4(3)
C1B	C2B	C15B	C16B	-76.3(5)
C1B	C2B	C15B	C20B	99.8(4)
C1B	C5B	C4B	C3B	-4.8(4)
C1B	C5B	C4B	C10B	175.0(3)
C2	C1	C5	C4	8.2(4)
C2	C3	C4	C5	1.0(3)
C2	C3	C4	C10	-179.8(3)
C2	C3	C7	C8	178.1(4)
C2	C3	C7	C11	-2.5(5)
C2	C15	C16	C17	-179.9(5)
C2	C15	C20	C19	-179.7(5)
C2B	C1B	C5B	C4B	7.0(4)
C2B	C3B	C4B	C5B	0.7(3)
C2B	C3B	C4B	C10B	-179.1(3)
C2B	C3B	C7B	C8B	176.9(5)
C2B	C3B	C7B	C11B	-4.1(5)
C2B	C15B	C16B	C17B	177.4(4)
C2B	C15B	C20B	C19B	-177.5(4)
C3	C2	C1	C5	-7.4(4)
C3	C2	C1	C6	170.8(3)
C3	C2	C15	C16	104.1(5)
C3	C2	C15	C20	-77.0(5)
C3	C4	C10	C9	1.1(5)
C3	C4	C10	C14	-178.8(4)
C3	C7	C8	C9	2.1(5)
C3	C7	C8	C12	-176.6(4)
C3B	C2B	C1B	C5B	-6.5(4)
C3B	C2B	C1B	C6B	170.6(3)
C3B	C2B	C15B	C16B	120.0(5)
C3B	C2B	C15B	C20B	-63.8(5)
C3B	C4B	C10B	C9B	0.6(5)
C3B	C4B	C10B	C14B	179.1(4)
C3B	C7B	C8B	C9B	3.7(5)
C3B	C7B	C8B	C12B	-175.5(4)
C4	C3	C2	C15	167.4(3)
C4	C3	C7	C8	-1.5(4)
C4	C3	C7	C11	177.9(4)
C4	C5	C1	C6	-169.9(3)
C4	C10	C9	C8	-0.6(5)
C4	C10	C9	C13	-179.5(4)
C4B	C3B	C2B	C15B	169.1(3)
C4B	C3B	C7B	C8B	-3.0(4)
C4B	C3B	C7B	C11B	175.9(4)
C4B	C5B	C1B	C6B	-170.1(3)
C4B	C10B	C9B	C8B	-0.0(5)
C4B	C10B	C9B	C13B	-178.1(6)
C5	C1	C2	C15	-172.3(3)
C5	C4	C3	C7	-179.3(3)
C5	C4	C10	C9	-179.9(5)
C5	C4	C10	C14	0.2(5)

Atom	Atom	Atom	Atom	Angle/°
C5B	C1B	C2B	C15B	-173.1(3)
C5B	C4B	C3B	C7B	-179.4(3)
C5B	C4B	C10B	C9B	-179.1(5)
C5B	C4B	C10B	C14B	-0.6(5)
C6	C1	C2	C15	5.8(5)
C6B	C1B	C2B	C15B	4.0(5)
C7	C3	C2	C15	-12.3(5)
C7	C3	C4	C10	-0.0(4)
C7	C8	C9	C10	-1.1(5)
C7	C8	C9	C13	177.9(4)
C7B	C3B	C2B	C15B	-10.8(5)
C7B	C3B	C4B	C10B	0.9(4)
C7B	C8B	C9B	C10B	-2.2(5)
C7B	C8B	C9B	C13B	175.9(6)
C8	C9	C10	C14	179.3(4)
C8B	C9B	C10B	C14B	-178.4(4)
C9	C8	C7	C11	-177.3(4)
C9B	C8B	C7B	C11B	-175.2(4)
C10	C9	C8	C12	177.6(4)
C10B	C9B	C8B	C12B	176.9(5)
C11	C7	C8	C12	4.0(5)
C11B	C7B	C8B	C12B	5.6(5)
C12	C8	C9	C13	-3.4(4)
C12B	C8B	C9B	C13B	-5.0(6)
C13	C9	C10	C14	0.4(5)
C13B	C9B	C10B	C14B	3.5(7)
C15	C16	C17	C18	-0.0(6)
C15	C20	C19	C18	-0.9(6)
C15B	C16B	C17B	C18B	-0.5(5)
C15B	C20B	C19B	C18B	0.3(6)
C16	C15	C20	C19	-0.8(5)
C16	C17	C18	C19	-1.7(7)
C16B	C15B	C20B	C19B	-1.2(5)
C16B	C17B	C18B	C19B	-0.5(6)
C17	C16	C15	C20	1.2(6)
C17	C18	C19	C20	2.1(6)
C17B	C16B	C15B	C20B	1.3(6)
C17B	C18B	C19B	C20B	0.6(6)

Table 6: Hydrogen Fractional Atomic Coordinates (×10[4]) and Equivalent Isotropic Displacement Parameters (Å[2]×10[3]) for EMH-0663. U_{eq} is defined as 1/3 of the trace of the orthogonalised U_{ij}.

Atom	x	y	z	U_{eq}
H6a	3743(9)	7657(10)	1260(20)	45.4(14)
H6b	3730(10)	6765(8)	1390(20)	45.4(14)
H6c	3265.2(19)	7265(18)	2456(5)	45.4(14)
H6Ba	7390(15)	5815(3)	177(11)	45.5(14)
H6Bb	6855(6)	6283(7)	-800(30)	45.5(14)
H6Bc	7655(10)	6152(9)	-1313(19)	45.5(14)
H11a	5135(15)	8978(2)	4254(14)	42.8(14)
H11b	5028(13)	9007(2)	5950(20)	42.8(14)
H11c	5810(3)	9006(2)	5290(30)	42.8(14)
H11d	9456(4)	7535(13)	2775(15)	35.4(12)
H11e	8964(11)	7806(9)	4068(9)	35.4(12)
H11f	8782(9)	7048(5)	3240(20)	35.4(12)

Atom	x	y	z	U_{eq}
H12a	6018(14)	7839(11)	8018(8)	54.3(16)
H12b	6577(3)	8092(16)	6828(18)	54.3(16)
H12c	5900(11)	8603(6)	7160(20)	54.3(16)
H12d	8971(11)	8910(15)	3949(6)	56.5(16)
H12e	9540(3)	8808(13)	2700(30)	56.5(16)
H12f	9038(12)	9529(3)	2720(30)	56.5(16)
H13a	6602(8)	6934(10)	7160(30)	56.3(17)
H13b	5920(8)	6629(17)	7987(10)	56.3(17)
H13c	6261(14)	6147(7)	6721(18)	56.3(17)
H13d	8611(16)	9932(3)	900(50)	126(4)
H13e	7763(15)	9960(3)	930(50)	126(4)
H13f	8200(30)	9919(3)	2392(9)	126(4)
H14a	5848(8)	5701(2)	4770(30)	53.6(16)
H14b	5380(17)	5666(2)	6195(6)	53.6(16)
H14c	5002(10)	5682(3)	4670(30)	53.6(16)
H14d	6948(11)	9362(14)	230(5)	55.4(17)
H14e	7566(3)	9410(13)	-940(20)	55.4(17)
H14f	7033(13)	8713(3)	-930(20)	55.4(17)
H16	4813(2)	8800(2)	1478(5)	40.6(12)
H16B	7631(2)	5867(2)	2733(4)	31.0(10)
H17	4286(2)	9905(3)	1178(6)	49.2(14)
H17B	8277(2)	4880(2)	3356(4)	33.8(11)
H18	3419(2)	10268(3)	2648(5)	44.9(13)
H18B	9385(2)	4769(2)	2570(5)	37.5(11)
H19	3043(3)	9495(3)	4343(6)	49.9(15)
H19B	9839(2)	5633(3)	1146(5)	43.8(14)
H20	3579(2)	8395(2)	4669(5)	41.1(12)
H20B	9192(2)	6633(2)	517(5)	35.0(11)
H5	4550(30)	6130(30)	3100(60)	59(15)
H5B	6920(30)	7590(30)	-780(60)	65(17)

Table 7: Solvent masking (PLATON/SQUEEZE) information for EMH-0663.

No	x	y	z	V	e	Content
1	0.000	0.000	-0.044	130.5	52.4	1C1,1H2,1Cl2
2	0.083	0.596	0.048	11.3	0.0	?
3	0.500	0.500	0.044	130.5	52.5	1C1,1H2,1Cl2
4	0.417	0.096	-0.048	11.3	1.0	?
5	0.583	0.904	0.952	11.3	0.0	?
6	0.917	0.404	0.048	11.3	0.0	?

10:

Experimental. Single colorless needle-shaped crystals of EMH-0823 were recrystallized from a mixture of heptane and chloroform by slow evaporation. A suitable crystal with dimensions 0.44 × 0.10 × 0.05 mm^3 was selected and mounted on a loop with IVH oil on a XtaLAB Synergy, Dualflex, HyPix diffractometer. The crystal was kept at a steady T = 100.00(10) K during data collection. The structure was solved with ShelXT (Sheldrick, 2015) and Olex2 1.5-alpha (Dolomanov et al., 2009. The structure was refined with olex2.refine 1.5-alpha (Bourhis et al., 2015) using full matrix least squares minimisation on $\boldsymbol{F^2}$.

Crystal Data. $C_{13}H_{16}BrNO_2$, M_r = 298.181, orthorhombic, $P2_12_12_1$ (No. 19), a = 4.89174(4) Å, b = 12.75435(11) Å, c = 21.7609(2) Å, $\alpha = \beta = \gamma$ = 90°, V = 1357.68(2) Å^3, T = 100.00(10) K, Z = 4, Z' = 1, μ(Cu K$_\alpha$) = 4.058, 29968 reflections measured, 2825 unique (R_{int} = 0.0256) which were used in all calculations. The final wR_2 was 0.0232 (all data) and R_1 was 0.0114 (I≥2 σ(I)).

**Compound**	CCDC 2454911
Formula	$C_{13}H_{16}BrNO_2$
$D_{calc.}$/ g cm^{-3}	1.459
μ/mm^{-1}	4.058
Formula Weight	298.181
Color	colorless
Shape	needle-shaped
Size/mm^3	0.44×0.10×0.05
T/K	100.00(10)
Crystal System	orthorhombic
Flack Parameter	-0.017(4)
Hooft Parameter	-0.017(4)
Space Group	$P2_12_12_1$
a/Å	4.89174(4)
b/Å	12.75435(11)
c/Å	21.7609(2)
α/°	90
β/°	90
γ/°	90
V/Å^3	1357.68(2)
Z	4
Z'	1
Wavelength/Å	1.54184
Radiation type	Cu K$_\alpha$
Θ_{min}/°	4.02
Θ_{max}/°	77.27
Measured Refl's.	29968
Indep't Refl's	2825
Refl's I≥2 σ(I)	2787
R_{int}	0.0256
Parameters	285
Restraints	129
Largest Peak	0.3476
Deepest Hole	-0.2411
GooF	1.0439
wR_2 (all data)	0.0232
wR_2	0.0231
R_1 (all data)	0.0117
R_1	0.0114

Structure Quality Indicators

**Reflections:**	d min (CuKα) 2Θ=154.5° 0.79	I/σ(I) 92.4	Rint m=10.54 2.56%	Full 135.4° 99.3	
**Refinement:**	Shift -0.004	Max Peak 0.3	Min Peak -0.2	GooF 1.044	Hooft -.017(4)

A colourless needle-shaped-shaped crystal with dimensions 0.44 × 0.10 × 0.05 mm^3 was mounted on a loop with IVH oil. Data were collected using a XtaLAB Synergy, Dualflex, HyPix diffractometer operating at T = 100.00(10) K.

Data were measured using ω scans with Cu K_α radiation. The diffraction pattern was indexed and the total number of runs and images was based on the strategy calculation from the program CrysAlisPro system (CCD 44.57a 64-bit (release 20-06-2024)). The maximum resolution that was achieved was Θ = 77.27° (0.79 Å).

The unit cell was refined using CrysAlisPro 1.171.44.57a (Rigaku OD, 2024) on 25038 reflections, 84% of the observed reflections.

Data reduction, scaling and absorption corrections were performed using CrysAlisPro 1.171.44.57a (Rigaku OD, 2024). The final completeness is 99.25 % out to 77.27° in Θ. A numerical absorption correction based on gaussian integration over a multifaceted crystal model was performed using CrysAlisPro 1.171.42.74a (Rigaku Oxford Diffraction, 2022). An empirical absorption correction using spherical harmonics, implemented in SCALE3 ABSPACK scaling algorithm was also applied. The absorption coefficient μ of this material is 4.058 mm^{-1} at this wavelength (λ = 1.54184Å) and the minimum and maximum transmissions are 0.351 and 1.000.

The structure was solved and the space group $P2_12_12_1$ (# 19) determined by the ShelXT (Sheldrick, 2015) structure solution program and refined by full matrix least squares minimisation on $\boldsymbol{F}^2$ using version of olex2.refine 1.5-alpha (Bourhis et al., 2015). All atoms, even hydrogen atoms, were refined anisotropically. Hydrogen atom positions were located from the electron densities and freely refined using Hirshfeld scattering factors. Refinement was by using NoSpherA2, an implementation of non-spherical atom-form-factors (F. Kleemiss, H. Puschmann, O. Dolomanov, S.Grabowsky - https://doi.org/10.1039/D0SC05526C – 2020). SOFTWARE: ORCA 5.0 PARTITIONING: NoSpherA2 INT ACCURACY: Normal METHOD: PBE BASIS SET: def2-SVP CHARGE: 0 MULTIPLICITY: 1 DATE: 2025-05-19_18-04-55

There is a single formula unit in the asymmetric unit, which is represented by the reported sum formula. In other words: Z is 4 and Z' is 1. The moiety formula is C13 H16 Br N O2.

The Flack parameter is very close to zero: the refined value to -0.02(1). The absolute structure was determined using Bayesian statistics on Bijvoet differences and Olex2, resulting in a value of in -0.04(1). The chiral atoms in this structure are: C7(S) and C9(R). Note: The Flack parameter is used to determine chirality of the crystal studied, the value should be near 0, a value of 1 means that the stereochemistry is wrong and the model should be inverted. A value of 0.5 means that the crystal consists of a racemic mixture of the two enantiomers.

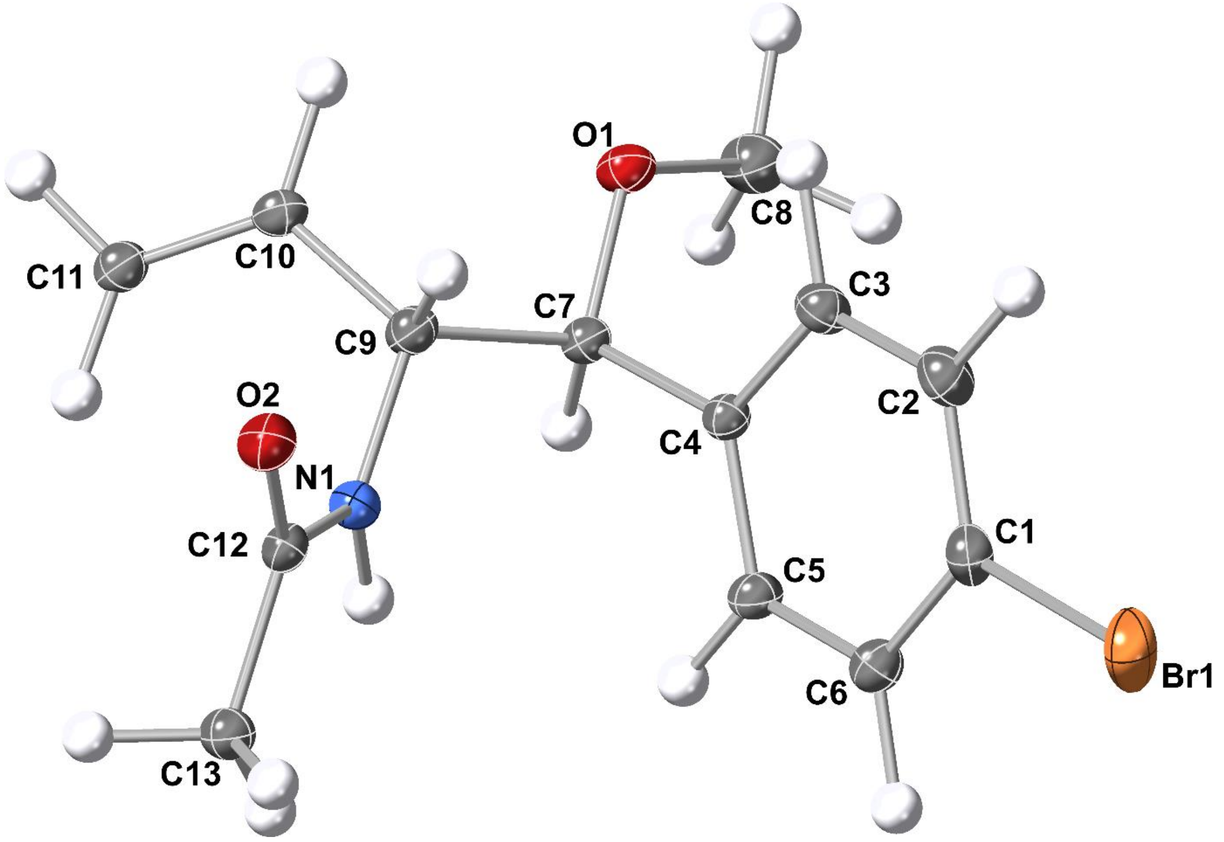

Figure 5 Thermal ellipsoidal representation of the crystal structure. The chiral atoms in this structure are: C7(S) and C9(R). The ellipses are shown at the 35% probability level, and the hydrogen atoms are shown as spheres.

Data Plots: Diffraction Data

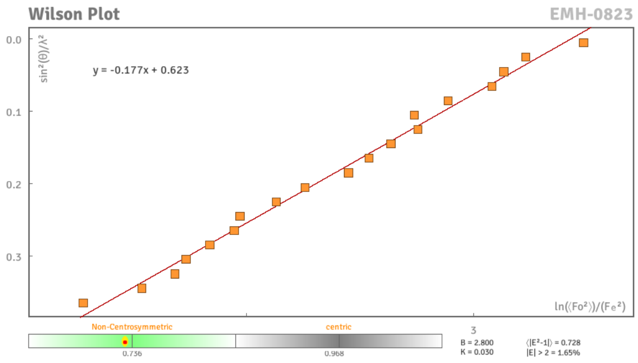

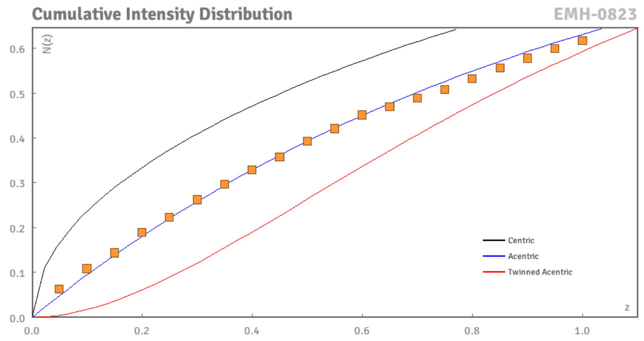

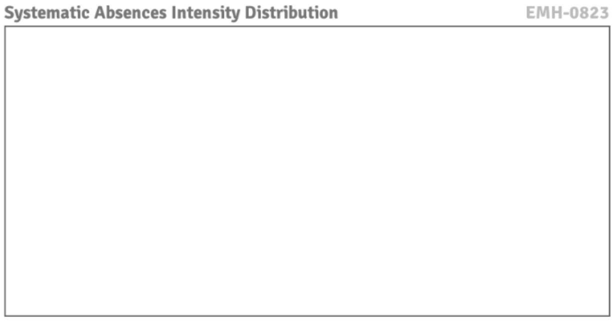

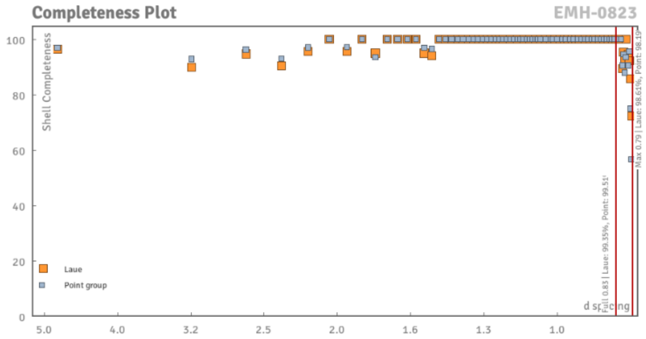

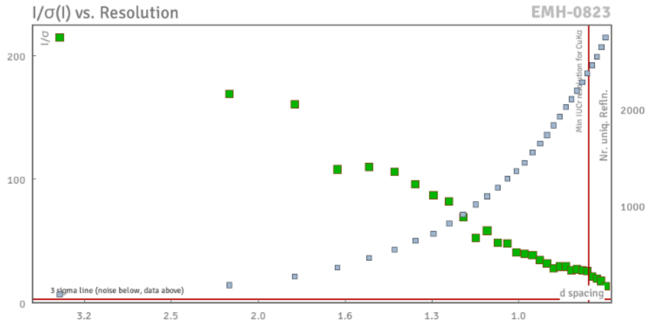

Data Plots: Refinement and Data

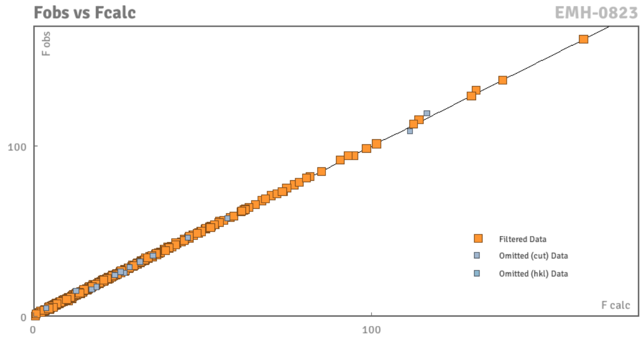

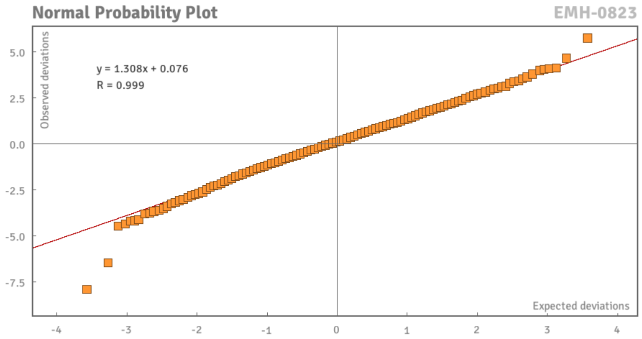

Reflection Statistics

Total reflections (after filtering)	29778	Unique reflections	2826
Completeness	0.982	Mean I/σ	62.07
hkl_{max} collected	(5, 16, 27)	hkl_{min} collected	(-6, -15, -27)
hkl_{max} used	(6, 16, 27)	hkl_{min} used	(-6, 0, 0)
Lim d_{max} collected	100.0	Lim d_{min} collected	0.77
d_{max} used	11.0	d_{min} used	0.79
Friedel pairs	3185	Friedel pairs merged	0
Inconsistent equivalents	4	R_{int}	0.0255
R_{sigma}	0.0108	Intensity transformed	0
Omitted reflections	0	Omitted by user (OMIT hkl)	190
Multiplicity	(2081, 1609, 1403, 1202, 835, 709, 357, 183, 98, 56, 46, 46, 20, 26, 8, 1)	Maximum multiplicity	28
Removed systematic absences	0	Filtered off (Shel/OMIT)	0

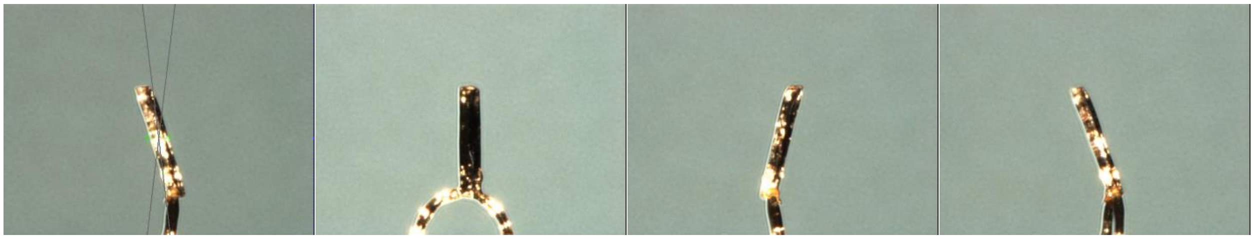

Table 8: Fractional Atomic Coordinates (×10[4]) and Equivalent Isotropic Displacement Parameters (Å[2]×10[3]) for EMH-0823. U_{eq} is defined as 1/3 of the trace of the orthogonalised U_{ij}.

Atom	x	y	z	U_{eq}
Br1	6267.2(6)	9084.85(19)	4285.98(12)	42.38(4)
O2	2614.0(12)	3909.9(6)	4587.0(3)	27.92(16)
O1	7248.8(14)	4568.1(5)	2577.9(3)	27.82(15)
N1	6894.7(16)	3848.3(6)	4195.2(4)	21.01(17)
C1	6873(2)	7742.9(7)	3937.6(5)	27.7(2)
C2	5270(2)	7418.3(8)	3449.2(5)	31.8(2)
C3	5679(2)	6415.6(8)	3210.1(4)	27.4(2)
C4	7670.2(17)	5754.0(7)	3452.6(4)	20.56(18)
C5	9277.6(19)	6109.0(7)	3937.9(4)	25.9(2)
C6	8883(2)	7103.8(7)	4186.2(4)	29.1(2)
C7	7955(2)	4644.0(8)	3207.7(4)	21.78(19)
C8	9295(2)	4972.0(9)	2181.5(5)	35.6(3)
C9	6039(2)	3894.4(7)	3558.9(4)	25.90(19)
C10B	5615(3)	2859.4(10)	3239.6(6)	28.8(3)
C11B	6071(3)	1942.1(10)	3513.9(7)	28.7(3)
C12	5107.6(18)	3851.4(7)	4663.4(4)	19.33(18)
C13	6353(3)	3761.5(9)	5292.1(5)	27.0(2)
C11A	5094(12)	1969(4)	3339(3)	28.7(3)
C10A	6875(10)	2757(3)	3384(2)	28.8(3)

Table 9: Anisotropic Displacement Parameters (×10[4]) for EMH-0823. The anisotropic displacement factor exponent takes the form: $-2\pi^2[h^2a^{*2} \times U_{11} + \ldots + 2hka^* \times b^* \times U_{12}]$

Atom	U_{11}	U_{22}	U_{33}	U_{23}	U_{13}	U_{12}
Br1	63.78(8)	21.66(5)	41.69(6)	4.12(6)	18.12(6)	-1.05(5)
O2	14.2(3)	32.9(4)	36.7(4)	0.6(3)	1.9(3)	0.4(3)
O1	27.5(3)	34.4(4)	21.6(3)	-5.6(3)	1.0(3)	-4.1(3)
N1	15.9(4)	24.5(4)	22.6(4)	1.7(3)	1.3(3)	0.1(3)
C1	34.2(6)	21.2(4)	27.5(5)	1.4(4)	6.3(4)	2.4(4)
C2	35.7(6)	27.5(5)	32.3(5)	8.5(4)	-1.3(4)	3.7(4)
H2	73(7)	43(5)	75(8)	30(3)	-36(4)	-12(3)
C3	27.7(6)	27.9(5)	26.6(5)	4.4(4)	-4.3(4)	1.9(4)
H3	45(6)	46(6)	46(5)	15(3)	-22(3)	-11(3)
C4	18.7(4)	21.3(5)	21.7(4)	-0.3(3)	-0.6(3)	-0.1(3)
C5	24.4(5)	24.4(5)	28.8(5)	1.3(4)	-6.7(4)	-2.6(4)
H5	44(5)	33(4)	48(6)	12(2)	-25(3)	-13(3)
C6	34.4(5)	23.9(4)	28.9(5)	-0.6(4)	-3.1(4)	-3.0(4)
H6	60(7)	41(6)	55(6)	12(3)	-27(3)	-19(3)
C7	21.0(5)	23.0(5)	21.3(4)	-1.7(4)	0.2(4)	-1.9(4)
H7	18(6)	63(9)	41(7)	16(6)	0(6)	-10(6)
C8	34.9(6)	46.7(6)	25.1(5)	-5.0(5)	6.6(4)	-1.3(4)
H8A	61(6)	66(6)	30(2)	-7(3)	0.4(14)	-3.0(14)

Atom	U_{11}	U_{22}	U_{33}	U_{23}	U_{13}	U_{12}
H8B	56(6)	50(2)	44(6)	-8.7(13)	8(3)	-4.3(13)
H8C	41(3)	63(5)	45(6)	0.8(17)	4.8(18)	0(3)
C9	26.9(5)	27.6(5)	23.2(4)	-6.1(4)	0.1(4)	-1.8(3)
C10B	35.2(8)	28.6(5)	22.7(7)	-5.9(4)	-5.1(5)	-2.2(4)
C11B	29.8(8)	24.5(5)	31.8(8)	-1.1(4)	-5.7(5)	-2.1(4)
C12	14.0(4)	18.8(5)	25.2(4)	0.3(3)	2.0(3)	-1.6(3)
C13	25.5(5)	31.6(5)	23.9(5)	-2.9(5)	0.8(5)	0.9(4)
H13A	64(11)	80(12)	55(10)	16(9)	-18(8)	-26(9)
H13B	87(12)	72(10)	50(9)	-72(10)	-25(9)	36(8)
H13C	57(10)	79(11)	27(7)	-12(10)	-14(7)	8(7)
C11A	29.8(8)	24.5(5)	31.8(8)	-1.1(4)	-5.7(5)	-2.1(4)
C10A	35.2(8)	28.6(5)	22.7(7)	-5.9(4)	-5.1(5)	-2.2(4)
H1	40(8)	41(8)	38(7)	-5(7)	20(8)	18(6)

Table 10: Bond Lengths in Å for EMH-0823.

Atom	Atom	Length/Å
Br1	C1	1.8952(10)
O2	C12	1.2334(10)
O1	C7	1.4167(11)
O1	C8	1.4180(12)
N1	C9	1.4477(12)
N1	C12	1.3424(11)
N1	H1	1.012(13)
C1	C2	1.3840(15)
C1	C6	1.3872(14)
C2	H2	1.088(13)
C2	C3	1.3952(14)
C3	H3	1.120(11)
C3	C4	1.3924(13)
C4	C5	1.3924(12)
C4	C7	1.5190(13)
C5	H5	1.071(10)
C5	C6	1.3925(13)
C6	H6	1.102(12)
C7	H7	1.126(11)
C7	C9	1.5414(13)

Atom	Atom	Length/Å
C8	H8A	1.0970
C8	H8B	1.0970
C8	H8C	1.0970
C9	H9A	1.1180
C9	H9B	1.094(14)
C9	C10B	1.5061(15)
C9	C10A	1.554(3)
C10B	H10B	1.070(14)
C10B	C11B	1.3323(17)
C11B	H11A	1.068(4)
C11B	H11B	1.070(4)
C12	C13	1.5022(14)
C13	H13A	1.068(14)
C13	H13B	1.076(12)
C13	H13C	1.038(14)
C11A	H11C	1.1020
C11A	H11D	1.1020
C11A	C10A	1.334(4)
C10A	H10A	1.1030

Table 11: Bond Angles in ° for EMH-0823.

Atom	Atom	Atom	Angle/°
C8	O1	C7	113.06(7)
C12	N1	C9	122.51(8)
H1	N1	C9	118.5(7)
H1	N1	C12	119.0(7)
C2	C1	Br1	119.26(8)
C6	C1	Br1	119.04(8)
C6	C1	C2	121.69(9)
H2	C2	C1	120.4(7)
C3	C2	C1	118.64(9)
C3	C2	H2	120.9(7)
H3	C3	C2	119.7(6)
C4	C3	C2	120.97(9)

Atom	Atom	Atom	Angle/°
C4	C3	H3	119.4(6)
C5	C4	C3	119.03(9)
C7	C4	C3	119.73(8)
C7	C4	C5	121.16(8)
H5	C5	C4	119.9(5)
C6	C5	C4	120.82(9)
C6	C5	H5	119.3(5)
C5	C6	C1	118.84(9)
H6	C6	C1	121.2(6)
H6	C6	C5	120.0(6)
C4	C7	O1	112.38(8)
H7	C7	O1	109.6(6)

Atom	Atom	Atom	Angle/˚
H7	C7	C4	109.8(6)
C9	C7	O1	106.80(7)
C9	C7	C4	110.40(7)
C9	C7	H7	107.7(6)
H8A	C8	O1	109.5
H8B	C8	O1	109.5
H8B	C8	H8A	109.5
H8C	C8	O1	109.5
H8C	C8	H8A	109.5
H8C	C8	H8B	109.5
C7	C9	N1	108.89(8)
H9A	C9	N1	114.09(5)
H9A	C9	C7	114.09(5)
H9B	C9	N1	103.9(7)
H9B	C9	C7	105.8(7)
H9B	C9	H9A	18.4(7)
C10B	C9	N1	116.45(9)
C10B	C9	C7	113.50(8)
C10B	C9	H9A	88.86(7)
C10B	C9	H9B	107.3(7)
C10A	C9	N1	96.9(2)
C10A	C9	C7	107.34(19)
C10A	C9	H9A	114.09(19)

Atom	Atom	Atom	Angle/˚
C10A	C9	H9B	132.1(7)
C10A	C9	C10B	26.55(17)
H10B	C10B	C9	116.1(8)
C11B	C10B	C9	122.68(13)
C11B	C10B	H10B	121.2(8)
H11A	C11B	C10B	121.7(7)
H11B	C11B	C10B	119.4(7)
H11B	C11B	H11A	118.6(10)
N1	C12	O2	122.82(9)
C13	C12	O2	121.90(9)
C13	C12	N1	115.27(9)
H13A	C13	C12	108.9(7)
H13B	C13	C12	108.5(7)
H13B	C13	H13A	107.4(11)
H13C	C13	C12	112.7(6)
H13C	C13	H13A	110.4(11)
H13C	C13	H13B	108.8(12)
H11D	C11A	H11C	120.0
C10A	C11A	H11C	120.0
C10A	C11A	H11D	120.0
C11A	C10A	C9	123.4(4)
H10A	C10A	C9	118.32(18)
H10A	C10A	C11A	118.3(3)

Table 12: Torsion Angles in ˚ for EMH-0823.

Atom	Atom	Atom	Atom	Angle/˚
Br1	C1	C2	C3	-177.97(8)
Br1	C1	C6	C5	178.52(7)
O2	C12	N1	C9	-1.15(12)
O1	C7	C4	C3	31.53(9)
O1	C7	C4	C5	-151.70(8)
O1	C7	C9	N1	173.20(7)
O1	C7	C9	C10B	41.74(10)
O1	C7	C9	C10A	69.4(2)
N1	C9	C7	C4	-64.35(8)
N1	C9	C10B	C11B	-2.01(13)
N1	C9	C10A	C11A	101.3(4)
C1	C2	C3	C4	-0.49(12)
C1	C6	C5	C4	-0.64(11)
C2	C1	C6	C5	-0.34(12)
C2	C3	C4	C5	-0.45(12)
C2	C3	C4	C7	176.39(9)
C3	C2	C1	C6	0.90(12)
C3	C4	C5	C6	1.03(11)
C3	C4	C7	C9	-87.59(9)
C4	C7	O1	C8	75.74(9)
C4	C7	C9	C10B	164.19(9)
C4	C7	C9	C10A	-168.2(2)
C5	C4	C7	C9	89.18(9)
C6	C5	C4	C7	-175.77(9)
C7	C9	N1	C12	138.35(7)
C7	C9	C10B	C11B	125.63(12)
C7	C9	C10A	C11A	-146.4(4)
C8	O1	C7	C9	-163.06(9)

Atom	Atom	Atom	Atom	Angle/°
C9	N1	C12	C13	177.95(8)
C10B	C9	N1	C12	-91.78(10)
C10B	C9	C10A	C11A	-38.3(6)
C11B	C10B	C9	C10A	43.9(4)
C12	N1	C9	C10A	-110.6(2)

Table 13: Hydrogen Fractional Atomic Coordinates (×10[4]) and Equivalent Isotropic Displacement Parameters ($Å^2 \times 10^3$) for EMH-0823. U_{eq} is defined as 1/3 of the trace of the orthogonalised U_{ij}.

Atom	x	y	z	U_{eq}
H2	3690(30)	7928(9)	3266(6)	64(4)
H3	4400(20)	6140(9)	2815(5)	46(3)
H5	10830(20)	5612(8)	4126(5)	41(3)
H6	10160(30)	7370(10)	4574(6)	52(4)
H7	10110(20)	4361(10)	3277(5)	41(3)
H8A	8661(7)	4864(5)	1702.1(5)	52(3)
H8B	9584(10)	5810.2(15)	2274(2)	50(3)
H8C	11222(4)	4554(4)	2262(2)	49(3)
H9A	3816(2)	4066.0(7)	3495.0(4)	31.1(2)
H9B	4050(30)	4288(10)	3577(6)	31.1(2)
H10B	4900(30)	2899(11)	2776(7)	34.6(4)
H11A	6670(30)	1895(10)	3985(3)	34.5(4)
H11B	5630(30)	1231(6)	3273(5)	34.5(4)
H13A	5440(30)	4334(12)	5586(6)	66(4)
H13B	5860(30)	3002(11)	5476(6)	70(5)
H13C	8460(30)	3848(11)	5288(5)	54(4)
H11C	2906(12)	2108(4)	3427(3)	34.5(4)
H11D	5811(12)	1178(4)	3213(3)	34.5(4)
H10A	9051(10)	2593(3)	3293(2)	34.6(4)
H1	8920(30)	3812(9)	4289(5)	40(3)

Table 14: Atomic Occupancies for all atoms that are not fully occupied in EMH-0823.

Atom	Occupancy
H9A	0.218(2)
H9B	0.782(2)
C10B	0.782(2)
H10B	0.782(2)
C11B	0.782(2)
H11A	0.782(2)
H11B	0.782(2)
C11A	0.218(2)
H11C	0.218(2)
H11D	0.218(2)
C10A	0.218(2)
H10A	0.218(2)

Citations

CrysAlisPro (ROD), Rigaku Oxford Diffraction, Poland (?).

CrysAlisPro Software System, Rigaku Oxford Diffraction, (2024).

L.J. Bourhis and O.V. Dolomanov and R.J. Gildea and J.A.K. Howard and H. Puschmann, The Anatomy of a Comprehensive Constrained, Restrained, Refinement Program for the Modern Computing Environment - Olex2 Disected, *Acta Cryst. A*, (2015), **A71**, 59-71.

O.V. Dolomanov and L.J. Bourhis and R.J. Gildea and J.A.K. Howard and H. Puschmann, Olex2: A complete structure solution, refinement and analysis program, *J. Appl. Cryst.*, (2009), **42**, 339-341.

Sheldrick, G.M., ShelXT-Integrated space-group and crystal-structure determination, *Acta Cryst.*, (2015), **A71**, 3-8.

10. References

1. Farr, C.M.B.; Kazerouni, A.M.; Park, B.; Poff, C.D.; Won, J.; Sharp, K.R.; Baik, M-H.; Blakey, S.B. Designing a Planar Chiral Rhodium Indenyl Catalyst for Regio- and Enantioselective Allylic C-H Amidation. *J. Am. Chem. Soc*. **2020**, *142*, 13996-14004.
2. Gross, P.; Im, H.; Laws III, D.; Park, B.; Baik, M-H.; Blakey, S.B. Enantioselective Aziridination of Unactivated Terminal Alkenes Using a Planar Chiral Rh(III) Indenyl Catalyst. *J. Am. Chem. Soc*. **2024**, *146*, 1447-1454.
3. Burg, F.; Rovis, T. Diastereoselective Three-Component 3,4-Amino Oxygenation of 1,3-Dienes Catalyzed by a Cationic Heptamethylindenyl Rhodium(III) Complex. *J. Am. Chem. Soc*. **2021**, *143*, 17964-17969.
4. Burg, F.; Rovis, T. Rh(III)-Catalyzed Intra- and Intermolecular 3,4-Difunctionalization of 1,3-Dienes via Rh(III)-π-Allyl Amidation with 1,4,2-Dioxazolones. *ACS Catal*. **2022**, *12*, 9690-9697.
5. Li, X.; Song, H.; Yu, S.; Mi, R.; Li, X-X. Rhodium-Catalyzed Enantioselective 1,4-Oxyamination of Conjugated gem-Difluorodienes via Coupling with Carboxylic Acids and Dioxazolones. *Angew. Chem. Int. Ed.* **2023**, *62*, e202305669.
6. Adegboyega, A. K.; Son, J. Reaction of Dioxazolones with Boronic Acids: Copper-Mediated Synthesis of *N*-Aryl Amides via *N*-Acyl Nitrenes. *Org. Lett.* **2022,** *24* (27), 4925-4929.
7. Burman, J. S.; Harris, R. J.; Farr, C. M. B.; Bacsa, J.; Blakey, S. B. Rh(III) and Ir(III)Cp* Complexes Provide Complementary Regioselectivity Profiles in Intermolecular Allylic C-H Amidation Reactions. *ACS Catal.* **2019,** *9* (6), 5474-5479.
